# Genetic associations of leisure sedentary behaviors and the risk of 15 site‐specific cancers: A Mendelian randomization study

**DOI:** 10.1002/cam4.5974

**Published:** 2023-05-06

**Authors:** Jinwei Chen, Kaibin Yang, Youyu Qiu, Weijie Lai, Sifan Qi, Gaoyuan Wang, Lin Chen, Kunpeng Li, Dan Zhou, Qing Liu, Linglong Tang, Xu Liu, Xiaojing Du, Rui Guo, Jun Ma

**Affiliations:** ^1^ Department of Radiation Oncology Sun Yat‐sen University Cancer Center, State Key Laboratory of Oncology in South China, Collaborative Innovation Center for Cancer Medicine, Guangdong Key Laboratory of Nasopharyngeal Carcinoma Diagnosis and Therapy Guangzhou P. R. China; ^2^ Zhongshan School of Medicine Sun Yat‐sen University Guangzhou P. R. China; ^3^ Department of Radiation Oncology Sixth Affiliated Hospital, Kunming Medical University Yuxi Yunan China; ^4^ Department of Medical Statistics and Epidemiology, School of Public Health Sun Yat‐sen University Guangzhou P. R. China

**Keywords:** cancer risk factors, epidemiology and prevention, genome‐wide association, women's cancer

## Abstract

**Background and Aims:**

Leisure sedentary behavior (LSB) is associated with the risk of cancer, but the causal relationship between them has not been clarified. The aim of this study was to assess the potential causal association between LSB and risk of 15 site‐specific cancers.

**Methods:**

The causal association between LSB and cancer were assessed with univariate Mendelian randomization (UVMR) and multivariate Mendelian randomization (MVMR). 194 SNPs associated with LSB (from the UK Biobank 408,815 individuals) were adopted as the instrument variables. Sensitivity analyses were performed to ensure the robustness of the results.

**Results:**

UVMR analysis revealed that television watching significantly increased the risk of endometrial cancer (OR = 1.29, 95% CI = 1.02–1.64, *p* = 0.04) (mainly the endometrioid histology [OR = 1.28, 95% CI = 1.02–1.60, *p* = 0.031])，breast cancer (OR = 1.16, 95% CI = 1.04–1.30, *p* = 0.007) (both ER+ breast cancer [OR = 1.17, 95% CI = 1.03–1.33, *p* = 0.015], and ER− breast cancer [OR = 1.55, 95% CI = 1.26–1.89, *p* = 2.23 × 10^−5^]). Although causal association was not found between television watching and ovarian cancer, it was seen in low grade and low malignant potential serous ovarian cancer (OR = 1.49, 95% CI = 1.07–2.08, *p* = 0.018). However, significant results were not obtained in the UVMR analysis between driving, computer use and the 15 types of cancer. Further MVMR analysis indicated that the above results are independent from most metabolic factors and dietary habits, but mediated by educational attainment.

**Conclusion:**

LSB in form of television watching has independent causal association with the risk of endometrial cancer, breast cancer, and ovarian cancer.

## 1 INTRODUCTION

Cancer is one of the leading causes of death globally and poses a major health threat to humans.^1^ There were an estimated 19.3 million new cancer cases and 10.0 million cancer deaths in 2020.^2^ Premature deaths from cancer have significantly reduced life expectancy in both developing and developed countries.^3^ An increasing volume of evidence has shown the benefits of early prevention of cancer, emphasizing the tremendous potential for the development of good lifestyle habits to prevent the incidence of cancer.^4^, ^5^, ^6^, ^7^

Leisure sedentary behaviors (LSBs) are any activity during which one is seated, reclined, or lying, and does not exert much energy (≤1.5 metabolic equivalents).^8^, ^9^ On average, a British adult spends 5 h a day sedentary,^10^ a French adult sits for 12 h on weekdays,^11^ and an American adult spends 55% of their waking time sedentary, or 7.7 h per day.^12^ Owing to their high prevalence, LSBs have become an important public health issue. High levels of sedentary time have been reported to be associated with an increased risk of many cancers in numerous previous observational studies.^5^, ^13^, ^14^, ^15^, ^16^ Although much observational research has focused on understanding the association between LSB and cancer,^14^, ^17^, ^18^ exploring causation between LSB and cancer is difficult with observational studies, in which confounding factors may affect the conclusions.^5^, ^19^, ^20^, ^21^, ^22^, ^23^

Mendelian randomization (MR) determines whether an observational association between a risk factor and an outcome is causal, by using genetic variants.^24^ Typically, individuals inherit genetic variants that affect a risk factor at birth, and these variants are not confounded by other factors. Hence, differences in outcomes between carriers of variants and those without variants can be attributed to differences in risk factors. As this process is similar to random allocation of treatment in randomized controlled trials, reverse causation and the confounding problems in observational studies can be overcome, thus identifying a causal effect and providing evidence for it.^25^ MR has three core assumptions. First, genetic variants must be associated with exposure, but single nucleotide polymorphisms (SNPs) need not be functional variants that are responsible for SNP‐exposure relationships. Second, genetic variants should not be associated with exposure‐outcome confounders. Third, genetic variants should be associated with outcome only through the exposure being studied.^26^ On the basis of existing studies, we carried out this study in an effort to investigate the causal effects of LSB on the risk of 15 site‐specific cancers using univariable MR (UVMR) and multivariable MR (MVMR) methods.

## 2 METHODS

### 2.1 Genetic variants for leisure sedentary behaviors

The instrumental variables (IVs) for LSBs were derived from a recent genome‐wide association analysis (GWAS) of sedentary behaviors in 408,815 volunteers of European ancestry registered in UK Biobank (Table 1).^27^ During the study, LSB was measured as watching TV, interacting with a computer (excluding work‐related computer use), and driving, according to self‐reported questionnaires. We excluded participants whose sedentary phenotypes were outside of the 99.5% range on the right side of the normal distribution, because of the right‐skewness of sedentary phenotypes. This GWAS analysis revealed 193 independent genetic variants at 169 genetic loci associated with LSB, of which 152 genetic variants at 145 loci were associated with television watching, 37 genetic variants at 36 loci were associated with computer use, and 4 genetic variants at 4 loci were associated with driving (*p* < 1 × 10^−8^).

**TABLE 1:** Overview of the genetic data for leisure sedentary behaviors, covariate variables, and 10 site‐specific cancers^a^.

Trait	GWAS ID	Year	Cohort	Population	PMID	Gender	Sample size	Cases	Controls
Leisure sedentary behaviors	NA	2020	UKB	European	32317632	Female and male	408,815	12,136	396,679
Endometrial cancer	ebi‐a‐GCST006464	2018	ECAC+E2C2+UKB	European	30093612	Female	121,885	12,906	108,979
Endometrial cancer (NEE)	ebi‐a‐GCST006466	2018	ECAC+E2C2+UKB	European	30093612	Female	36,677	1230	35,447
Endometrial cancer (EE)	ebi‐a‐GCST006465	2018	ECAC+E2C2+UKB	European	30093612	Female	54,884	8758	46,126
Breast cancer	ieu‐a‐1126	2017	BCAC+DRIVE	European	29059683	Female	228,951	122,977	105,974
Breast cancer (ER+)	ieu‐a‐1127	2017	BCAC+DRIVE	European	29059683	Female	175,475	69,501	105,974
Breast cancer (ER‐)	ieu‐a‐1128	2017	BCAC+DRIVE	European	29059683	Female	127,442	21,468	105,974
Ovarian cancer	ieu‐a‐1120	2017	OCAC+CIMBA	European	28346442	Female	66,450	25,509	40,941
Ovarian cancer (LGS+LMPS)	ieu‐a‐1229	2017	OCAC+CIMBA	European	28346442	Female	43,907	2966	40,941
Ovarian cancer (HGS)	ieu‐a‐1121	2017	OCAC+CIMBA	European	28346442	Female	53,978	13,037	40,941
Ovarian cancer (MO)	ieu‐a‐1231	2017	OCAC+CIMBA	European	28346442	Female	43,507	2566	40,941
Ovarian cancer (EO)	ieu‐a‐1125	2017	OCAC+CIMBA	European	28346442	Female	43,751	2810	40,941
Ovarian cancer (CCO)	ieu‐a‐1124	2017	OCAC+CIMBA	European	28346442	Female	42,307	1366	40,941
Cervical cancer	ukb‐b‐8777	2018	UKB	European	NA	Female	462,933	1889	461,044
Oral cavity and pharyngeal cancer	ieu‐b‐89	2016	INHANCE	European	27749845	Female and male	5425	2497	2928
Esophageal cancer	finn‐b‐C3_OESOPHAGUS	2021	FB	European	NA	Female and male	218,792	232	218,560
Cancer of liver and intrahepatic bile ducts	finn‐b‐C3_LIVER_INTRAHEPATIC_BILE_DUCTS	2021	FB	European	NA	Female and male	218,792	304	218,488
Pancreatic cancer	ieu‐a‐822	2009	PanScan1	European	19648918	Female and male	3835	1896	1939
Cancer of urinary organs	finn‐b‐C3_URINARY_TRACT	2021	FB	European	NA	Female and male	218,792	2168	216,624
Prostate cancer	ieu‐b‐85	2018	PRACTICAL	European	29892016	Male	140,254	79,148	61,106
Melanoma	ukb‐a‐58	2017	UKB	European	NA	Female and male	337,159	2677	334,482
Bladder cancer	ukb‐b‐8193	2018	UKB	European	NA	Female and male	462,933	1101	461,832
Glioma	ieu‐a‐1013	2013	GliomaScan	European	22,886,559	Female and male	6811	1856	4955
Thyroid cancer	ieu‐a‐1082	2013	NA	European	23894154	Female and male	1080	649	431
Colon cancer	ukb‐d‐C3_COLON	2018	UKB	European	NA	Female and male	361,194	2437	358,757
Triglycerides	ebi‐a‐GCST002216	2013	GLGC	European	24097068	Female and male	94,595	NA	NA
Cholesterol, total	ebi‐a‐GCST002221	2013	GLGC	European	24097068	Female and male	94,595	NA	NA
LDL cholesterol	ebi‐a‐GCST002222	2013	GLGC	European	24097068	Female and male	94,595	NA	NA
HDL cholesterol	ebi‐a‐GCST002223	2013	GLGC	European	24097068	Female and male	94,595	NA	NA
Type 2 diabetes	ebi‐a‐GCST006867	2018	DIAGRAM+GERA+UKB	European	30054458	Female and male	655,666	61,714	1178
Body mass index	ieu‐a‐835	2015	GIANT	European	25673413	Female and male	322,154	NA	NA
Year of schooling	ieu‐a‐1001	2016	SSGAC	European	27225129	Female and male	293,723	NA	NA
Cheese intake	ukb‐b‐1489	2018	MRC‐IEU	European	NA	Female and male	451,486	NA	NA
Alcoholic drinks intake	ukb‐b‐13,978	2018	MRC‐IEU	European	NA	Female and male	34,317	NA	NA
Chocolate‐covered raisin intake	ukb‐b‐1160	2018	MRC‐IEU	European	NA	Female and male	64,949	NA	NA
Chocolate‐covered biscuits intake	ukb‐b‐5068	2018	MRC‐IEU	European	NA	Female and male	64,949	NA	NA
Tea intake	ukb‐b‐6066	2018	MRC‐IEU	European	NA	Female and male	447,485	NA	NA
Ice‐cream intake	ukb‐b‐17,189	2018	MRC‐IEU	European	NA	Female and male	64,949	NA	NA
Coffee intake	ukb‐b‐5237	2018	MRC‐IEU	European	NA	Female and male	428,860	NA	NA
Cereal intake	ukb‐b‐15,926	2018	MRC‐IEU	European	NA	Female and male	441,640	NA	NA
Oatcakes intake	ukb‐b‐6298	2018	MRC‐IEU	European	NA	Female and male	64,949	NA	NA
Fresh fruit intake	ukb‐b‐3881	2018	MRC‐IEU	European	NA	Female and male	446,462	NA	NA
Fried potatoes intake	ukb‐b‐12,836	2018	MRC‐IEU	European	NA	Female and male	64,949	NA	NA
Drinking water intake	ukb‐b‐3975	2018	MRC‐IEU	European	NA	Female and male	64,949	NA	NA
Sweets intake	ukb‐b‐10,217	2018	MRC‐IEU	European	NA	Female and male	64,949	NA	NA
Muesli intake	ukb‐b‐11,004	2018	MRC‐IEU	European	NA	Female and male	64,949	NA	NA
Fizzy drink intake	ukb‐b‐2832	2018	MRC‐IEU	European	NA	Female and male	64,949	NA	NA
Savory biscuits intake	ukb‐b‐1503	2018	MRC‐IEU	European	NA	Female and male	64,949	NA	NA

Abbreviations: BCAC, Breast Cancer Association Consortium; CCO, clear cell ovarian cancer; CIMBA, Consortium of Investigators of Modifiers of BRCA1/2; DIAGRAM, Diabetes Genetics Replication and Meta‐analysis Consortium; DRIVE, Discovery, Biology and Risk of Inherited Variants in Breast Cancer Consortium; E2C2, the Epidemiology of Endometrial Cancer Consortium; ECAC, Endometrial Cancer Association Consortium; EE, endometrioid endometrial cancer; EO, endometrioid ovarian cancer; ER–, estrogen receptor negative; ER+, estrogen receptor positive; FB, Finnish Biobank; GERA, Genetic Epidemiology Research on Aging; GIANT, Genetic Investigation of Anthropometric Traits Consortium; HDL, high‐density lipoprotein; HGS, high‐grade serous; INHANCE, International Head and Neck Cancer Epidemiology Consortium; LDL, low‐density lipoprotein; LGS+LMPS, low‐grade and low malignant potential serous; MO, mucinous ovarian cancer; MRC‐IEU, MRC Integrative Epidemiology Unit; NEE, non‐endometrioid endometrial cancer; OCAC, Ovarian Cancer Association Consortium; PanScan1, Pancreatic Cancer Cohort Consortium; PRACTICAL, Prostate Cancer Association Group to Investigate Cancer‐Associated Alterations in the Genome Consortium; GLGC, Global Lipid Genetics Consortium; SSGAC, Social Science Genetic Association Consortium; UKB, UK Biobank.

^a^ Overview of genetic data used in the Mendelian randomization analyses. The study population were all European. For case–control studies, the number of cases and controls was reported, and for all studies, the sample size was reported.

SNPs that met the following criteria were selected for MR analysis in the summary data of this GWAS: *p* < 5 × 10^−8^, independent of each other within 5000G, absence of linkage disequilibrium (*r* ^*2*^ ≤ 0.005, *p* < 0.05), and no moderate allele frequency (Table S1).

### 2.2 Instrumental variables for cancers

The IVs associated with 15 cancers (ovarian, endometrial, breast, cervical, prostate, pancreatic, oral and pharyngeal, esophageal, liver and intrahepatic bile ducts, urinary organs, colon, bladder, thyroid, glioma, and melanoma), 6 metabolic factors (triglycerides [TC], total cholesterol, high‐density lipoprotein [HDL] cholesterol level, low‐density lipoprotein [LDL] cholesterol level, body mass index [BMI], and type 2 diabetes [T2D]), 11 dietary factors (intake of fizzy drink, coffee, tea, cereal, fresh fruit, chocolate‐covered raisin, chocolate‐covered biscuits, ice‐cream, fried potatoes, sweets, savory biscuits) and year of schooling were derived from published large GWAS. The detailed information of these studies is shown in Table 1.

### 2.3 Weak instrument bias analyses

Weak instrument bias refers to bias produced by genetic variants with low power to explain exposure owing to insufficient sample size. According to the literature, the *F*‐statistic, equal to (*n*–2)*R* ^2^/(1–*R* ^2^), can be used to evaluate the effect of weak instrumental variables.^28^ *R* ^2^ was the degree of sedentary behaviors explained by the SNP, and was calculated using the formula *R* ^2^ = 2 × (1 – EAF) × EAF×*β* ^2^/(se^2^ × *n*) (EAF refers to effect allele frequency, *n* was the sample size of the GWAS). Weak instrument bias is considered when *F* > 10. In addition, variation between individual genetic variant (*I* ^2^_GX_) was calculated to assess potential weak instrument bias in the MR‐Egger regression analysis. Low risk of measurement bias was considered at *I* ^2^_GX_ > 95%.^29^

### 2.4 Univariable Mendelian randomization analysis

The causal relationship between individual SNPs and cancers was estimated by *β*‐value (Table S3). Then an inverse‐variance‐weighted (IVW) (fixed‐effects) method was used to summarize the *β*‐values of individual SNPs to obtain the total effect of LSB on 15 site‐specific cancers.

**FIGURE 1:**
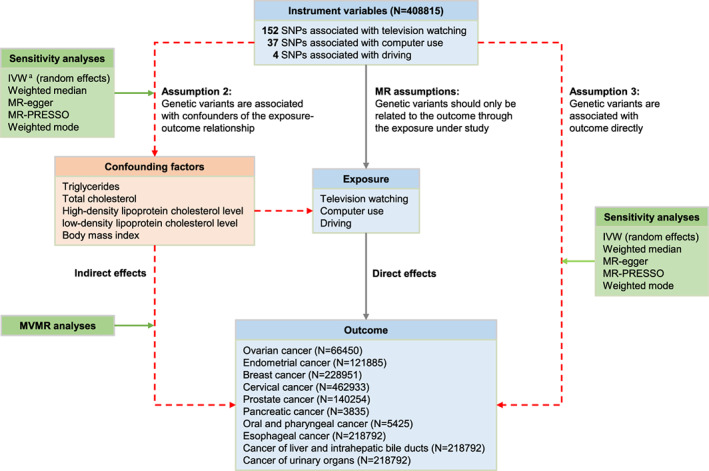
Overview of Mendelian randomization analyses. Solid gray lines represent the direct effect of instrument variables on outcomes through the exposure under study (television watching, computer use, and driving). Dashed red lines represent indirect effects of instrument variables on outcomes that have potential violation of Mendelian randomization assumptions. IVW, inverse‐variance‐weighted method.

**FIGURE 2:**
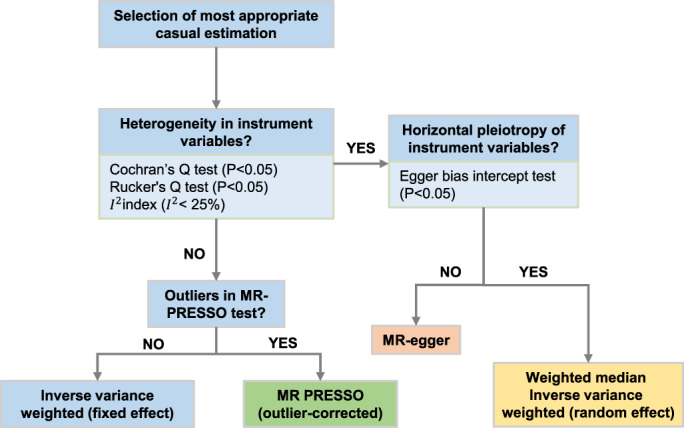
Selection of the most appropriate causal estimation. It was reported that different Mendelian randomization sensitivity analyses made different assumptions about horizontal pleiotropy, heterogeneity, and error in the instrument‐exposure associations. Thus, the most appropriate Mendelian randomization sensitivity analysis should be selected for estimating the associations according to differing horizontal pleiotropy and heterogeneity.

Further sensitivity analyses, including IVW (random‐effects), weighted‐median, MR‐Egger, MR‐PRESSO, and weighted‐mode, were performed to account for presence of horizontal pleiotropy, heterogeneity, potential violations of the MR assumptions, and invalid instrument‐exposure associations (Figure 1).^30^, ^31^, ^32^ First, Cochran's Q and Rucker's Q statistic values were calculated to test for the presence of heterogeneity in the IVW (fixed‐effects) and MR‐Egger, respectively. Heterogeneity was considered at *p* < 0.05. *I* ^*2*^ index, equal to ($\frac{Q–\mathrm{df}}{Q}\times 100\%$) (*Q* represents the quantitative value of Cochran's *Q* test, *df* represents degree of freedom) was also calculated, and heterogeneity was considered as significant if *I* ^*2*^ > 25%.^33^ Once heterogeneity exists, either SNPs with very small *p*‐value for outcomes should be excluded, or random‐effects models and weight‐median are more appropriate for estimating MR effect (Figure 2).^30^, ^34^ Second, the Egger bias intercept test was used to detect the presence of horizontal pleiotropy.^31^ Significant difference between MR‐Egger intercept and 0 was evidence of horizontal pleiotropy. MR‐Egger could appropriately estimate the causal effect in this situation (Figure 2). The MR‐PRESSO is also a commonly used R package for pleiotropy. It can test for horizontal pleiotropy, remove pleiotropic outliers, and test for estimation differences before and after the outliers are removed.^35^

Horizontal pleiotropy, outliers, and heterogeneity were visually analyzed by using scatterplots, leave‐one‐out plots, and funnel plots.^36^ MR‐Steiger analysis was performed to validate the direction of causality between exposure and outcomes.^37^

To determine if the results were robust to the *p*‐value threshold, we added genetic variants for sedentary behavior with higher *p*‐value (<1 × 10^−7^, < 1 × 10^−6^) and repeated the MR analysis.

### 2.5 Multivariable Mendelian randomization analysis

GWAS studies of LSB have revealed some degree of genetic association between sedentary behaviors and other traits including triglycerides, total cholesterol, HDL, LDL, BMI, T2D, and educational attainment.^27^ Other studies revealed the association between sedentary behaviors and dietary habits.^38^, ^39^ The effects of LSB on these traits were estimated using UVMR analyses and the significantly associated traits were included in MVMR analysis.

To investigate whether the effect of LSB on cancers may be mitigated by its effect on other traits and prevent potential MR assumption violations, MVMR was used to estimate the direct effect of multiple exposures on different outcomes (Figure 1).^40^ The SNPs used for MVMR analysis were linked to both LSB and a second exposure; exclusion criteria were consistent with UVMR analysis.

### 2.6 Statistical analysis

Primary univariate and multivariate analyses of the relationship between LSB and cancers were conducted with a two‐sided significance threshold of *p* < 0.05. Correction for *p*‐value was made using the Bonferroni method in the secondary analysis of LSB on metabolic factors (*p* < 0.05/9 = 0.006). Associations were considered suggestive at 0.006 < *p* < 0.05. Causal associations were estimated using the odds ratio (OR). All analyses were finished using the R package TwoSampleMR (version 0.5.6). Data analyses were conducted between January 2021 and June 2022.

## 3 RESULTS

### 3.1 Instrument variables

A total of 194 IVs were included in this study, of which 4 were related to driving, 37 to computer use, and 152 to television watching. Heritability referred to the proportion of genetic variance of all SNPs in the total variance and it was used to assess the degree to which SNPs affected the trait. Heritability was estimated from the literature to be 16.1%, 9.3%, and 4.4% for television watching, computer use, and driving, respectively.^27^ In this study, the *F*‐value of IVs related to television watching, computer use, and driving ranged from 23.94 to 144.19, 24.07 to 79.56, and 24.00 to 45.01, respectively. None of them were considered to have weak instrument bias (*F* > 10) (Table S1). As calculated in the original literature, *I* ^2^_GX_ for television watching, computer use, and driving were 0.98, 0.98, and 0, respectively, indicating a low chance of weak instrument bias except for driving.^27^ Statistical power of these IVs was calculated using mRnd power calculator.^41^ Statistical power of television watching was 100%. However, statistical power of computer use and driving remained scarce (<80%) because of the low heritability. (Table S2).

### 3.2 Causal effects of television watching on 15 site‐specific cancers

**FIGURE 3:**
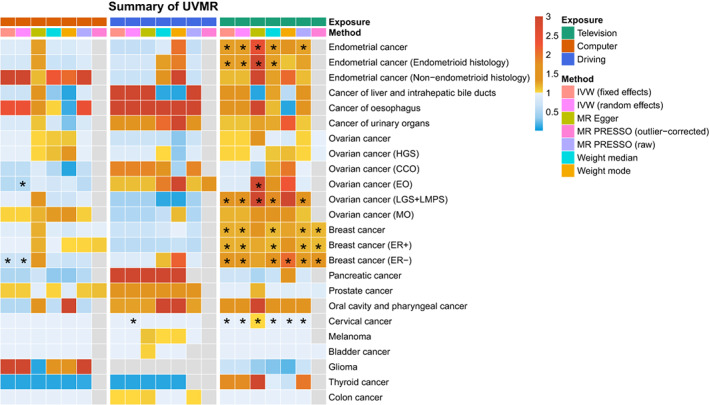
Summary of the univariable Mendelian randomization results for leisure sedentary behaviors and 10 site‐specific cancers. Total effect sizes for associations between leisure sedentary behaviors and 10 site‐specific cancers were estimated using seven different methods. Asterisks indicate that the association is nominally significant (*p* < 0.05). Color is scaled based on the Mendelian randomization odds ratio estimates, and associations for which no instrument was available are presented as white tiles.

Complete results of UVMR of the association between 3 sedentary behaviors and 15 site‐specific cancers are summarized in Figure 3. It was obvious that significant positive results were obtained mainly for associations between television watching and female cancers.

Using an IVW fixed‐effects approach, a one‐standard deviation (1‐SD) increase in watching television increased endometrial cancer risk by nearly 30% (OR, 1.28; 95% CI, 1.06–1.55; *p* = 0.010). It occurred in endometrioid subtype (OR, 1.28; 95% CI, 1.02–1.60; *p* = 0.031) rather than non‐endometrioid subtype (OR, 1.15; 95% CI, 0.72–1.86; *p* = 0.56) (Table S3, Figure S1). There was no evidence of horizontal pleiotropy (*p*‐intercept >0.05). However, there was significant heterogeneity except for non‐endometrioid subtype (Table S4, Figures S2–S13). After controlling the heterogeneity by using a weighted‐median and IVW random‐effects approach, the causal effect of television watching on overall endometrial cancer and endometrioid subtype remained significant.

Similarly, television watching was significantly associated with breast cancer (OR, 1.16; 95% CI, 1.04–1.30; *p* = 0.007) in the IVW fixed‐effects analyses. The association appeared to be stronger for the estrogen receptor negative (ER–) subtype (OR, 1.55; 95% CI, 1.26–1.89; *p* = 2.23 × 10^−5^) than for estrogen receptor positive (ER+) subtype (OR, 1.17; 95% CI, 1.03–1.33; *p* = 0.015) (Table S3, Figure S1). In the following sensitivity analyses, we used the outlier (MR‐PRESSO) method to test and correct for 3, 3, and 2 horizontal pleiotropic outliers for overall, ER+, and ER– breast cancer, respectively. After excluding these outliers, the causal estimates showed no significant differences (Tables S5, S6). Given that the average pleiotropic effect was minor and the intercept from the MR‐Egger regression was not statistically significant, the influence of pleiotropy may have been minimal (Table S4, Figures S14–S25). In addition, although heterogeneity was significant, the results of control analysis with breast cancer and two subtypes as the outcome remained significant.

The results for low‐grade and low malignant potential serous ovarian cancer demonstrated an approximately 49% increase in risk, 1‐SD increase in television watching (OR, 1.49; 95% CI, 1.07–2.08; *p* = 0.018) without significant pleiotropy and heterogeneity (Tables S3–S6, Figures S26–S49). This association was also supported by MR‐Egger and weighted‐median analyses. However, overall and other subtype analyses of ovarian cancer yielded statistically significant results in all UVMR sensitivity analyses (Figure S1).

Although the causal effect between television watching and cervical cancer was significant in all sensitivity analyses, it was extremely weak (OR, 1.003; 95% CI, 1.001–1.005; *p =* 4.7 × 10^−4^) (Tables S3 and S4, Figures S50–S53). This association might be explained by the lack of sufficient adjustment and residual confusion, although it seemed to be a new finding.

Aside from the above results, television watching made no significant difference to the other 11 cancers. There was no evidence of horizontal pleiotropy or heterogeneity except for prostate cancer. But even when we used control analyses to correct the heterogeneity, no significant results were obtained (Tables S3–S6, Figures S54–S99).

### 3.3 Causal effects of computer use and driving on 15 site‐specific cancers

There were no significant results in any of the UVMR analyses of relationship between driving and the 15 site‐specific cancers. UVMR analysis of computer use also failed to obtain any significant results except for non‐endometrioid endometrial cancer (OR, 2.99; 95% CI, 1.19–7.49; *p* = 0.019) and cervical cancer (OR, 1.002; 95% CI, 0.99–1.005; *p* = 0.208). Both of the latter findings, however, were supported by only one MR method, whereas all other methods consistently showed null results for computer use and endometrial and cervical cancer (Tables S3–S6). Thus, we considered them as false positive results.

A lowered *p*‐value threshold for IVs was used and the UVMR analyses were repeated, considering the small number of driving and computer use variants used in the MR analyses. The genetic association was independent of *p*‐value thresholds, as the results remained non‐significant with the change of *p*‐value thresholds (Tables S3–S6). Therefore, caution should be exercised in considering driving and computer use as causal risk factors for cancers.

### 3.4 Multiple variables Mendelian randomization

We investigated the causal association between sedentary behaviors and metabolic factors, educational attainment and dietary habits through UVMR. TV viewing was found to be causally associated with BMI (OR, 1.29; 95% CI, 1.19–1.40; *p* < 0.001), TC (OR, 1.19; 95% CI, 1.07–1.31; *p* < 0.001), LDL (OR, 1.13; 95% CI, 1.00–1.27; *p* = 0.04), and T2D (OR, 1.87; 95% CI, 1.61–2.19; *p* < 0.001), while negatively associated with HDL (OR, 0.82; 95% CI, 0.75–0.90; *p* < 0.001) and years of schooling (OR, 0.60; 95% CI, 0.56–0.64; *p* < 0.001). For dietary habits, TV viewing was a risk factor of most unhealthy dietary habits (intake of chocolate‐covered raisin [OR, 1.02; 95% CI, 1.00–1.04; *p* = 0.02], fizzy drink [OR, 1.07; 95% CI, 1.02–1.12; *p* = 0.006], fried potatoes [OR, 1.15; 95% CI, 1.05–1.26; *p* = 0.003], and sweets [OR, 1.06; 95% CI, 1.00–1.11; *p* = 0.03]), but was negatively associated with other dietary habits (intake of cereal [OR, 0.85; 95% CI, 0.82–0.89; *p* < 0.001], fresh fruit [OR, 0.91; 95% CI, 0.88–0.93; *p* < 0.001], and coffee [OR, 0.91; 95% CI, 0.88–0.94; *p* < 0.001]). Computer use was showed to be positively associated with years of schooling (OR, 1.78; 95% CI, 1.48–2.14; *p* < 0.001) and intake of cereal (OR, 1.15; 95% CI, 1.02–1.30; *p* = 0.02). However, there were no significant associations between computer use and all metabolic factors as well as all unhealthy dietary habits. As for driving, it was only showed to be associated with year of schooling (OR, 0.59; 95% CI, 0.37–0.95; *p* = 0.03) (Tables S7–S9). Interestingly, educational attainment was inversely associated with TV viewing, while positively associated with computer use. MVMR analysis was performed to ensure that the causal association between television watching and endometrial, breast, and ovarian cancer came from the direct influence of television watching rather than significant confounding variables.

**FIGURE 4:**
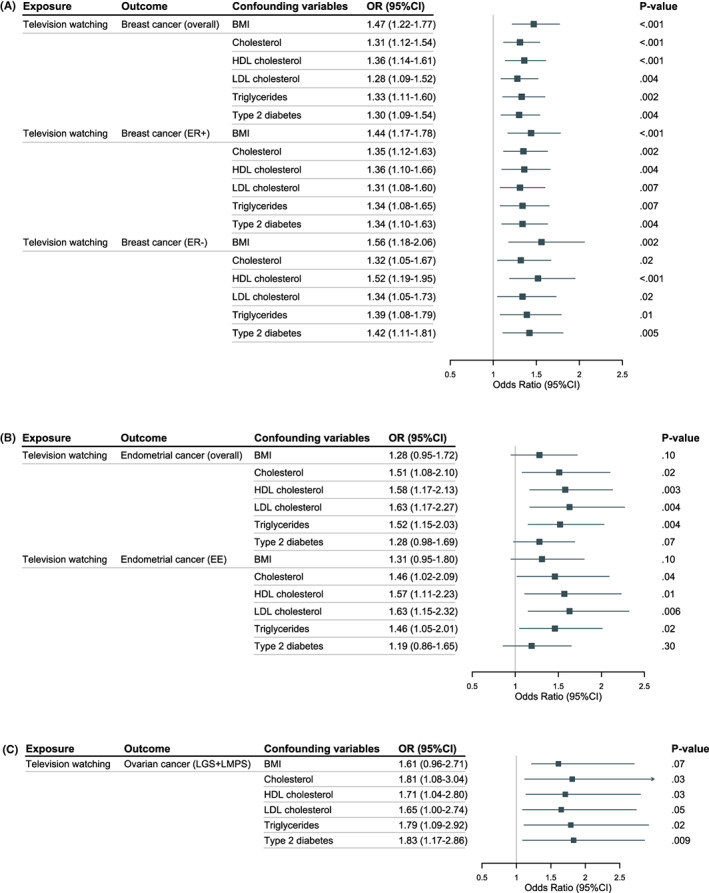
Forest plot of additional multivariate Mendelian randomization analyses of the relationship between sedentary behaviors and three female cancers. Direct effect sizes for associations between leisure sedentary behaviors and (A) breast cancer, (B) endometrial cancer, and (C) ovarian cancer were estimated by multivariable randomization analysis. Confounding factors included body mass index, triglycerides, cholesterol, high‐density lipoprotein cholesterol, low‐density lipoprotein cholesterol in serum, and type 2 diabetes. ER+, estrogen receptor positive breast cancer; ER–, estrogen receptor negative breast cancer; EE, endometrioid endometrial cancer; LGS + LMPS, low‐grade and low malignant potential serous ovarian cancer.

For endometrial cancer, adjustment of BMI, T2D, and educational attainment made the previously causal association non‐significant, indicating that BMI, T2D, and educational attainment were mediators in this association. Adjustment of BMI also indicated it as the mediators in the association between television watching and low‐grade serous ovarian cancer. Besides, only educational attainment changed the association between television watching and breast cancer, indicating it as a mediator (Figure 4, Table S9).

## 4 DISCUSSION

Our study explored the causal relationship between LSBs and 15 site‐specific cancers, using MR analyses. After complete sensitivity analyses, the associations were converged on three common female cancers – endometrial, breast, and ovarian cancer. Furthermore, the associations varied among subtypes of the three female cancers. However, computer use and driving were not seen as risk factors for the selected cancers. Further MVMR analyses indicated the direct effect of television watching on the three female cancers. Given the high stability of the positive results, we considered television watching as a risk factor for endometrial cancer, breast cancer, and ovarian cancer. Whether computer use and driving were risk factors was not conclusive.

We found that television watching increased the risk of endometrial cancer, especially in endometroid subtype. This is in accordance with the previous large meta‐analyses that found a consistent association between television watching and incidence of endometrial cancer.^15^, ^16^, ^42^ Having said that, the novelty of our study lies in that we obtained causal associations between LSB and endometrial cancer instead of observational associations. It should also be noted that another meta‐analysis previously reported a non‐significant result [risk ratio (RR), 1.05; 95% CI, 0.51–2.15].^14^ However, only one cohort study, with a low quality rating of evidence, was included in analysis. The incomplete literature search and different definitions of television watching might be the reasons for this discrepancy.

We indicated television watching as a risk factor for breast cancer. Contrary to our findings, no significant causal association was shown in two other meta‐analyses.^15^, ^43^ However, the definition and measurement methods of sedentary behaviors in these studies were highly heterogeneous, which might account for this discrepancy. Other studies have established the causal association between LSB and breast cancer risk.^16^, ^42^, ^44^, ^45^, ^46^ But the association between LSB and breast cancer incidence by ER status was still unclear.^46^, ^47^, ^48^ Even if LSB increased the risk of ER–breast cancer more obviously in a study similar to ours,^48^ that study only included a small number of cases for analysis owing to the lack of subtype information in all the breast cancer cases, limiting its validity. With more rigorous and complete analyses, our study effectively fills the gaps in existing research.

We also demonstrated causal associations between LSB and ovarian cancer in line with a large meta‐analysis.^49^ But it must be pointed out that the observed estimates of the association in this study were inflated because women who volunteered as controls in this study were generally healthier. Thus observed evidence on LSB and ovarian cancer in our study is an important finding, particularly as no meaningful and reliable association has been found between sedentary behaviors and ovarian cancer risk in several large studies.^8^, ^49^, ^50^ For other 12 cancers, only colon cancer had observational associations with LSB in previous studies,^14^, ^15^ but we could not identify any meaningful causal associations by rigorous MR analyses despite this.

Another key point to note is that causal effects of driving and computer use on the selected cancers were either non‐significant or unestablished in several sensitivity analyses. For driving trait, inadequate SNPs and low statistical power might be the reasons. For computer use, the follow‐up pre‐UVMR and MVMR analysis gave the possible explanation. Pre‐UVMR analysis revealed high genetic correlations between sedentary behaviors and educational attainment, which were negative for TV viewing and driving, and positive for computer use. In addition, causal associations were found between TV viewing and cardio‐metabolic factors as well as unhealthy dietary habits, which were insignificant for computer use. It seemed that people spending more time in using computer had higher educational attainment and healthier dietary habits. And education had been presumed to causally influence health because it generally confers greater access to salubrious resources such as economic security, healthy lifestyles, social ties, fulfilling jobs, a sense of personal control, and learned effectiveness.^51^ That said, volunteering bias in the questionnaires data was one possible explanation for the difference. We therefore take caution in determining driving and computer use as causal risk factors for cancer.

The multivariable MR analyses showed an effect of television watching on female cancer independent of most cardio‐metabolic factors and dietary habits. It also indicated vertical pleiotropy of BMI, T2D, intake of fizzy drink and biscuits, as the direct effects of TV viewing on cancers were attenuated compared with the total effects. This provides genetic insights in how sedentary behaviors are associated with cancers. LSB could cause increased snack intake and decreased energy expenditure, accompanied by weight gain and obesity, which could increase risk of cancer. Obesity facilitates carcinogenesis through a number of pathways, including insulin resistance, perturbations in the insulin‐like growth factor axis, and low‐grade systemic inflammation.^52^, ^53^, ^54^, ^55^, ^56^ In postmenopausal women, adipose tissue is the main site for aromatization of androgen precursors to produce estrogen, which would increase the risk for endometrial cancer.^57^ It was also shown that reducing physical activity can increase serum levels of estradiol and decreased sex hormone binding globulin, thereby affecting the development of many female cancers.^14^, ^43^, ^58^ Other mechanisms include influence on BRCA1 gene status, decreased vitamin D levels, imbalance of inflammatory factors, and altered telomere length.^18^, ^59^, ^60^, ^61^, ^62^, ^63^

The multivariable MR in which we corrected for education indicated pleiotropy due to education. Pre‐UVMR analysis in our study revealed correlations between sedentary behaviors and educational attainment in line with previous study. Besides, we have also verified the inverse association between educational attainment and sedentary behaviors in line with a previous study. This study also found that higher educational attainment levels were positively with vigorous physical activity levels and alcohol consumption.^64^ In summary, traits like education, sedentary behaviors, and dietary habits are correlated and it is therefore difficult to disentangle their complex interrelationships. Both causal directions taken together would point to education having a complex dual mediating and confounding role in the association between television watching with cancer risk.

The strengths of our study are, initially, that it is the first to explore and find a causal association between sedentary behavior and three common female cancers using MR and that different associations were found within different subtypes with reliable evidence. The results suggest a potential role of LSB in cancer prevention. We may need to promote lifestyle changes that reduce sedentary time for the general public. Second, the instrumental variables used in this study were derived from a large GWAS with significant statistical power. In addition, this study carried out sensitivity analyses using several different methods to test the heterogeneity and pleiotropy of IVs and correct the directionality of causal associations. The test results confirmed one another, making the results more reliable. Finally, MVMR was performed on the positive results obtained from UVMR, which revealed the direct effect of LSB on the corresponding cancers.

The first limitation of this study is that the population included in this study was European, and it is not clear whether the conclusions of the study can be extended to other ethnic groups. Second, SNPs obtained by statistical methods rather than biological methods inevitably have pleiotropy, for which we used multiple sensitivity analyses to minimize the impact of pleiotropy. We also performed MVMR to correct the effects of confounders. However, this does not rule out the effects of other unknown, potentially confounding variables. In addition, the acquisition of leisure sedentary time was based on subjective measurements rather than with measurement tools; although this avoided measurement bias, it was pointed out that subjective and objective measurement standards are non‐uniform.^65^ LSB owing to occupational factors is also an important form of LSB, but the GWAS of IVs do not include it because of the lack of relevant data, which may have resulted in underestimation of LSB. The use of more precise means to determine sedentary time should therefore be considered in the future. An additional limitation of our study is that the GWAS studies for LSB involved both male and female, whereas female cancer was assessed only in women. Therefore, our results might be biased if the effects of the genetic variants are different between two sexes. However, the jury is still out on whether genetic variants of LSB had sex‐specific effects. Under this situation, applying sex‐combined IVs makes an implicit hypothesis that no effect differences exist between males and females, which, however, is not necessarily satisfied. Nevertheless, the use of sex‐combined IVs in MR analysis is not without advantages if such assumption can be well‐established. In this case, one of the greatest benefits is that more IVs would be exploited on account of a larger sample size for the exposure GWAS, which can potentially improve statistical power due to more phenotypic variances explained. Another study indicated that MR analysis might still provide evidence on whether a causal association exists but not necessarily on the precise magnitude of the causal effect when sex‐combined IVs were used.^66^ Additionally, we carefully examined heterogeneity in instruments and performed rigorous sensitivity analyses to ensure the robustness of the results according to the suggestions provided by a recent study.^67^ Finally, in the present study, we failed to perform stratified analysis of the study subjects by age, gender, and other demographic characteristics.

## AUTHOR CONTRIBUTIONS

**Jinwei Chen:** Conceptualization (lead); data curation (lead); formal analysis (lead); visualization (lead); writing – original draft (lead). **Kai‐Bin Yang:** Conceptualization (lead); formal analysis (lead); visualization (lead); writing – original draft (lead). **Youyu Qiu:** Conceptualization (supporting); formal analysis (lead); visualization (equal); writing – original draft (lead); writing – review and editing (equal). **Weijie Lai:** Data curation (lead); visualization (equal). **Sifan Qi:** Data curation (lead); visualization (supporting). **Gaoyuan Wang:** Data curation (equal); visualization (supporting). **Lin Chen:** Data curation (equal); writing – original draft (equal). **Kunpeng Li:** Data curation (supporting); writing – original draft (equal). **Dan Zhou:** Data curation (supporting); writing – original draft (supporting). **Qing Liu:** Formal analysis (equal); visualization (supporting); writing – original draft (lead). **Ling‐Long Tang:** Formal analysis (supporting); visualization (supporting); writing – original draft (equal). **Xu Liu:** Formal analysis (supporting); visualization (supporting); writing – review and editing (equal). **Xiao‐Jing Du:** Conceptualization (lead); project administration (lead); writing – review and editing (equal). **Rui Guo:** Conceptualization (supporting); supervision (lead); writing – review and editing (equal). **Jun Ma:** Conceptualization (lead); funding acquisition (lead); project administration (lead); supervision (lead); writing – review and editing (lead).

## FUNDING INFORMATION

This study was supported by grants from the National Natural Science Foundation of China (81930072, 82172870), the Key‐Area Research and Development Program of Guangdong Province (2019B020230002), and the Overseas Expertise Introduction Project for Discipline Innovation (111 Project, B14035).

## CONFLICT OF INTEREST STATEMENT

No potential conflict of interest was reported by the authors.

## CONSENT FOR PUBLICATION

Not applicable.

## ETHICS APPROVAL AND CONSENT TO PARTICIPATE

The study strictly followed the Declaration of Helsinki. The data were anonymized and publicly available, thus institutional ethics committee approval and consent from the participants were not required.

## Supporting information


Figures S1–S99.

Tables S1–S9.

## Data Availability

The original data supporting the findings of this study are available in the MR BASE database at https://www.mrbase.org/ or included in the supplementary materials. Data directly related to the results and code for data cleaning and analysis in this study are available from the corresponding authors upon reasonable request.
