# Supplementary material for: Genetic associations of leisure sedentary behaviors and the risk of 15 site‐specific cancers: A Mendelian randomization study
Source: Cancer Med. 2023 May 6;12(12):13623–36. doi: 10.1002/cam4.5974 (PMC10315754; doi:10.1002/cam4.5974)
Supplement: Supplementary file 1 — Figures S1–S99. [file CAM4-12-13623-s002.docx]

Online-only Supplements

Genetic Associations of Leisure Sedentary Behaviors and the Risk of 15 site-specific cancers, A Mendelian Randomization Study

Jinwei Chen, M.D.,^*^ Kaibin Yang, M.D.,^*^ Youyu Qiu, M.D.,^*^ Weijie Lai, M.D., Sifan Qi, M.D., Gaoyuan Wang, M.D., Lin Chen, M.D., Kunpeng Li, M.D., Dan Zhou, NP, Qing Liu, M.D., Linglong Tang, M.D., Xu Liu, M.D., Xiaojing Du, M.D.,^†^ Rui Guo, M.D.,^†^ Jun Ma, M.D. ^†^

# Supplementary Materials Checklist

**Supplementary Tables**

**eTable 1.** Significant instrument variables of traits of leisure sedentary behaviors and weak instruments analyses

**eTable 2.** Statistical power of intrument variables

**eTable 3.** Results from univariable Mendelian randomization analyses of relationships between leisure sedentary behaviors and 15 site-specific cancers

**eTable 4.** Heterogeneity, pleiotropy, and Steiger directionality test in the examined associations between leisure sedentary behaviors and 15 site-specific cancers

**eTable 5.** Significant instrument variable outliers of leisure sedentary behaviors that were excluded in the MR-PRESSO analyses

**eTable 6.** Results from MR-PRESSO analyses and outlier-corrected analyses

**eTable 7.** Results from pre-univariable Mendelian randomization analysis of the association between traits of leisure sedentary behaviors and the secondary exposure

**eTable 8.** Heterogeneity, pleiotropy, and Steiger directionality test in the associations between leisure sedentary behaviors and the confounding variables

**eTable 9.** Results of multivariable Mendelian randomization of relationships between television watching, covariate risk factors and three female cancers

**Supplementary Figures**

**eFigure 1.** Results of the univariable Mendelian randomization analyses of the association between television watching and endometrial cancer

**eFigure 2.** Results of the univariable Mendelian randomization analyses of the association between television watching and breast cancer

**eFigure 3.** Results of the univariable Mendelian randomization analyses of the association between television watching and ovarian cancer

**Summary illustration of eFigures 4–79**

**eFigures of endometrial cancer (overall)**

**eFigure 4.** Funnel plots of leisure sedentary behaviors and endometrial cancer (overall).

**eFigure 5.** Scatter plots of leisure sedentary behaviors and endometrial cancer (overall).

**eFigure 6.** Leave-one-out plots of leisure sedentary behaviors and endometrial cancer (overall).

**eFigure 7.** Forest plots of single-SNP analysis of leisure sedentary behaviors and endometrial cancer (overall).

**eFigures of endometrial cancer (endometrioid histology)**

**eFigure 8.** Funnel plots of leisure sedentary behaviors and endometrioid endometrial cancer.

**eFigure 9.** Scatter plots of leisure sedentary behaviors and endometrioid endometrial cancer.

**eFigure 10.** Leave-one-out plots of leisure sedentary behaviors and endometrioid endometrial cancer.

**eFigure 11.** Forest plots of single-SNP analysis of leisure sedentary behaviors and endometrioid endometrial cancer.

**eFigures of endometrial cancer (non-endometrioid histology)**

**eFigure 12.** Funnel plots of leisure sedentary behaviors and non-endometrioid endometrial cancer.

**eFigure 13.** Scatter plots of leisure sedentary behaviors and non-endometrioid endometrial cancer.

**eFigure 14.** Leave-one-out plots of leisure sedentary behaviors and non-endometrioid endometrial cancer.

**eFigure 15.** Forest plots of single-SNP analysis of leisure sedentary behaviors and non-endometrioid endometrial cancer.

**eFigures of breast cancer (overall)**

**eFigure 16.** Funnel plots of leisure sedentary behaviors and breast cancer (overall).

**eFigure 17.** Scatter plots of leisure sedentary behaviors and breast cancer (overall).

**eFigure 18.** Leave-one-out plots of leisure sedentary behaviors and breast cancer (overall).

**eFigure 19.** Forest plots of single-SNP analysis of leisure sedentary behaviors and breast cancer (overall).

**eFigures of breast cancer (ER**–**)**

**eFigure 20.** Funnel plots of leisure sedentary behaviors and breast cancer (ER–).

**eFigure 21.** Scatter plots of leisure sedentary behaviors and breast cancer (ER–).

**eFigure 22.** Leave-one-out plots of leisure sedentary behaviors and breast cancer (ER–).

**eFigure 23.** Forest plots of single-SNP analysis of leisure sedentary behaviors and breast cancer (ER–).

**eFigures of breast cancer (ER+)**

**eFigure 24.** Funnel plots of leisure sedentary behaviors and breast cancer (ER+).

**eFigure 25.** Scatter plots of leisure sedentary behaviors and breast cancer (ER+).

**eFigure 26.** Leave-one-out plots of leisure sedentary behaviors and breast cancer (ER+).

**eFigure 27.** Forest plots of single-SNP analysis of leisure sedentary behaviors and breast cancer (ER+).

**eFigures of ovarian cancer (low grade and low malignant potential serous subtype)**

**eFigure 28.** Funnel plots of leisure sedentary behaviors and ovarian cancer (LGS+LMPS).

**eFigure 29.** Scatter plots of leisure sedentary behaviors and ovarian cancer (LGS+LMPS).

**eFigure 30.** Leave-one-out plots of leisure sedentary behaviors and ovarian cancer (LGS+LMPS).

**eFigure 31.** Forest plots of single-SNP analysis of leisure sedentary behaviors and ovarian cancer (LGS+LMPS).

**eFigures of ovarian cancer (overall)**

**eFigure 32.** Funnel plots of leisure sedentary behaviors and ovarian cancer (overall).

**eFigure 33.** Scatter plots of leisure sedentary behaviors and ovarian cancer (overall).

**eFigure 34.** Leave-one-out plots of leisure sedentary behaviors and ovarian cancer (overall).

**eFigure 35.** Forest plots of single-SNP analysis of leisure sedentary behaviors and ovarian cancer (overall).

**eFigures of ovarian cancer (high grade serous subtype)**

**eFigure 36.** Funnel plots of leisure sedentary behaviors and high grade serous ovarian cancer.

**eFigure 37.** Scatter plots of leisure sedentary behaviors and high grade serous ovarian cancer.

**eFigure 38.** Leave-one-out plots of leisure sedentary behaviors and high grade serous ovarian cancer.

**eFigure 39.** Forest plots of single-SNP analysis of leisure sedentary behaviors and high grade serous ovarian cancer.

**eFigures of ovarian cancer (clear cell subtype)**

**eFigure 40.** Funnel plots of leisure sedentary behaviors and clear cell ovarian cancer.

**eFigure 41.** Scatter plots of leisure sedentary behaviors and clear cell ovarian cancer.

**eFigure 42.** Leave-one-out plots of leisure sedentary behaviors and clear cell ovarian cancer.

**eFigure 43.** Forest plots of single-SNP analysis of leisure sedentary behaviors and clear cell ovarian cancer.

**eFigures of ovarian cancer (endometrioid subtype)**

**eFigure 44.** Funnel plots of leisure sedentary behaviors and endometrioid ovarian cancer.

**eFigure 45.** Scatter plots of leisure sedentary behaviors and endometrioid ovarian cancer.

**eFigure 46.** Leave-one-out plots of leisure sedentary behaviors and endometrioid ovarian cancer.

**eFigure 47.** Forest plots of single-SNP analysis of leisure sedentary behaviors and endometrioid ovarian cancer.

**eFigures of ovarian cancer (mucinous subtype)**

**eFigure 48.** Scatter plots of leisure sedentary behaviors and mucinous ovarian cancer.

**eFigure 49.** Funnel plots of leisure sedentary behaviors and mucinous ovarian cancer.

**eFigure 50.** Leave-one-out plots of leisure sedentary behaviors and mucinous ovarian cancer.

**eFigure 51.** Forest plots of single-SNP analysis of leisure sedentary behaviors and mucinous ovarian cancer.

**eFigures of cervical cancer**

**eFigure 52.** Funnel plots of leisure sedentary behaviors and cervical cancer.

**eFigure 53.** Scatter plots of leisure sedentary behaviors and cervical cancer.

**eFigure 54.** Leave-one-out plots of leisure sedentary behaviors and cervical cancer.

**eFigure 55.** Forest plots of single-SNP analysis of leisure sedentary behaviors and cervical cancer.

**eFigures of prostate cancer**

**eFigure 56.** Funnel plots of leisure sedentary behaviors and prostate cancer.

**eFigure 57.** Scatter plots of leisure sedentary behaviors and prostate cancer.

**eFigure 58.** Leave-one-out plots of leisure sedentary behaviors and prostate cancer.

**eFigure 59.** Forest plots of single-SNP analysis of leisure sedentary behaviors and prostate cancer.

**eFigures of esophageal cancer**

**eFigure 60.** Funnel plots of leisure sedentary behaviors and esophageal cancer.

**eFigure 61.** Scatter plots of leisure sedentary behaviors and esophageal cancer.

**eFigure 62.** Leave-one-out plots of leisure sedentary behaviors and esophageal cancer.

**eFigure 63.** Forest plots of single-SNP analysis of leisure sedentary behaviors and esophageal cancer.

**eFigures of liver and intrahepatic bile ducts cancer**

**eFigure 64.** Funnel plots of leisure sedentary behaviors and cancer of liver and intrahepatic bile ducts.

**eFigure 65.** Scatter plots of leisure sedentary behaviors and cancer of liver and intrahepatic bile ducts.

**eFigure 66.** Leave-one-out plots of leisure sedentary behaviors and cancer of liver and intrahepatic bile ducts.

**eFigure 67.** Forest plots of single-SNP analysis of leisure sedentary behaviors and cancer of liver and intrahepatic bile ducts.

**eFigures of liver and pancreatic cancer**

**eFigure 68.** Funnel plots of leisure sedentary behaviors and pancreatic cancer.

**eFigure 69.** Scatter plots of leisure sedentary behaviors and pancreatic cancer.

**eFigure 70.** Leave-one-out plots of leisure sedentary behaviors and pancreatic cancer.

**eFigure 71.** Forest plots of single-SNP analysis of leisure sedentary behaviors and pancreatic cancer.

**eFigures of oral and pharyngeal cancer**

**eFigure 72.** Funnel plots of leisure sedentary behaviors and cancer of oral cavity and pharynx.

**eFigure 73.** Scatter plots of leisure sedentary behaviors and cancer of oral cavity and pharyngeal.

**eFigure 74.** Leave-one-out plots of leisure sedentary behaviors and cancer of oral cavity and pharynx.

**eFigure 75.** Forest plots of single-SNP analysis of leisure sedentary behaviors and cancer of oral cavity and pharynx.

**eFigures of urinary organs cancer**

**eFigure 76.** Scatter plots of leisure sedentary behaviors and cancer of urinary organs.

**eFigure 77.** Funnel plots of leisure sedentary behaviors and cancer of urinary organs.

**eFigure 78.** Leave-one-out plots of leisure sedentary behaviors and cancer of urinary organs.

**eFigure 79.** Forest plots of single-SNP analysis of leisure sedentary behaviors and cancer of urinary organs.

**eFigures of colon cancer**

**eFigure 80.** Scatter plots of leisure sedentary behaviors and colon cancer.

**eFigure 81.** Funnel plots of leisure sedentary behaviors and colon cancer.

**eFigure 82.** Leave-one-out plots of leisure sedentary behaviors and colon cancer.

**eFigure 83.** Forest plots of single-SNP analysis of leisure sedentary behaviors and colon cancer.

**eFigures of thyroid cancer**

**eFigure 84.** Scatter plots of leisure sedentary behaviors and thyroid cancer.

**eFigure 85.** Funnel plots of leisure sedentary behaviors and thyroid cancer.

**eFigure 86.** Leave-one-out plots of leisure sedentary behaviors and thyroid cancer.

**eFigure 87.** Forest plots of single-SNP analysis of leisure sedentary behaviors and thyroid cancer.

**eFigures of melanoma**

**eFigure 88.** Scatter plots of leisure sedentary behaviors and melanoma.

**eFigure 89.** Funnel plots of leisure sedentary behaviors and melanoma.

**eFigure 90.** Leave-one-out plots of leisure sedentary behaviors and melanoma.

**eFigure 91.** Forest plots of single-SNP analysis of leisure sedentary behaviors and melanoma.

**eFigures of bladder cancer**

**eFigure 92.** Scatter plots of leisure sedentary behaviors and bladder cancer.

**eFigure 93.** Funnel plots of leisure sedentary behaviors and bladder cancer.

**eFigure 94.** Leave-one-out plots of leisure sedentary behaviors and bladder cancer.

**eFigure 95.** Forest plots of single-SNP analysis of leisure sedentary behaviors and bladder cancer.

**eFigures of glioma**

**eFigure 96.** Scatter plots of leisure sedentary behaviors and glioma.

**eFigure 97.** Funnel plots of leisure sedentary behaviors and glioma.

**eFigure 98.** Leave-one-out plots of leisure sedentary behaviors and glioma.

**eFigure 99.** Forest plots of single-SNP analysis of leisure sedentary behaviors and glioma.

# Supplementary Figures

## eFigure 1. Results of the univariable Mendelian randomization analyses of the association between television watching and endometrial cancer


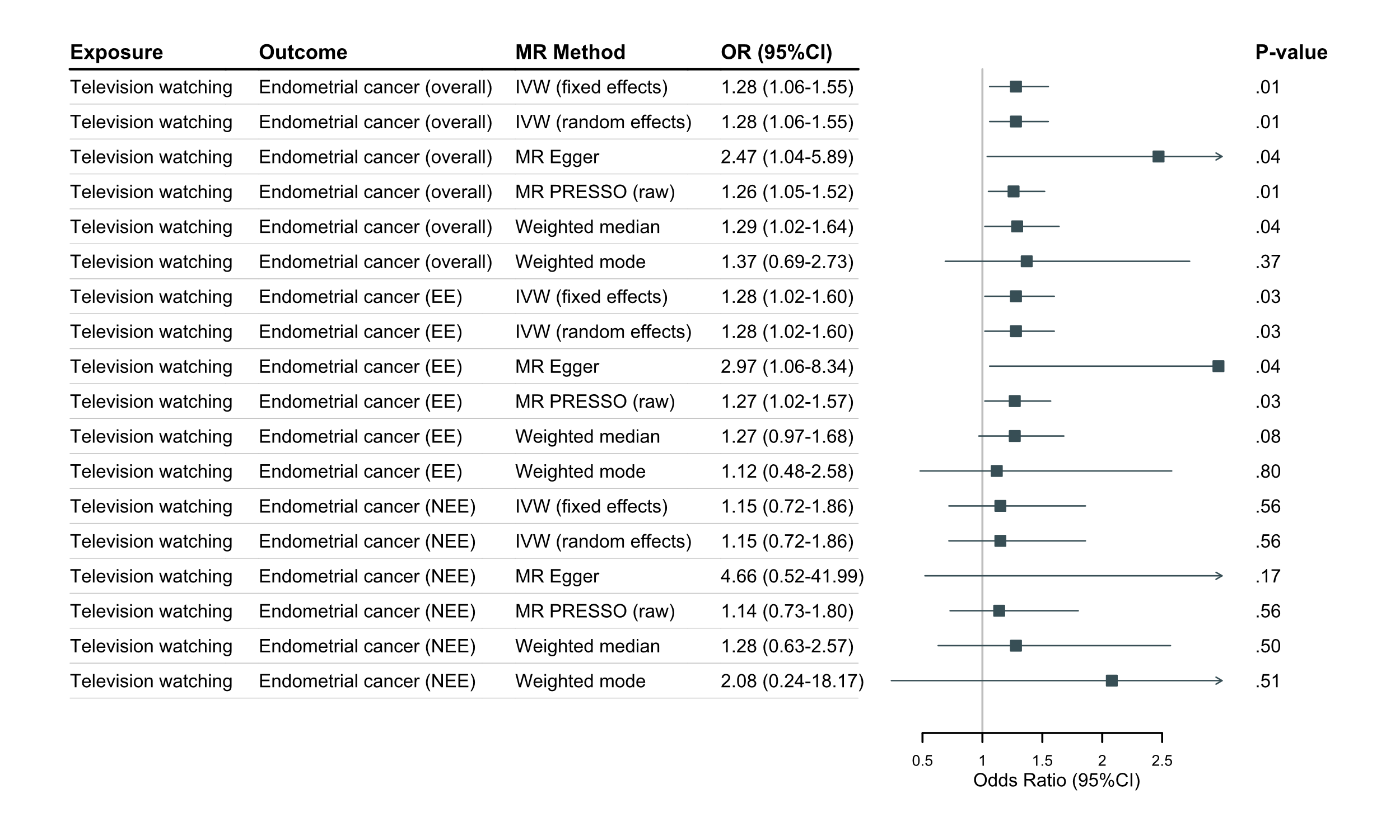


The forest plots show the results of the univariable Mendelian randomization analyses between sedentary television watching and endometrial cancer estimated with MR-IVW and sensitivity analyses. EE, endometrioid endometrial cancer; NEE, non-endometrioid endometrial cancer.

## eFigure 2. Results of the univariable Mendelian randomization analyses of the association between television watching and breast cancer


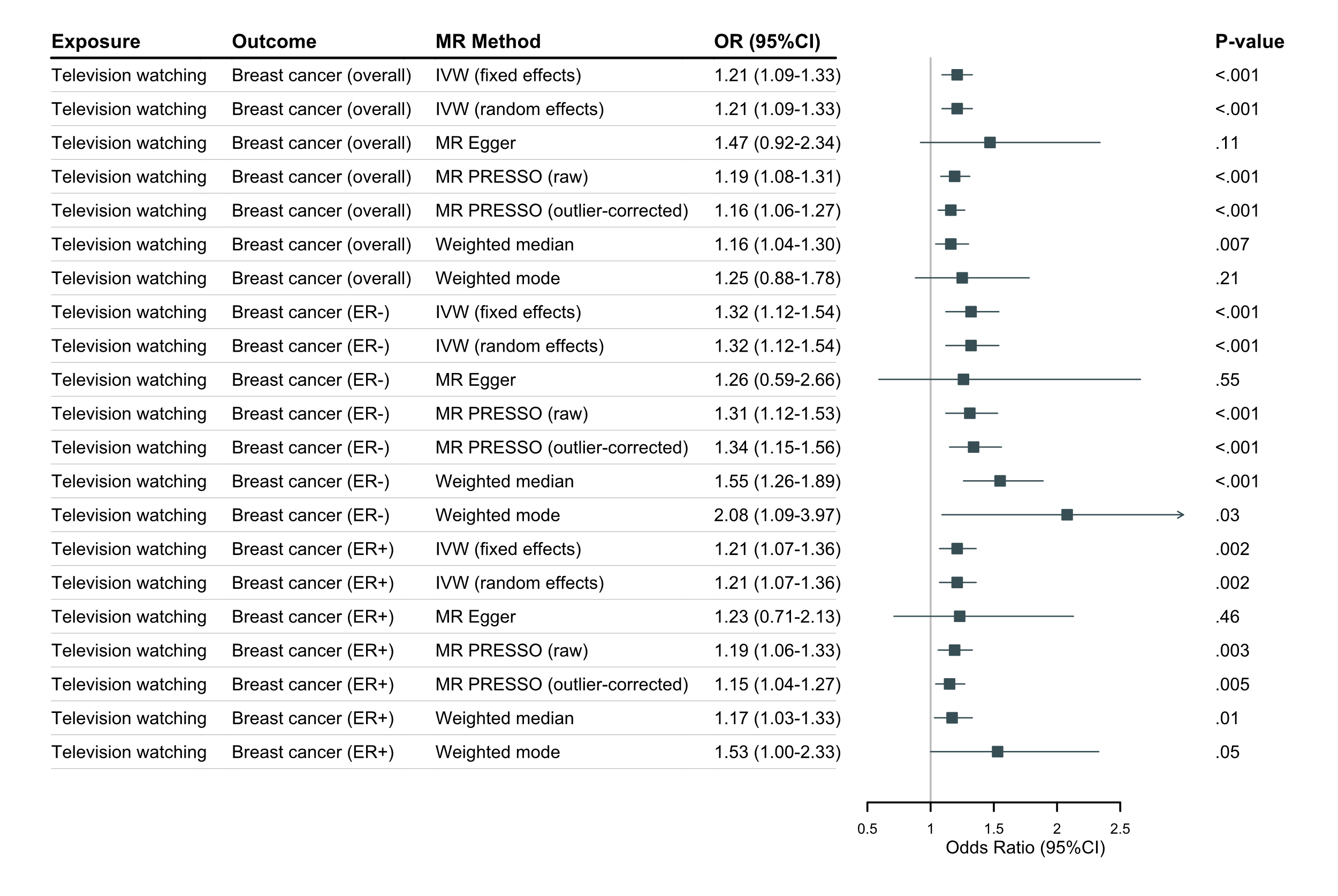


The forest plots show the results of the univariable Mendelian randomization analyses between sedentary television watching and breast cancer estimated with MR-IVW and sensitivity analyses. ER+, estrogen receptor positive breast cancer; ER–, estrogen receptor negative breast cancer.

## eFigure 3. Results of the univariable Mendelian randomization analyses of the association between television watching and ovarian cancer


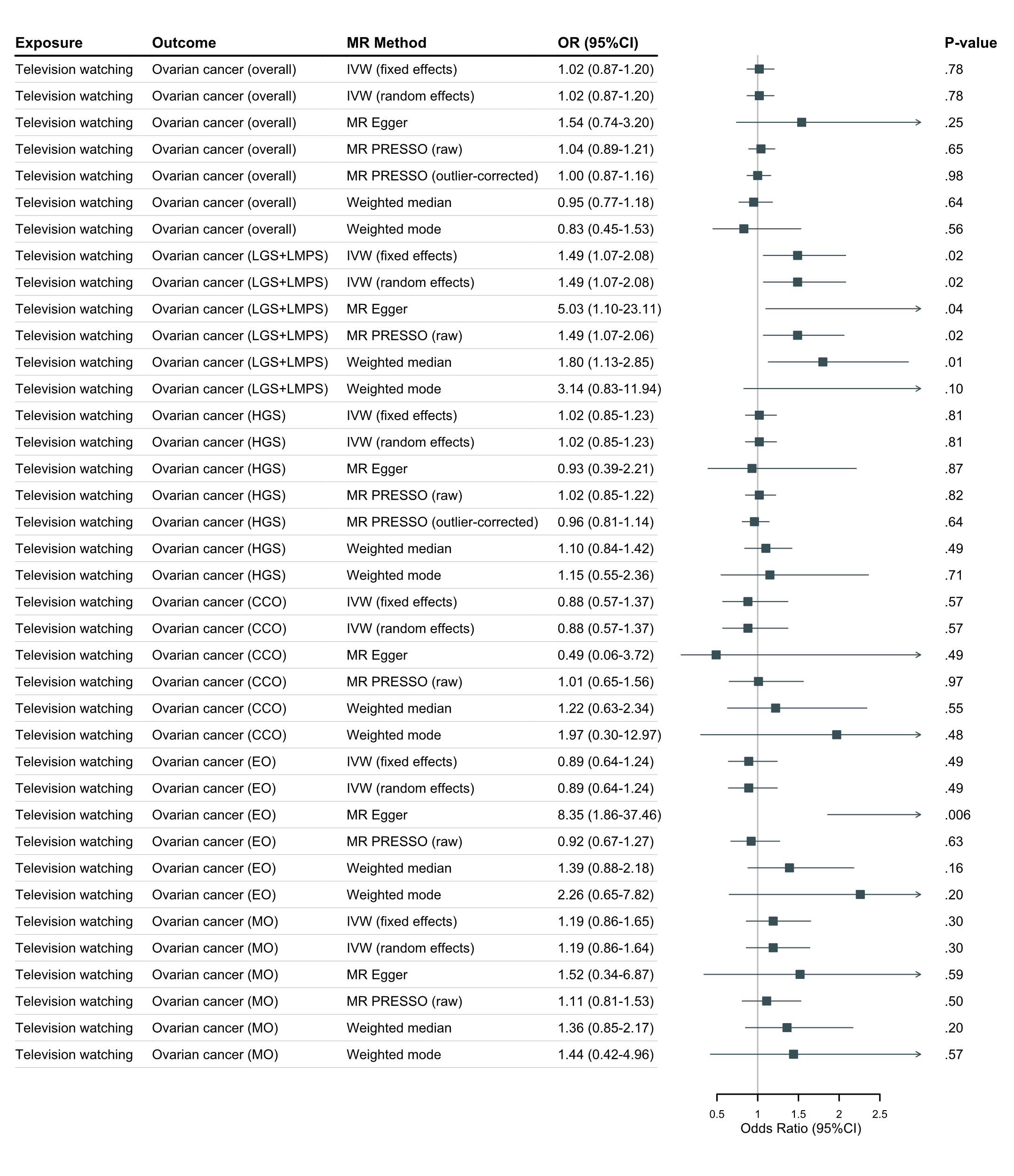


The forest plots show the results of the univariable Mendelian randomization analyses between sedentary watching and ovarian cancer estimated with MR-IVW and sensitivity analyses. LGS+LMPS, low grade and low malignant potential Serous ovarian cancer; HGS, high grade serous ovarian cancer; CCO, clear cell ovarian cancer; EO, Endometrioid ovarian cancer; MO, mucinous ovarian cancer.

## Summary illustration of eFigures 4-79

eFigure 1-76 include funnel plots, scatter plots, leave-one-out plots, forest plots of single-SNP analysis between leisure sedentary behaviors and 15 site-specific cancers.

**Illustration of funnel plots,**

For funnel plots of leisure sedentary behaviors and 15 site-specific cancers, β-value of instrument variables are displayed on the X-axis. 1/SE of instrument variables are displayed on the Y-axis. Funnel plots show the associations between the single-SNP causal effects of leisure sedentary behaviors on 15 site-specific cancers and the inverse of the standard error of the causal estimate. Vertical lines represent the MR estimations including all SNPs for five different Mendelian randomization analysis methods.

Specifically, asymmetric funnel plot indicated heterogeneity in instrument variables, which can be mutually verified with the results of heterogeneity test. Some points that deviate significantly from the population in the funnel plot can be removed.

**Illustration of scatter plots,**

Scatter plot including the Mendelian randomization estimates between leisure sedentary behaviors (television watching, computer use and driving) and 15 site-specific cancers for the main analyses, in which a threshold of *P* < 5×10^−8^ was used for the selection of variants.

In these plots, points represent the selected instrument variables, the line on each point reflects the 95% confidence interval, the variants’ effects and standard error on leisure sedentary behaviors are displayed on the X-axis, the variants’ effects and standard error on cancers on the Y-axis. The plots included 5 lines in different colors, which stand for the regression line of inverse-variance-weighted (IVW) (fixed effects meta-analysis), IVW (random effects meta-analysis), MR-Egger analysis, weighted-median analysis and weighted-mode analysis, respectively. The positive slope of the fitted curve indicates a positive association between exposure and outcome. SNP denotes single nucleotide polymorphism, MR denotes Mendelian randomization.

**Illustration of forest plots of leave-one-out plots,**

For forest plots of leave-one-out analysis between leisure sedentary behaviors (television watching, computer use and driving) and 15 site-specific cancers. The Mendelian randomization effect size and standard error of leisure sedentary behaviors (television watching, computer use and driving) on 15 site-specific cancers after leaving one specific SNP out are displayed on the X-axis. The SNPs for leisure sedentary behaviors that are left out are listed on the Y-axis.

Leave-one-out test is mainly to calculate the MR results of the remaining instrument variables after removing instrument variables one by one. If the estimated MR results of remaining instrument variables are significantly different from the total results, it indicates that the MR results are sensitive to this instrument variable.

**Illustration of forest plots of single-SNP analysis,**

For forest plots of single-SNP analysis between leisure sedentary behaviors and 15 site-specific cancers, the Mendelian Randomization effect size and standard error of 3 leisure sedentary behaviors (television watching, computer use and driving) on 15 site-specific cancers are displayed on the X-axis. The different genetic variants for leisure sedentary behaviors are listed on the Y-axis.

Each horizontal solid line in the forest plots reflects 95% confidence interval of the odds ratio (points) estimated by a single SNP using the Wald ratio method. Some solid lines are completely on the left of zero, indicating that the result estimated by this SNP is that leisure sedentary behaviors can reduce the risk of the specific cancer. Some solid lines are completely on the right of zero, indicating that the result estimated by this SNP is that leisure sedentary behaviors can increase the risk of the specific cancer, while those crossing 0 indicate that the result is not significant. Therefore, it may be inappropriate for us to look at the results of a SNP alone. Reasonable results can only be obtained by combining the single results, that is the lowest red line, which reflects the causal effect of exposure on the outcome under Inverse-variance-weighted (IVW) (fixed-effects), IVW (random-effects), MR-Egger, Weighted-median and Weighted-mode methods.

## eFigures of endometrial cancer (overall)

### eFigure 4. Funnel plots of leisure sedentary behaviors and endometrial cancer (overall)^a^

^
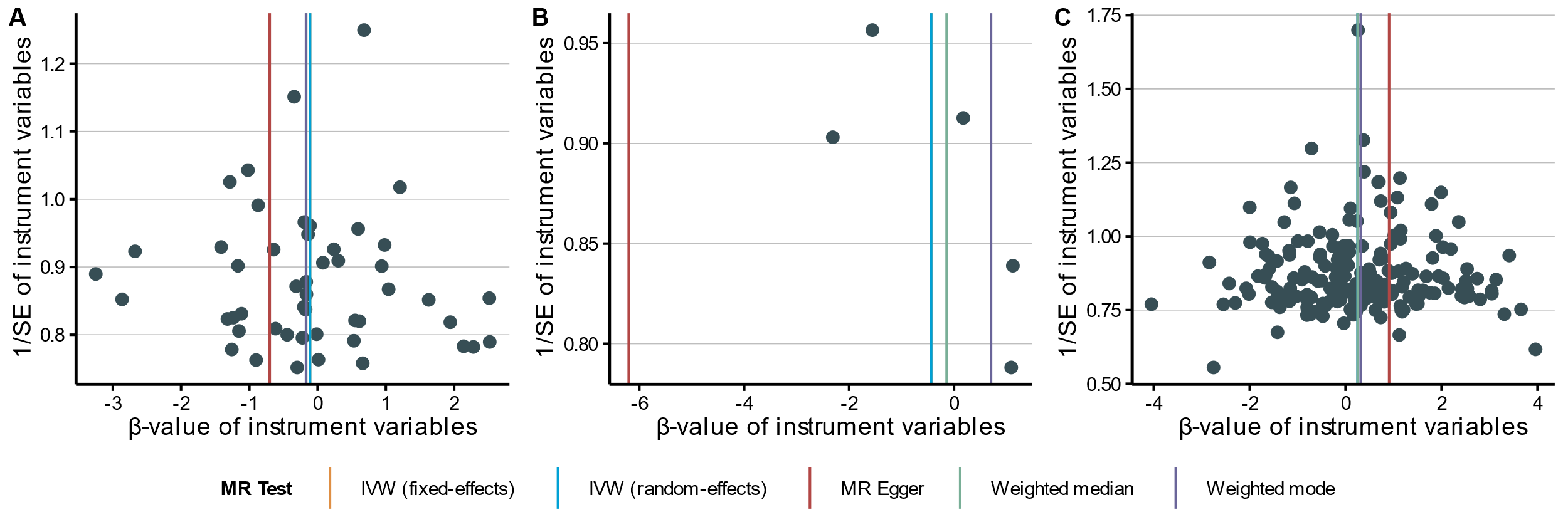
^

a. endometrial cancer (overall) stands for endometrial cancer including both endometrioid and non-endometrioid endometrial cancer.

Funnel plots with colored vertical lines representing total MR estimation of causal associations between (A) computer using and overall endometrial cancer, (B) driving and overall endometrial cancer, (C) television watching and overall endometrial cancer.

### eFigure 5. Scatter plots of leisure sedentary behaviors and endometrial cancer (overall)^a^

^
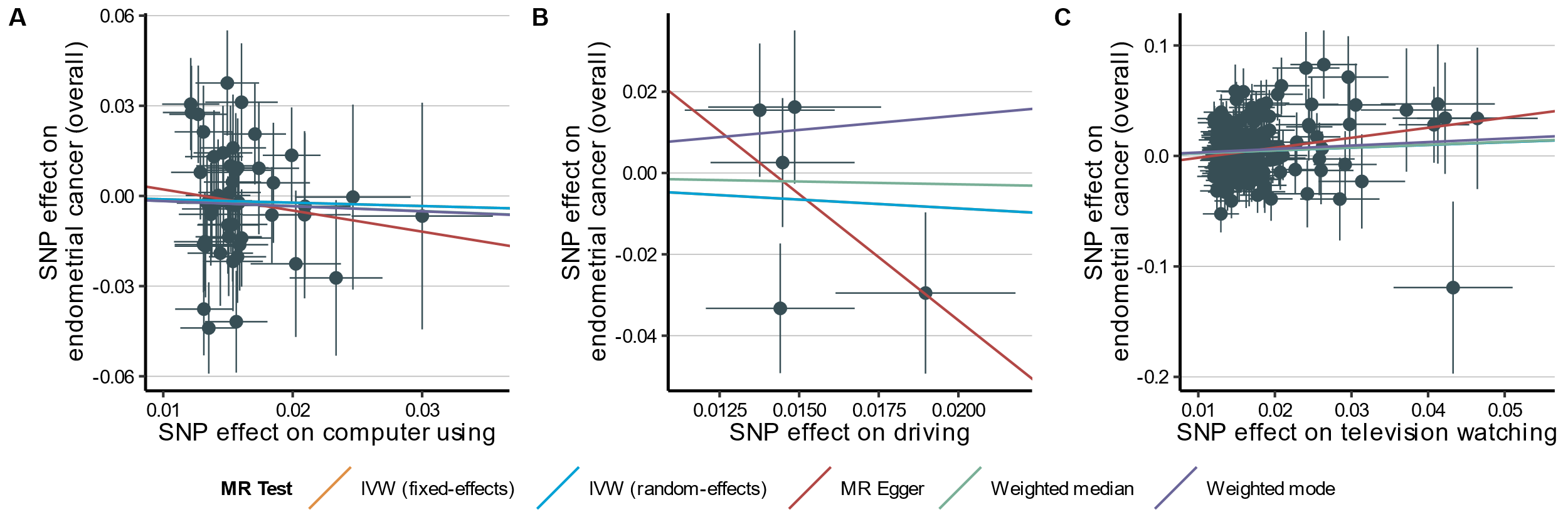
^

a. endometrial cancer (overall) stands for breast cancer including both endometrioid and non-endometrioid endometrial cancer.

Scatter plots with colored lines representing results of each mendelian randomization sensitivity analysis between (A) computer using and overall endometrial cancer, (B) driving and overall endometrial cancer, (C) television watching and overall endometrial cancer.

### eFigure 6. Leave-one-out plots of leisure sedentary behaviors and endometrial cancer (overall)^a^


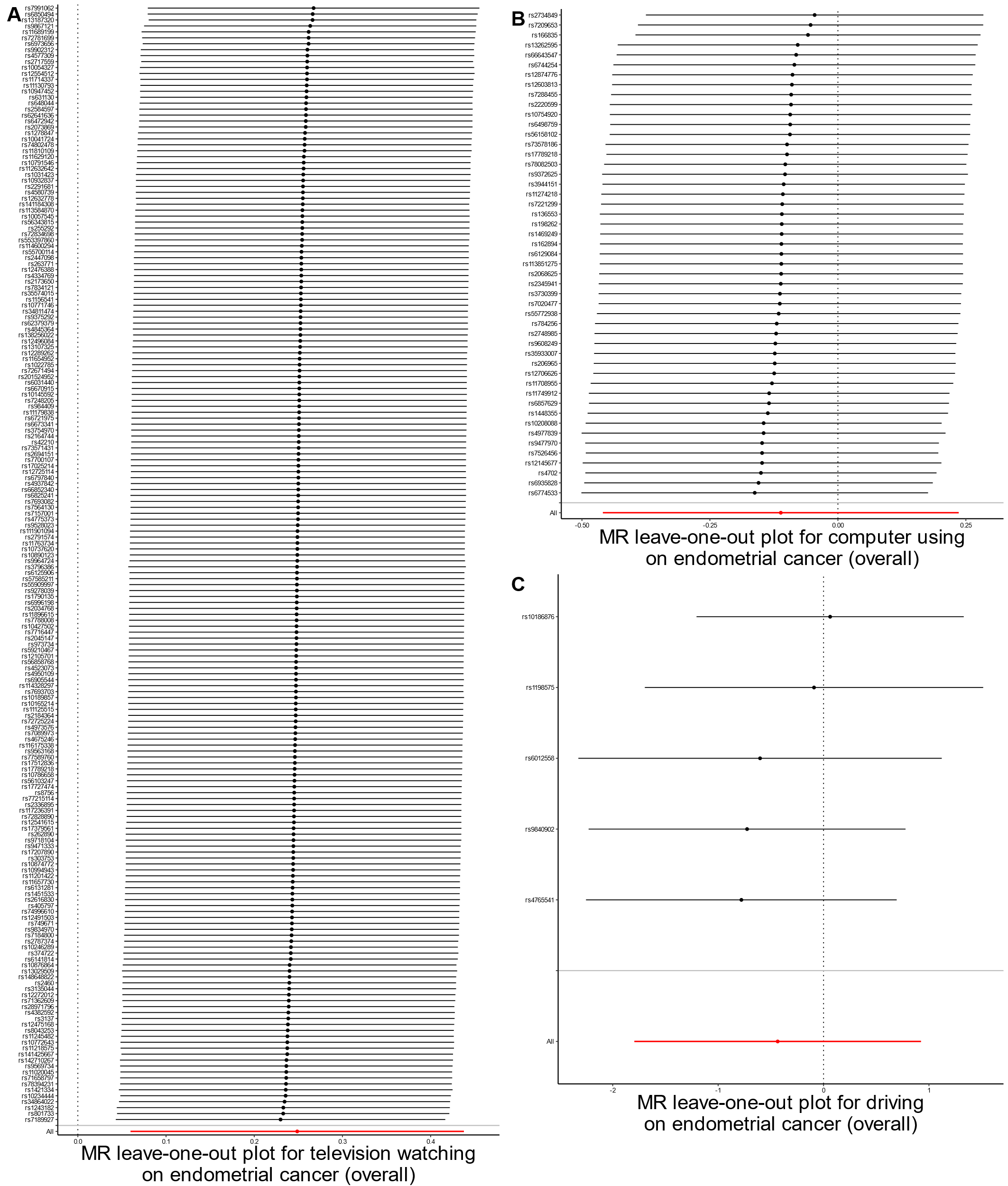


a. endometrial cancer (overall) stands for endometrial cancer including both endometrioid and non-endometrioid endometrial cancer.

Leave-one-out plot of Mendelian randomization sensitivity analysis between (A) television watching and overall endometrial cancer, (B) computer using and overall endometrial cancer, (C) driving and overall endometrial cancer.

### eFigure 7. Forest plots of single-SNP analysis of leisure sedentary behaviors and endometrial cancer (overall)^a^


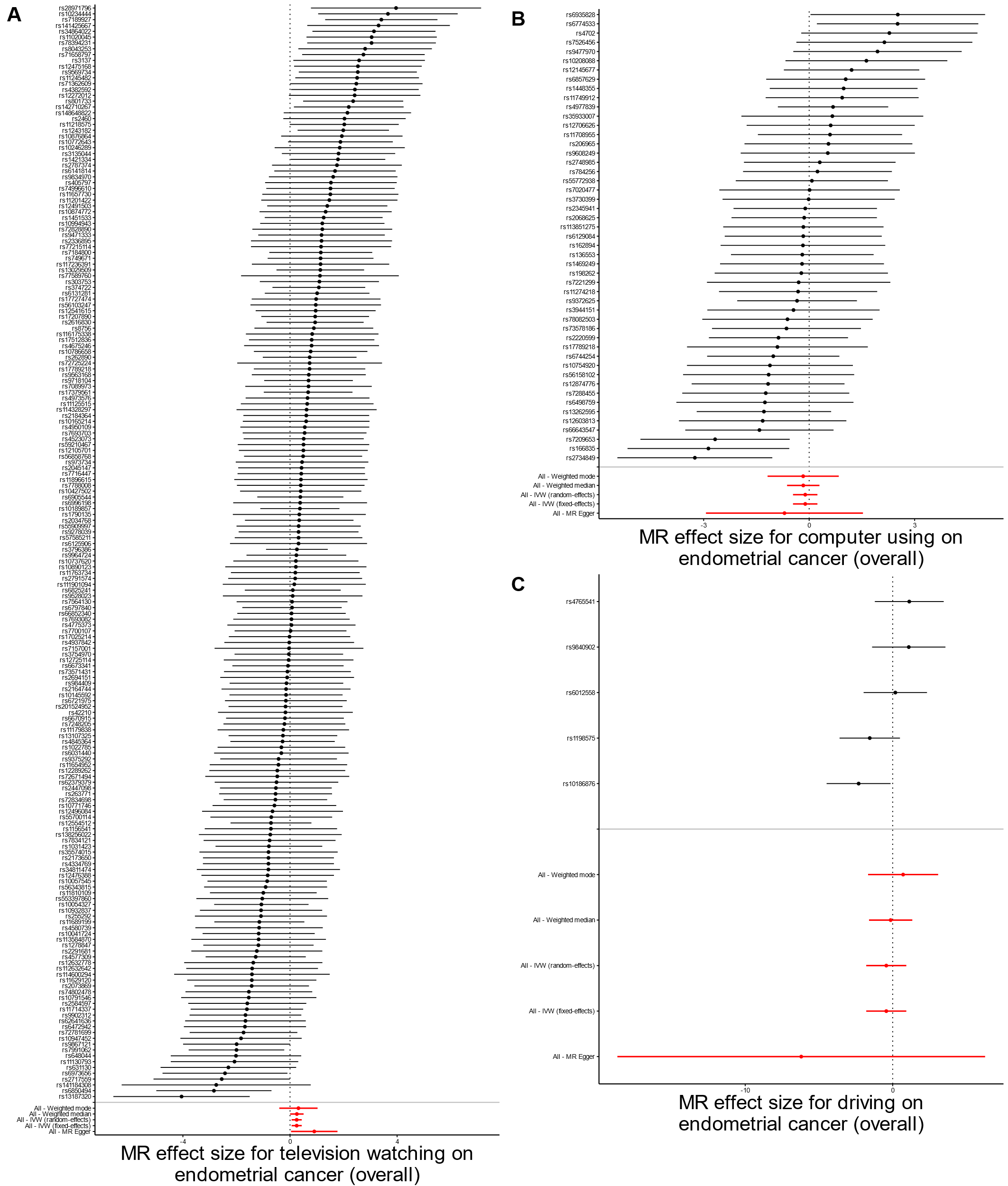


a. endometrial cancer (overall) stands for endometrial cancer including both endometrioid and non-endometrioid endometrial cancer.

The MR single-SNP analysis plots the Wald estimate of causal association between (A) television watching and overall endometrial cancer, (B) computer using and overall endometrial cancer, (C) driving and overall endometrial cancer.

## eFigures of endometrial cancer (EE)

### eFigure 8. Funnel plots of leisure sedentary behaviors and endometrioid endometrial cancer


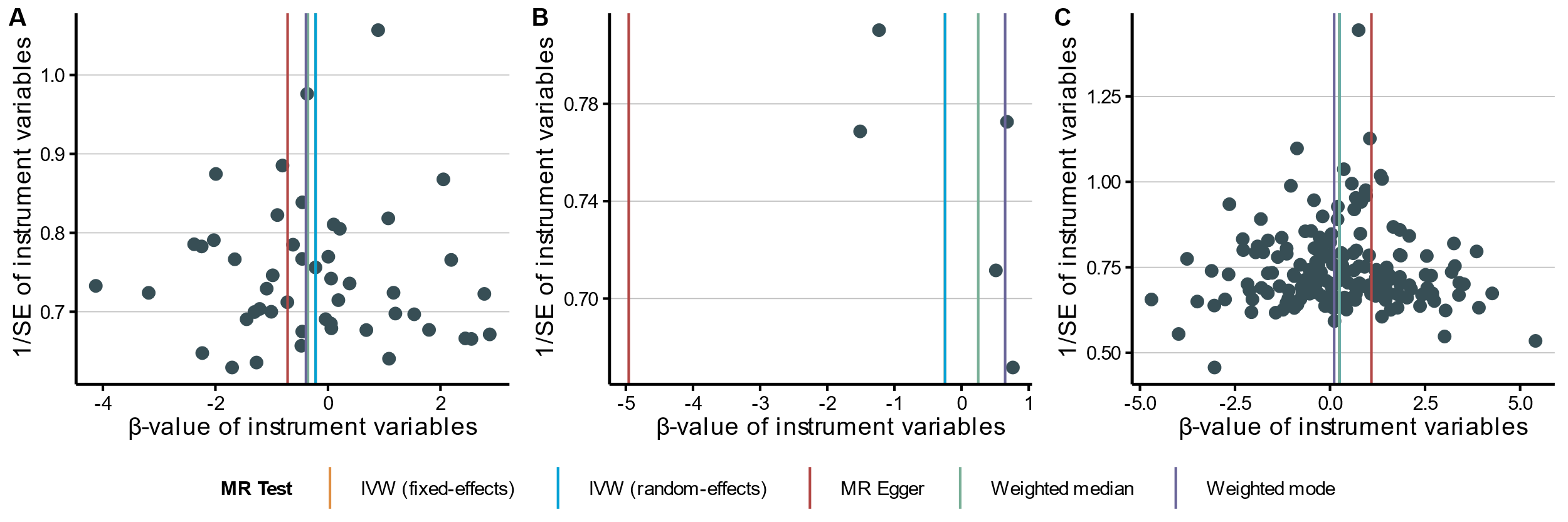


Funnel plots with colored vertical lines representing total MR estimation of causal associations between (A) computer using and endometrioid endometrial cancer, (B) driving and endometrioid endometrial cancer, (C) television watching and endometrioid endometrial cancer.

### eFigure 9. Scatter plots of leisure sedentary behaviors and endometrioid endometrial cancer

^
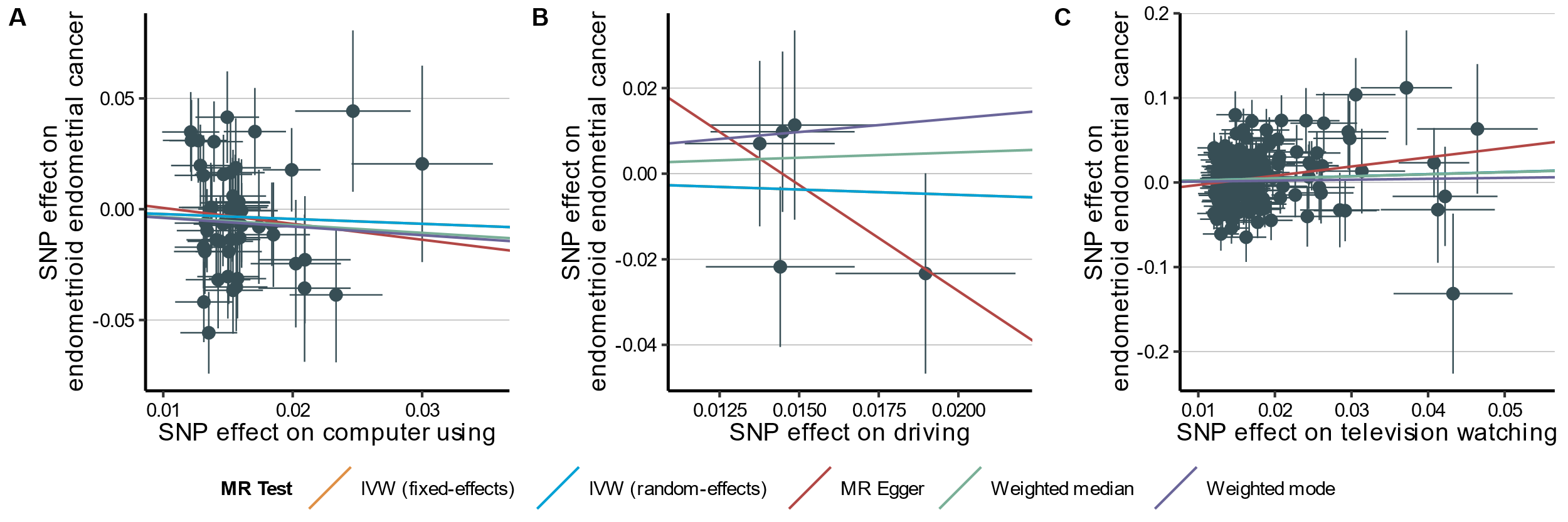
^

Scatter plots with colored lines representing results of each mendelian randomization sensitivity analysis between (A) computer using and endometrioid endometrial cancer, (B) driving and endometrioid endometrial cancer, (C) television watching and endometrioid endometrial cancer.

### eFigure 10. Leave-one-out plots of leisure sedentary behaviors and endometrioid endometrial cancer


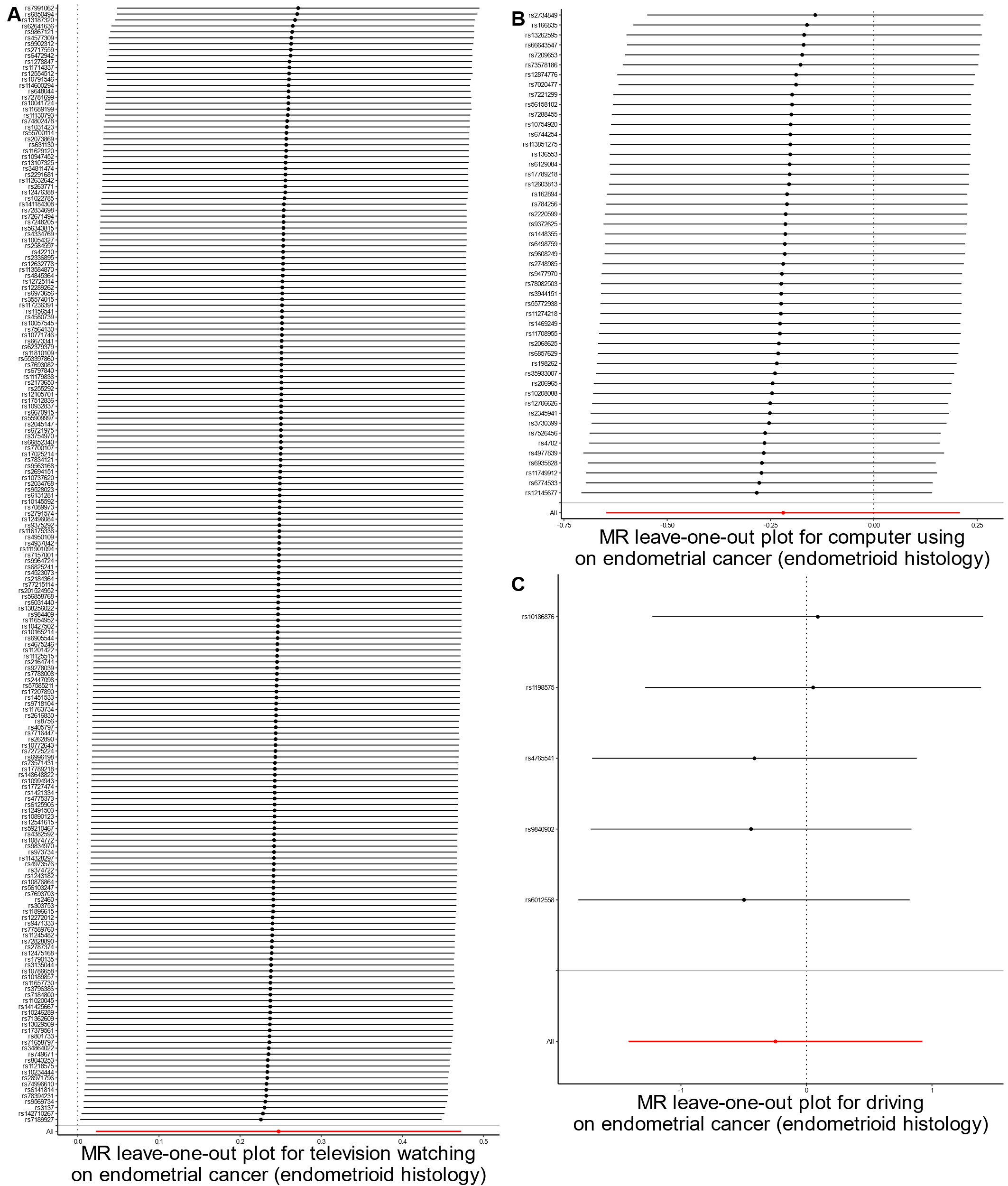


Leave-one-out plot of Mendelian randomization sensitivity analysis between (A) television watching and endometrioid endometrial cancer, (B) computer using and endometrioid endometrial cancer, (C) driving and endometrioid endometrial cancer.

### eFigure 11. Forest plots of single-SNP analysis of leisure sedentary behaviors and endometrioid endometrial cancer

^
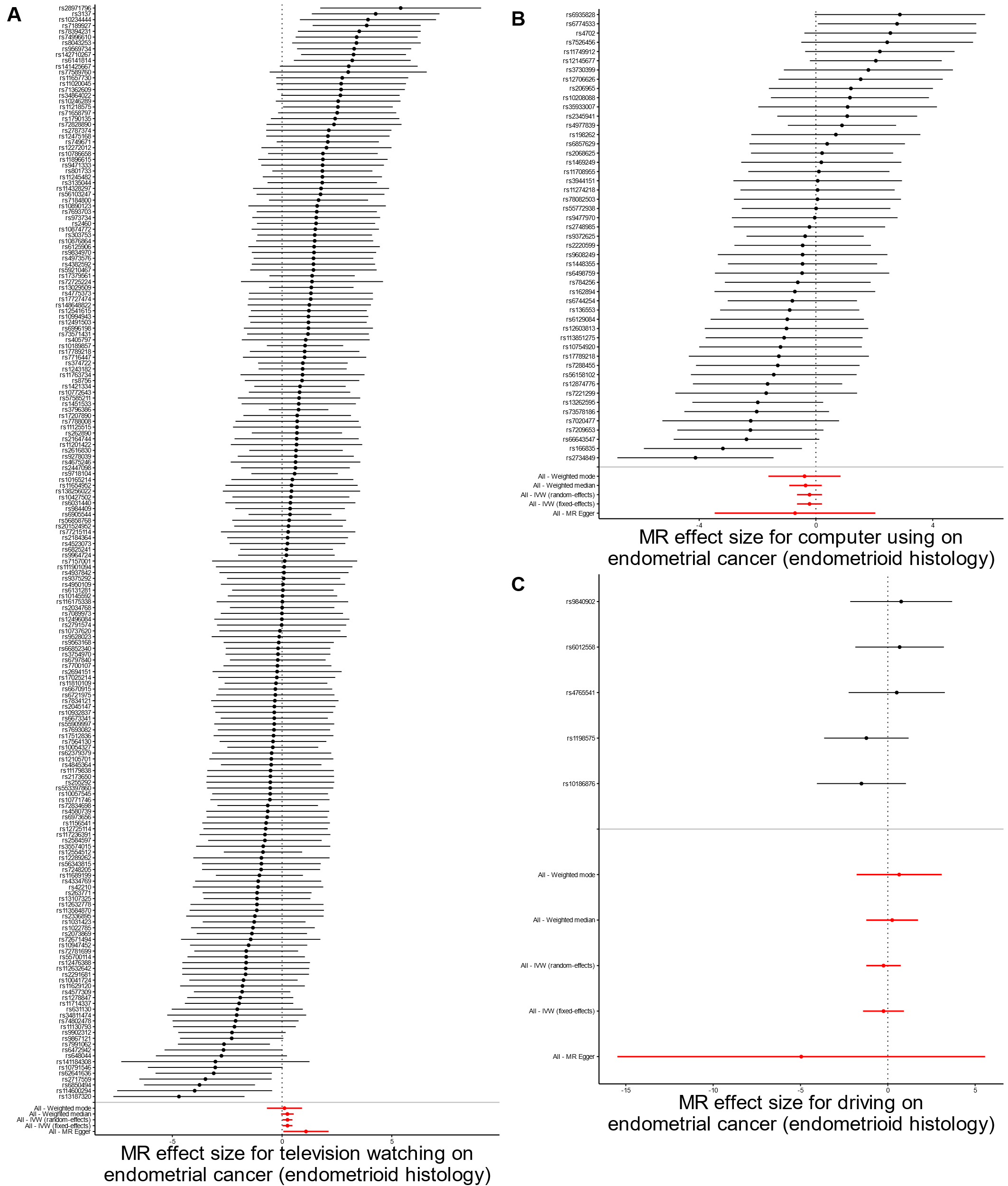
^

The MR single-SNP analysis plots the Wald estimate of causal association between (A) television watching and endometrioid endometrial cancer, (B) computer using and endometrioid endometrial cancer, (C) driving and endometrioid endometrial cancer.

## eFigures of endometrial cancer (non-EE)

### eFigure 12. Funnel plots of leisure sedentary behaviors and non-endometrioid endometrial cancer


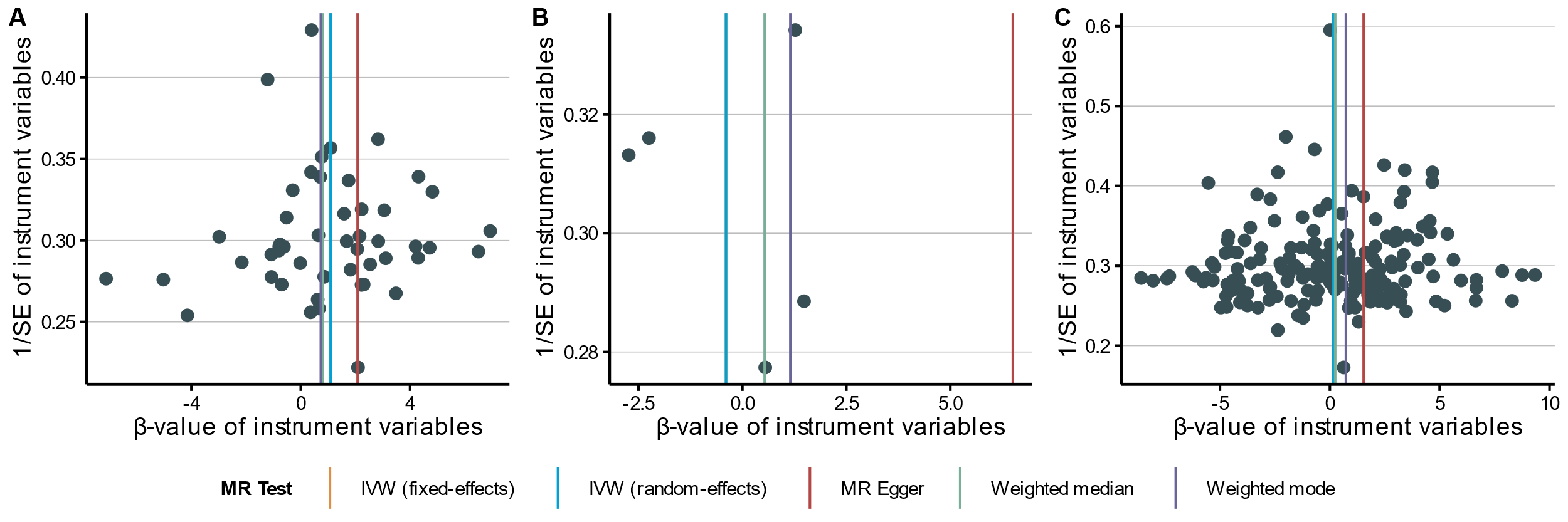


Funnel plots with colored vertical lines representing total MR estimation of causal associations between (A) computer using and non-endometrioid endometrial cancer, (B) driving and non-endometrioid endometrial cancer, (C) television watching and non-endometrioid endometrial cancer.

### eFigure 13. Scatter plots of leisure sedentary behaviors and non-endometrioid endometrial cancer


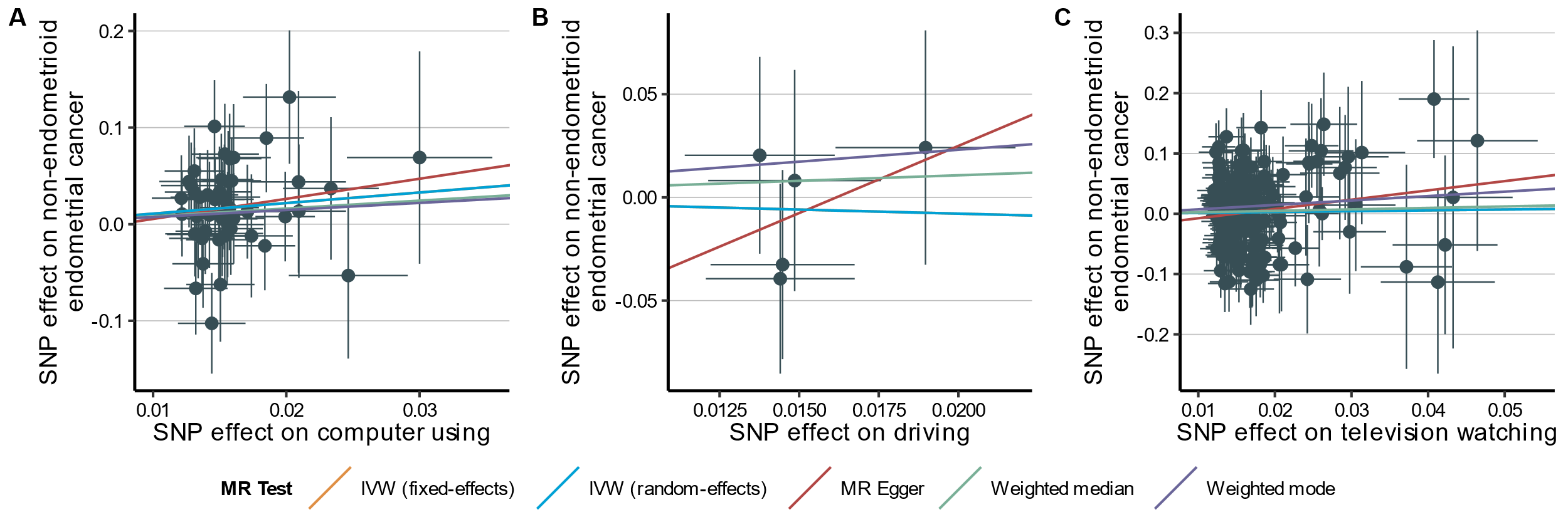


Scatter plots with colored lines representing results of each mendelian randomization sensitivity analysis between (A) computer using and non-endometrioid endometrial cancer, (B) driving and non-endometrioid endometrial cancer, (C) television watching and non-endometrioid endometrial cancer.

### eFigure 14. Leave-one-out plots of leisure sedentary behaviors and non-endometrioid endometrial cancer


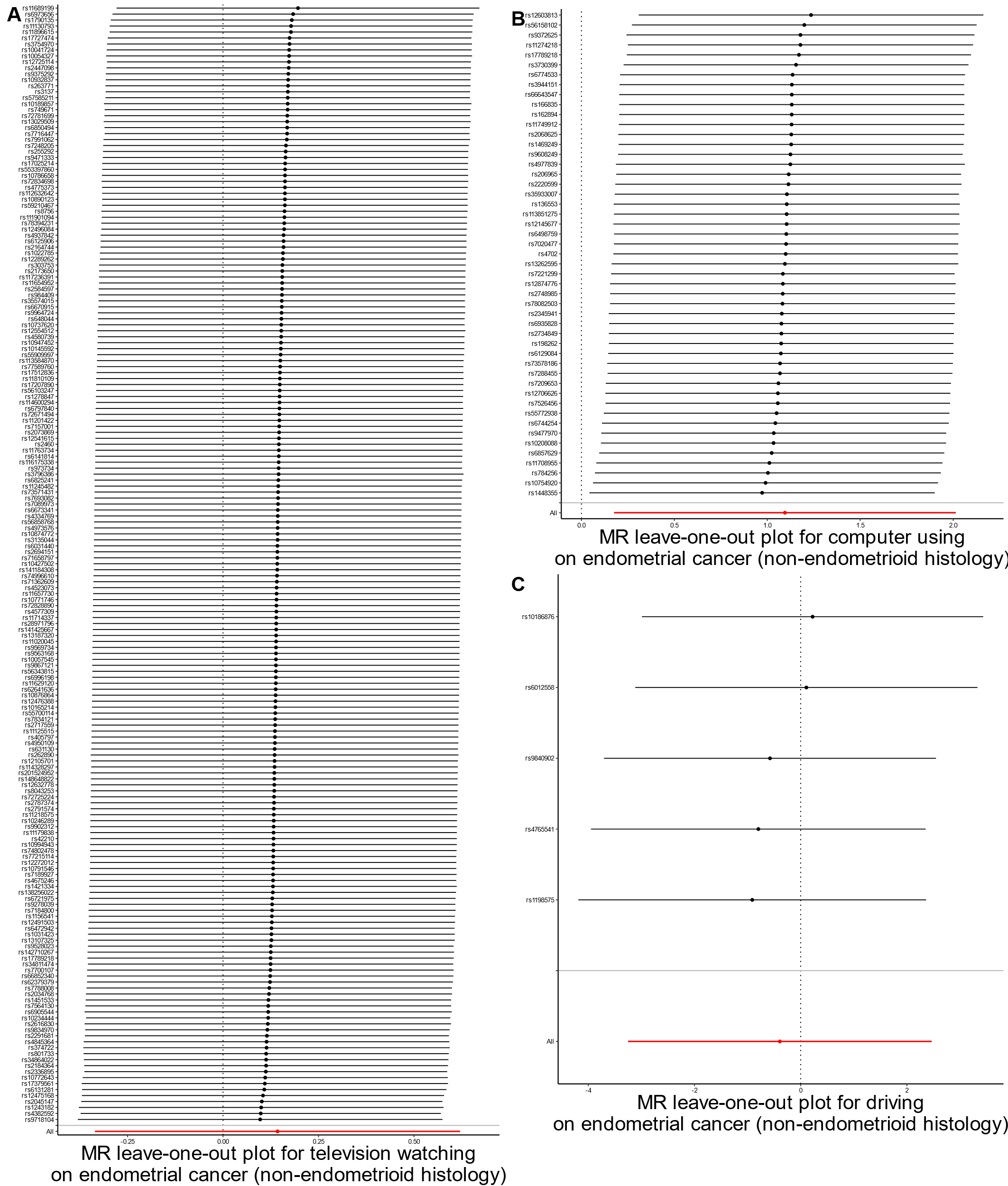


Leave-one-out plot of Mendelian randomization sensitivity analysis between (A) television watching and non-endometrioid endometrial cancer, (B) computer using and non-endometrioid endometrial cancer, (C) driving and non-endometrioid endometrial cancer.

### eFigure 15. Forest plots of single-SNP analysis of leisure sedentary behaviors and non-endometrioid endometrial cancer


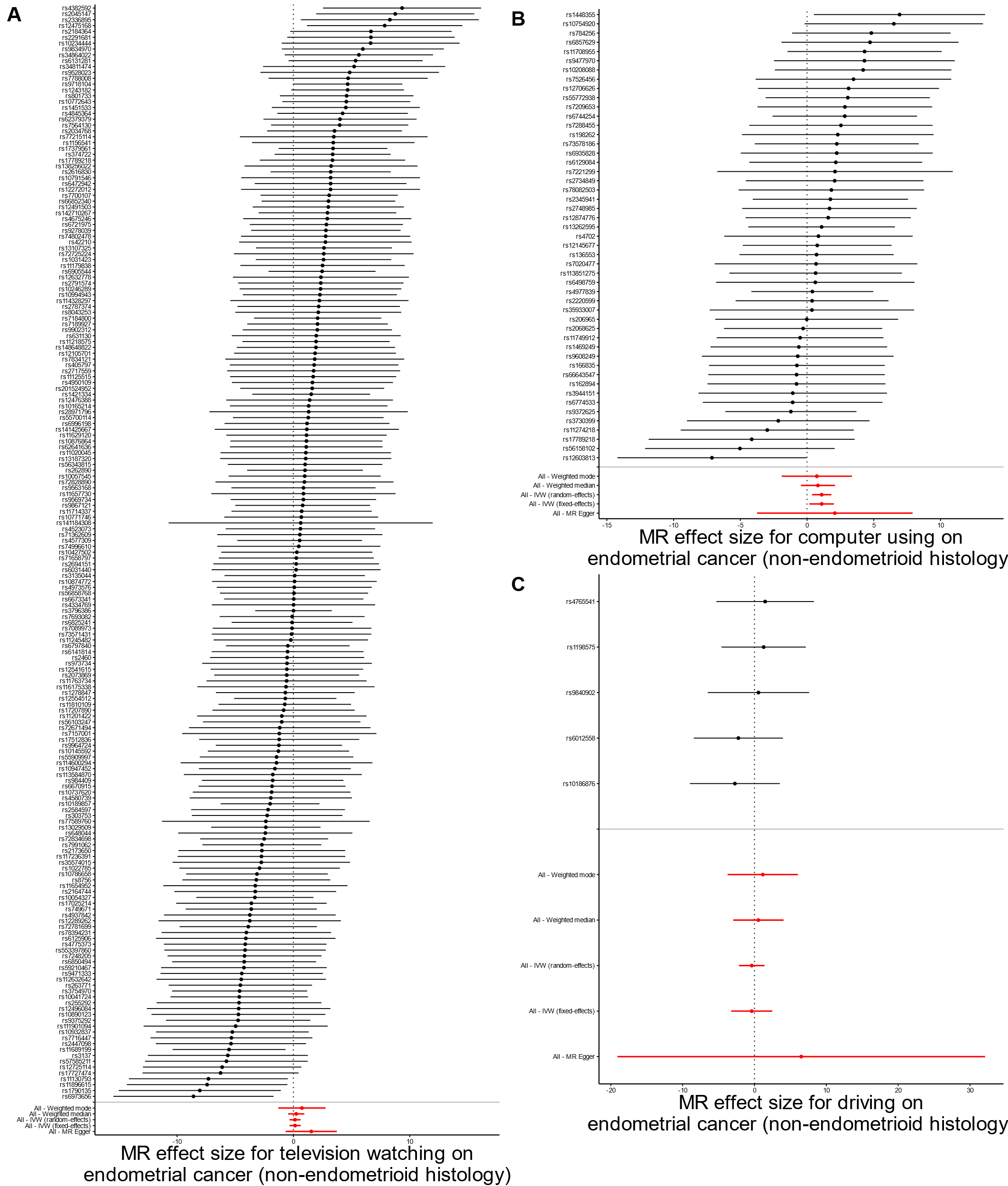


The MR single-SNP analysis plots the Wald estimate of causal association between (A) television watching and non-endometrioid endometrial cancer, (B) computer using and non-endometrioid endometrial cancer, (C) driving and non-endometrioid endometrial cancer.

## eFigures of breast cancer (overall)

### eFigure 16. Funnel plots of leisure sedentary behaviors and breast cancer (overall)^a^


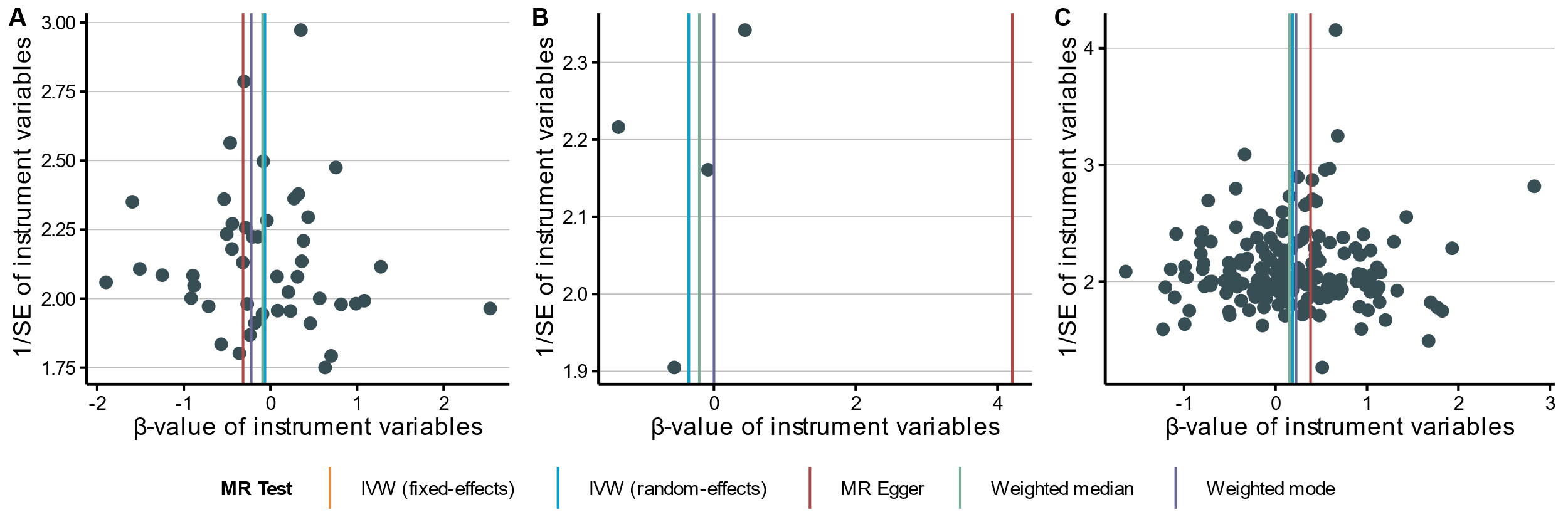


a. breast cancer (overall) stands for breast cancer including both estrogen receptor positive and estrogen receptor negative breast cancer.

Funnel plots with colored vertical lines representing total MR estimation of causal associations between (A) computer using and overall breast cancer, (B) driving and overall breast cancer, (C) television watching and overall breast cancer.

### eFigure 17. Scatter plots of leisure sedentary behaviors and breast cancer (overall)^a^

^
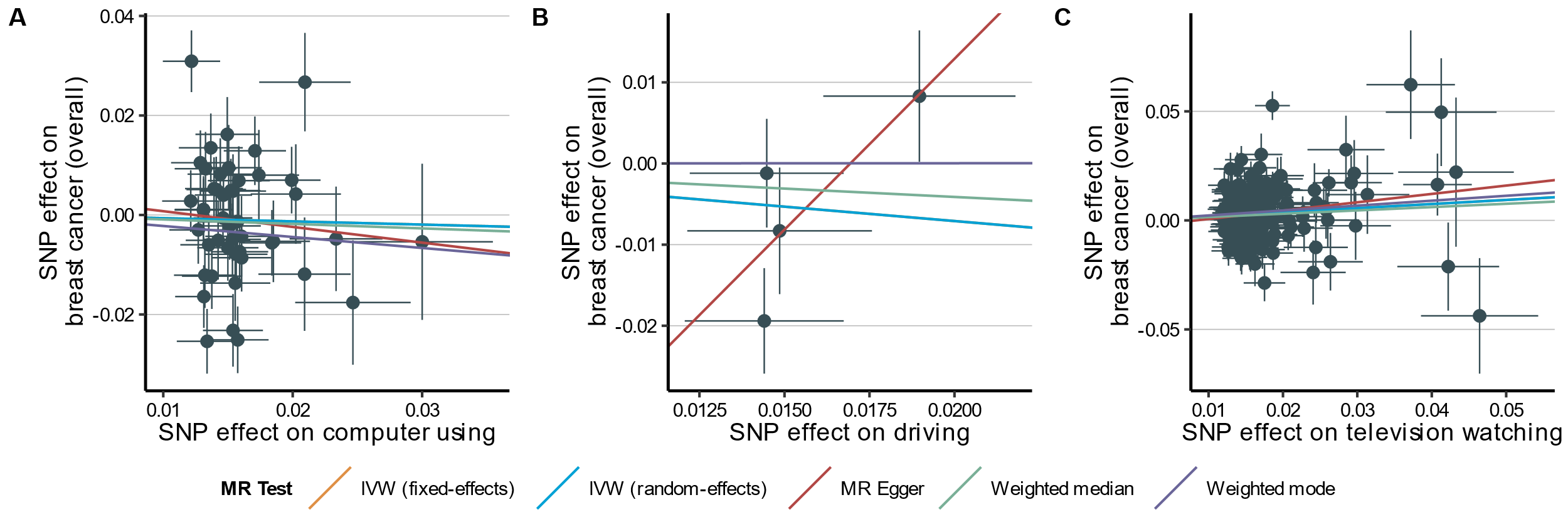
^

a. breast cancer (overall) stands for breast cancer including both estrogen receptor positive and estrogen receptor negative breast cancer.

Scatter plots with colored lines representing results of each mendelian randomization sensitivity analysis between (A) computer using and overall breast cancer, (B) driving and overall breast cancer, (C) television watching and overall breast cancer.

### eFigure 18. Leave-one-out plots of leisure sedentary behaviors and breast cancer (overall)^a^

^
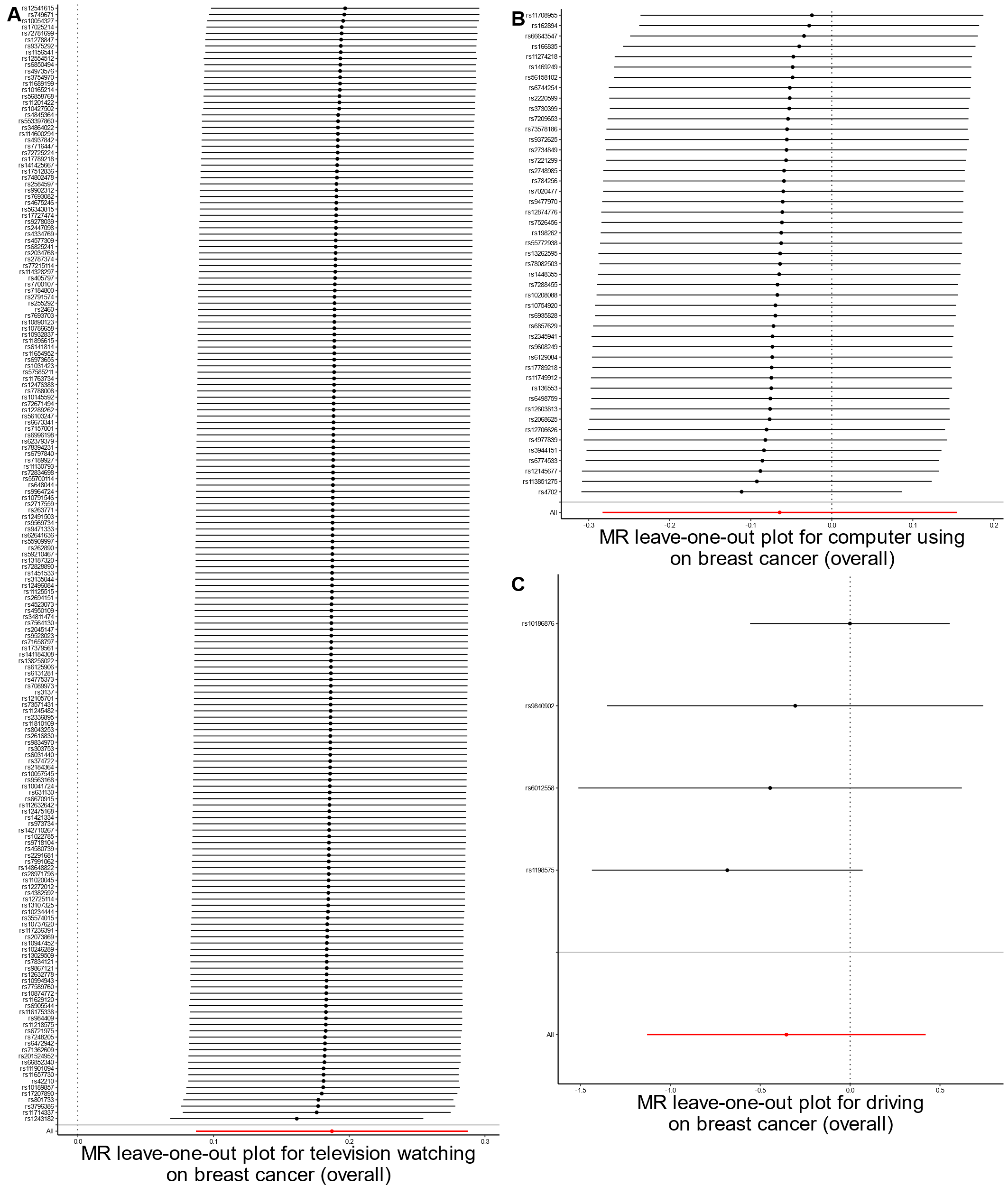
^

a. breast cancer (overall) stands for breast cancer including both estrogen receptor positive and estrogen receptor negative breast cancer.

Leave-one-out plot of Mendelian randomization sensitivity analysis between (A) television watching and overall breast cancer, (B) computer using and overall breast cancer, (C) driving and overall breast cancer.

### eFigure 19. Forest plots of single-SNP analysis of leisure sedentary behaviors and breast cancer (overall)^a^

^
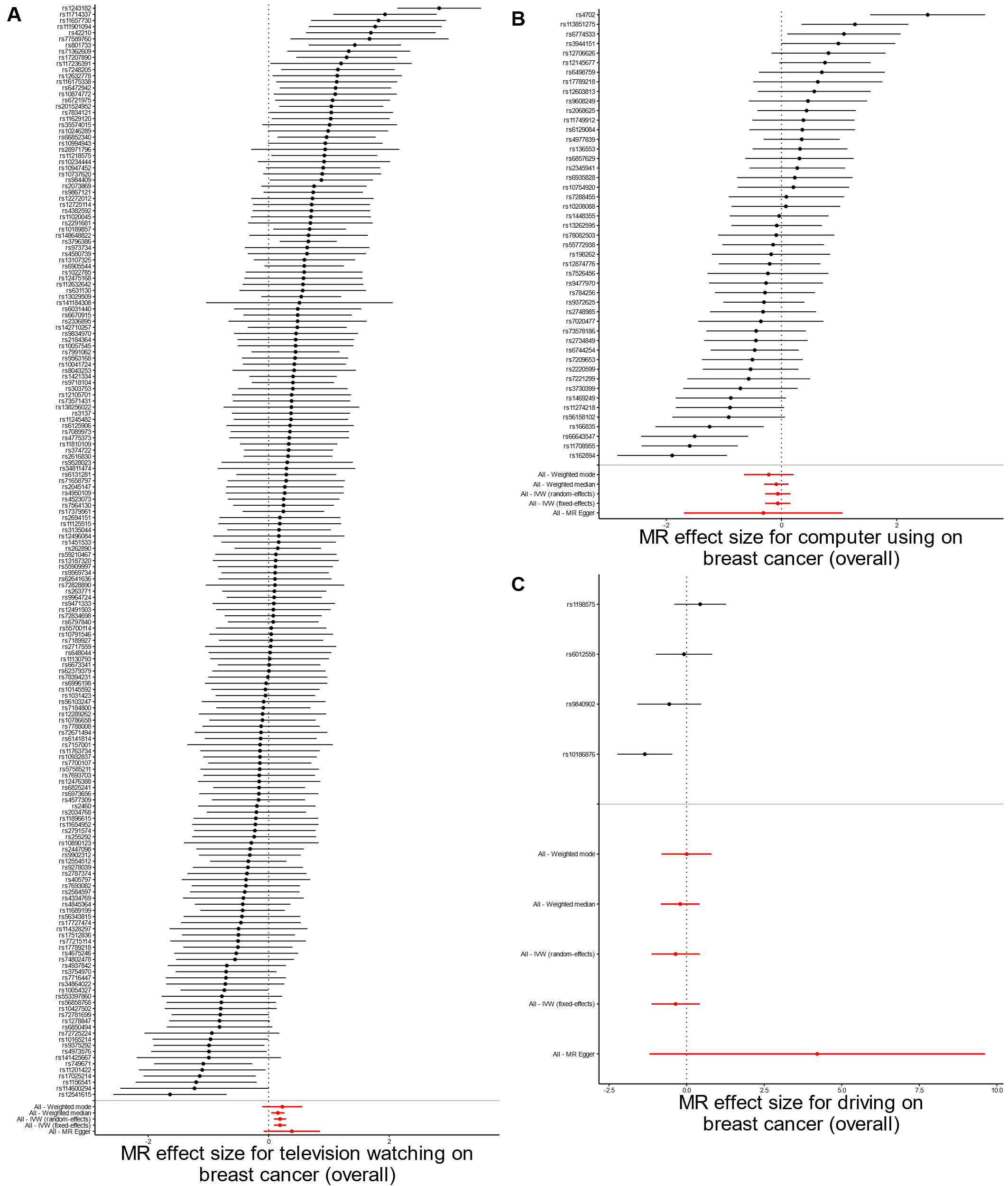
^

a. breast cancer (overall) stands for breast cancer including both estrogen receptor positive and estrogen receptor negative breast cancer.

The MR single-SNP analysis plots the Wald estimate of causal association between (A) television watching and overall breast cancer, (B) computer using and overall breast cancer, (C) driving and overall breast cancer.

## eFigures of breast cancer (ER–)

### eFigure 20. Funnel plots of leisure sedentary behaviors and breast cancer (ER–)^a^


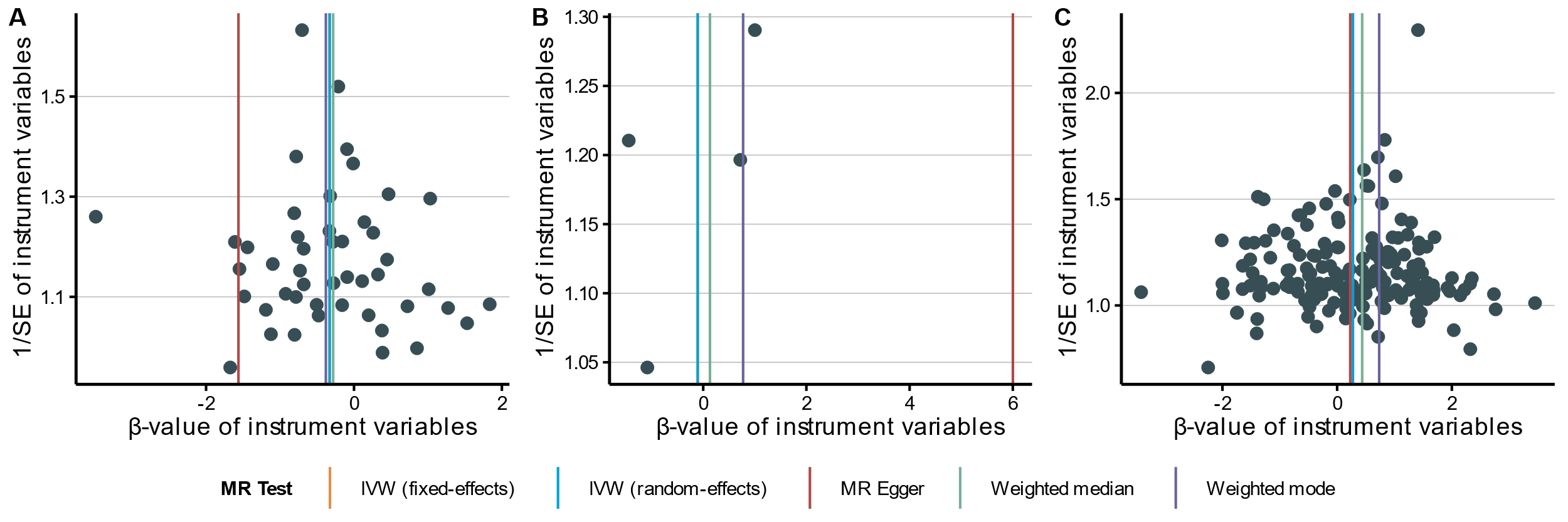


a. breast cancer (ER–) stands for estrogen receptor negative breast cancer.

Funnel plots with colored vertical lines representing total MR estimation of causal associations between (A) computer using and ER– breast cancer, (B) driving and ER– breast cancer, (C) television watching and ER– breast cancer.

### eFigure 21. Scatter plots of leisure sedentary behaviors and breast cancer (ER–)^a^

^
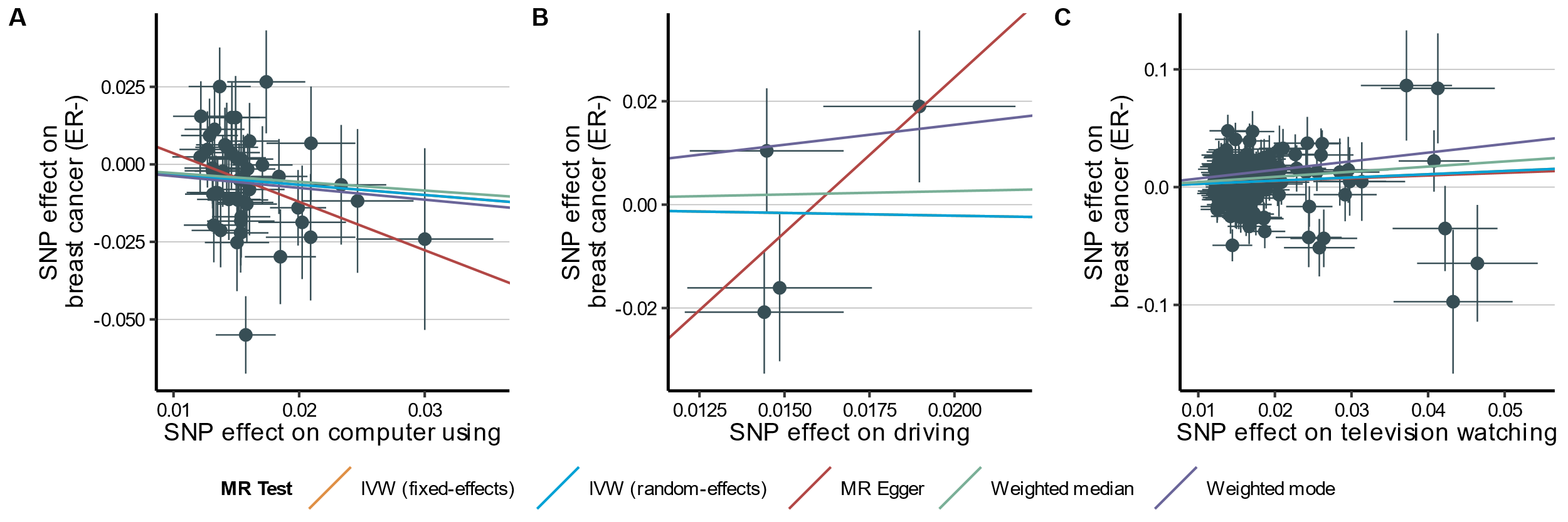
^

a. breast cancer (ER–) stands for estrogen receptor negative breast cancer.

Scatter plots with colored lines representing results of each mendelian randomization sensitivity analysis between (A) computer using and ER– breast cancer, (B) driving and ER– breast cancer, (C) television watching and ER– breast cancer.

### eFigure 22. Leave-one-out plots of leisure sedentary behaviors and breast cancer (ER–)^a^


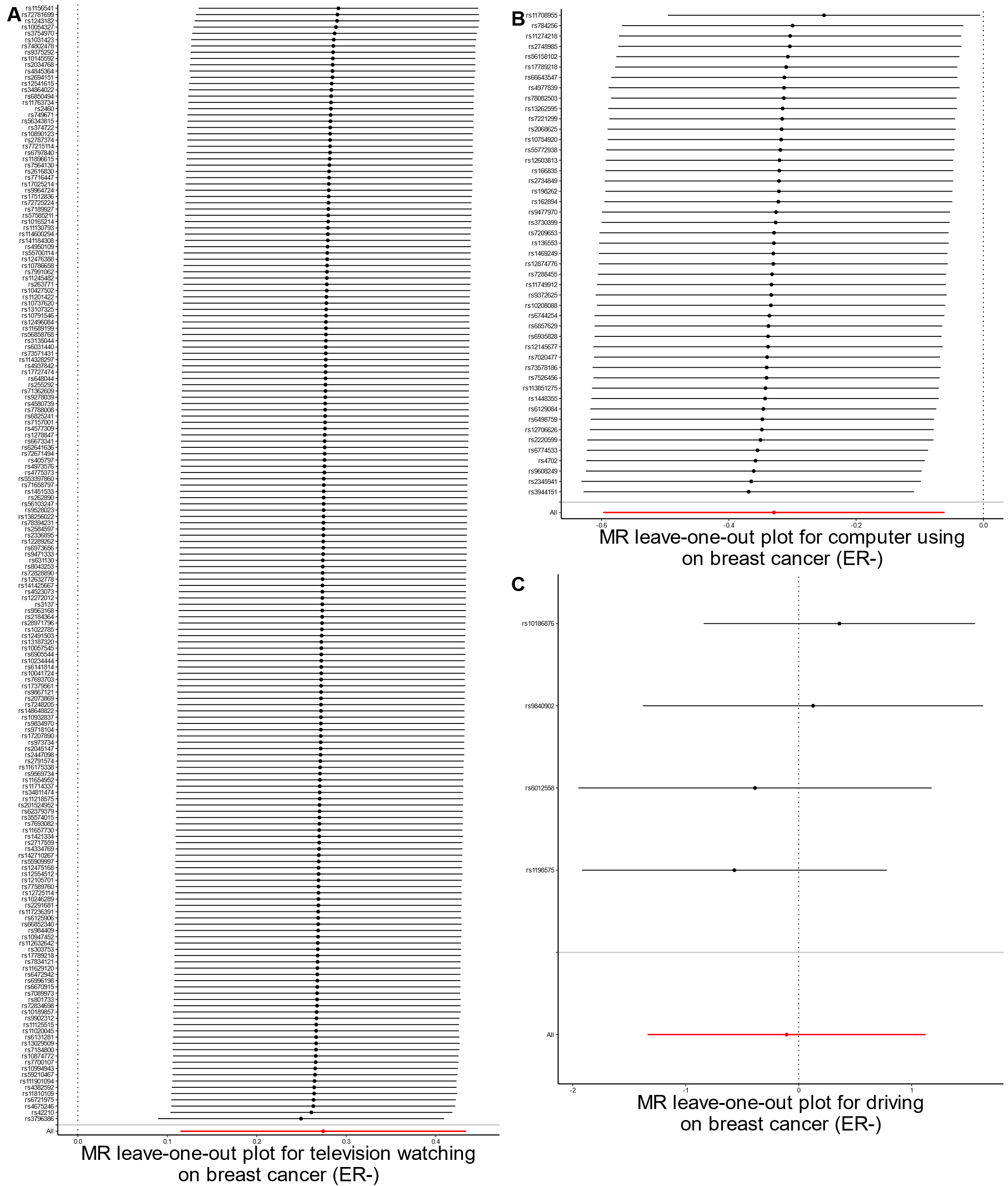


a. breast cancer (ER–) stands for estrogen receptor negative breast cancer.

Leave-one-out plot of Mendelian randomization sensitivity analysis between (A) television watching and ER– breast cancer, (B) computer using and ER– breast cancer, (C) driving and ER– breast cancer.

### eFigure 23. Forest plots of single-SNP analysis of leisure sedentary behaviors and breast cancer (ER–)^a^


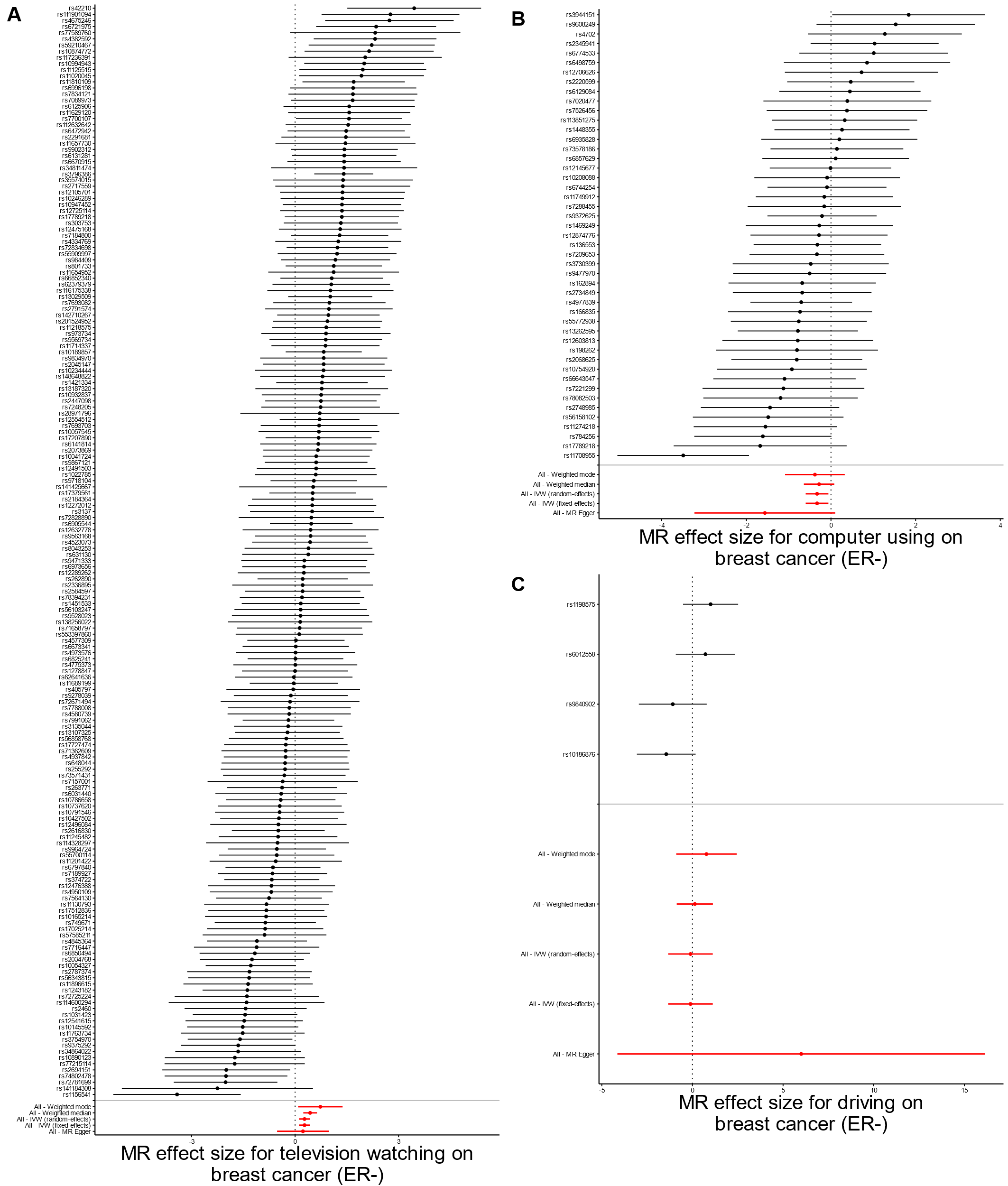


a. breast cancer (ER–) stands for estrogen receptor negative breast cancer.

The MR single-SNP analysis plots the Wald estimate of causal association between (A) television watching and ER– breast cancer, (B) computer using and ER– breast cancer, (C) driving and ER– breast cancer.

## eFigures of breast cancer (ER+)

### eFigure 24. Funnel plots of leisure sedentary behaviors and breast cancer (ER+)^a^


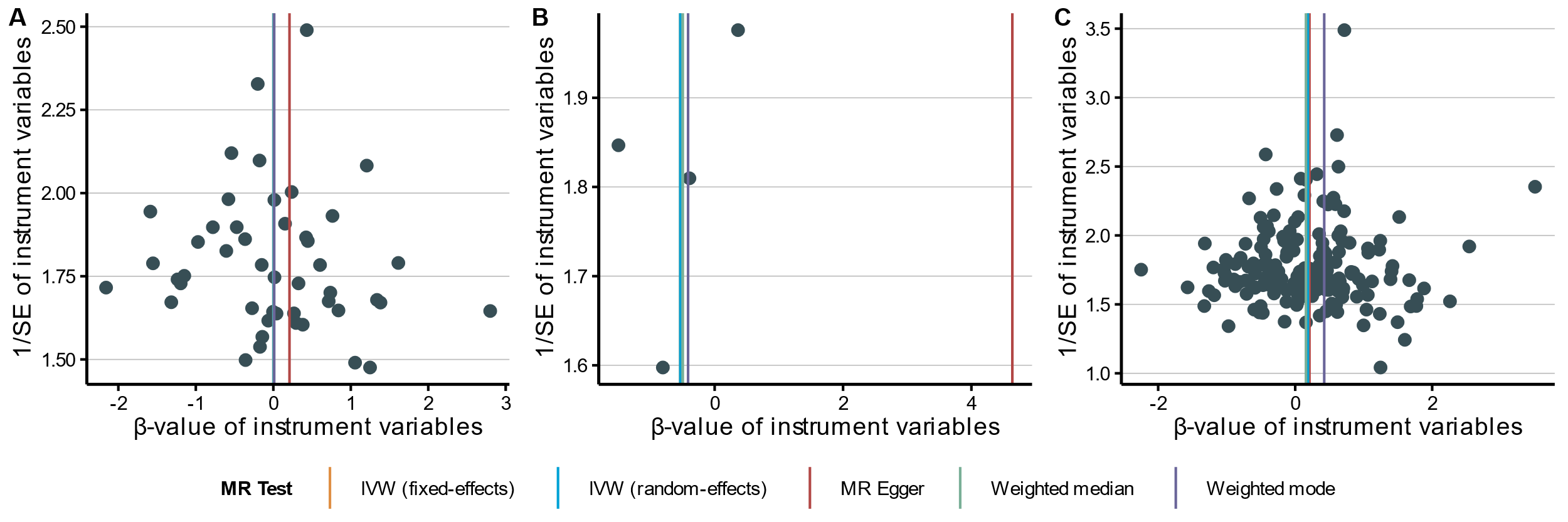


a. breast cancer (ER+) stands for estrogen receptor positive breast cancer.

Funnel plots with colored vertical lines representing total MR estimation of causal associations between (A) computer using and ER+ breast cancer, (B) driving and ER+ breast cancer, (C) television watching and ER+ breast cancer.

### eFigure 25. Scatter plots of leisure sedentary behaviors and breast cancer (ER+)^a^

^
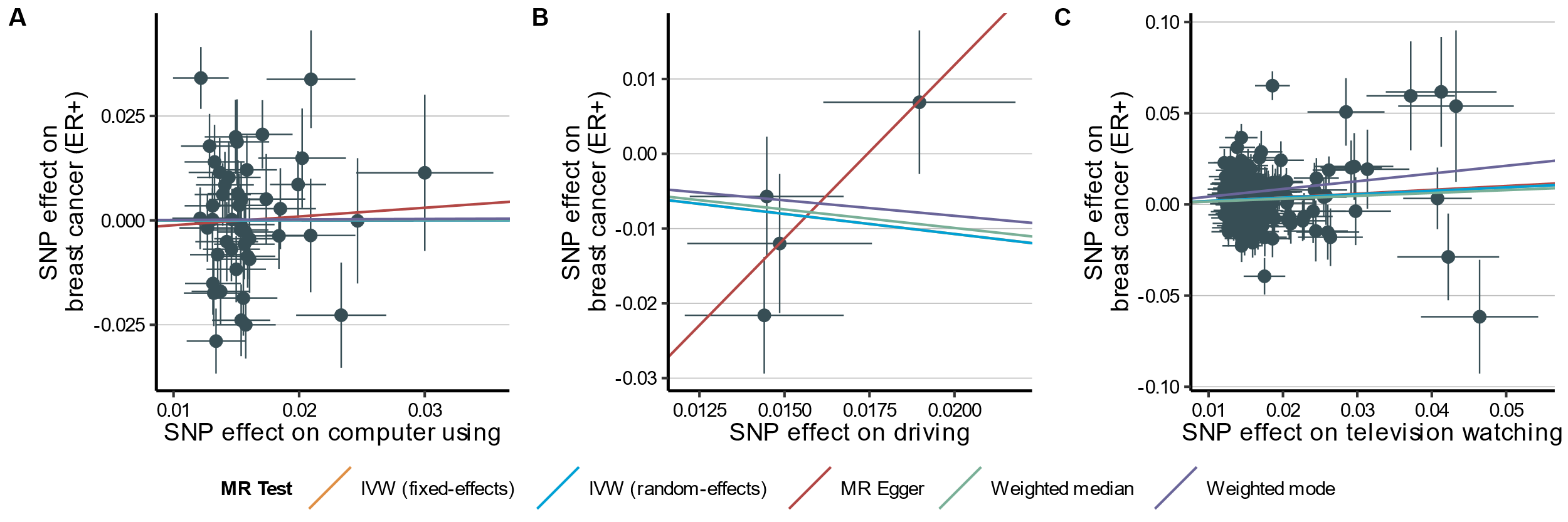
^

a. breast cancer (ER+) stands for estrogen receptor negative breast cancer.

Scatter plots with colored lines representing results of each mendelian randomization sensitivity analysis between (A) computer using and ER+ breast cancer, (B) driving and ER+ breast cancer, (C) television watching and ER+ breast cancer.

### eFigure 26. Leave-one-out plots of leisure sedentary behaviors and breast cancer (ER+)^a^


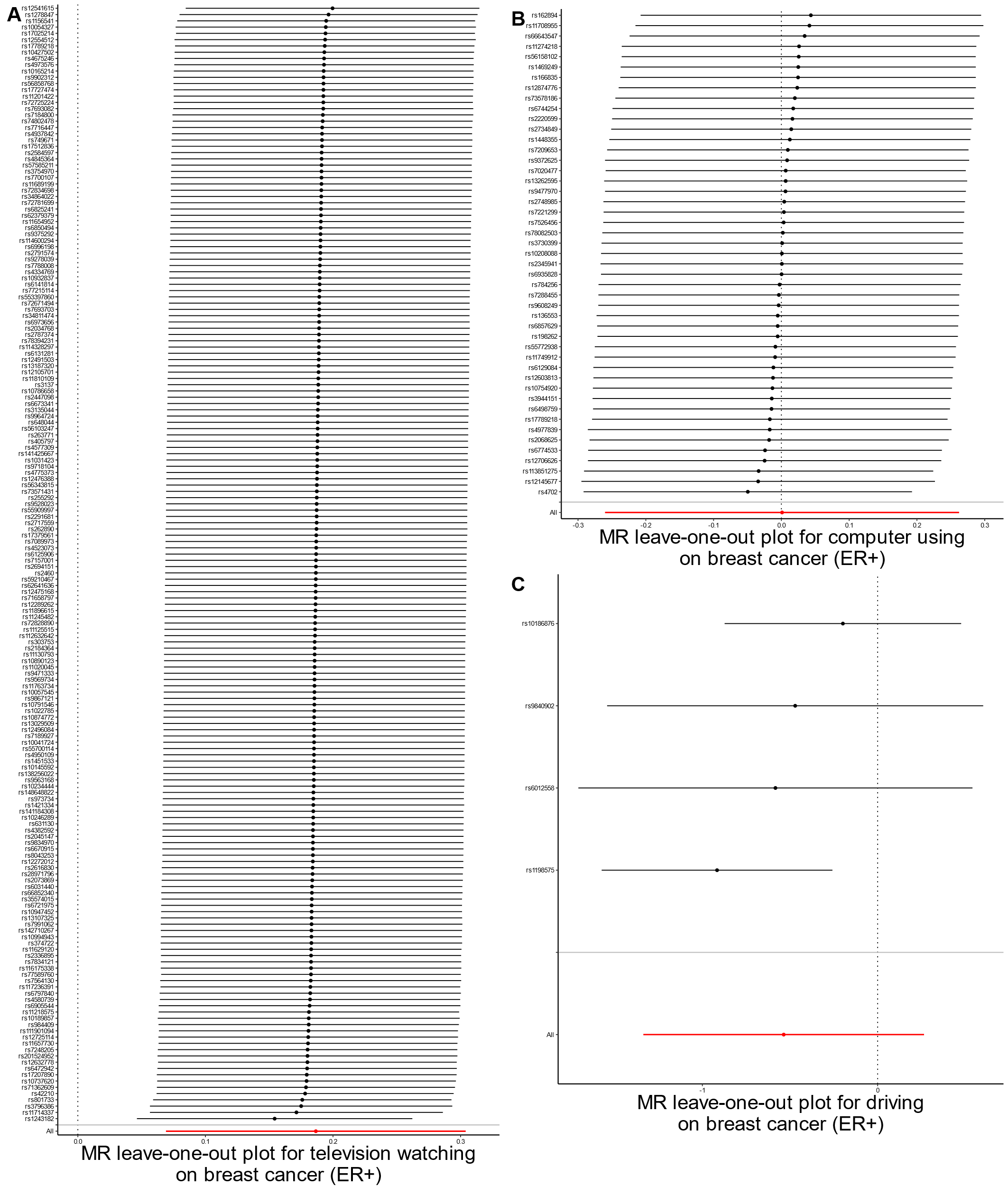


a. breast cancer (ER+) stands for estrogen receptor negative breast cancer.

Leave-one-out plot of Mendelian randomization sensitivity analysis between (A) television watching and ER+ breast cancer, (B) computer using and ER+ breast cancer, (C) driving and ER+ breast cancer.

### eFigure 27. Forest plots of single-SNP analysis of leisure sedentary behaviors and breast cancer (ER+)^a^


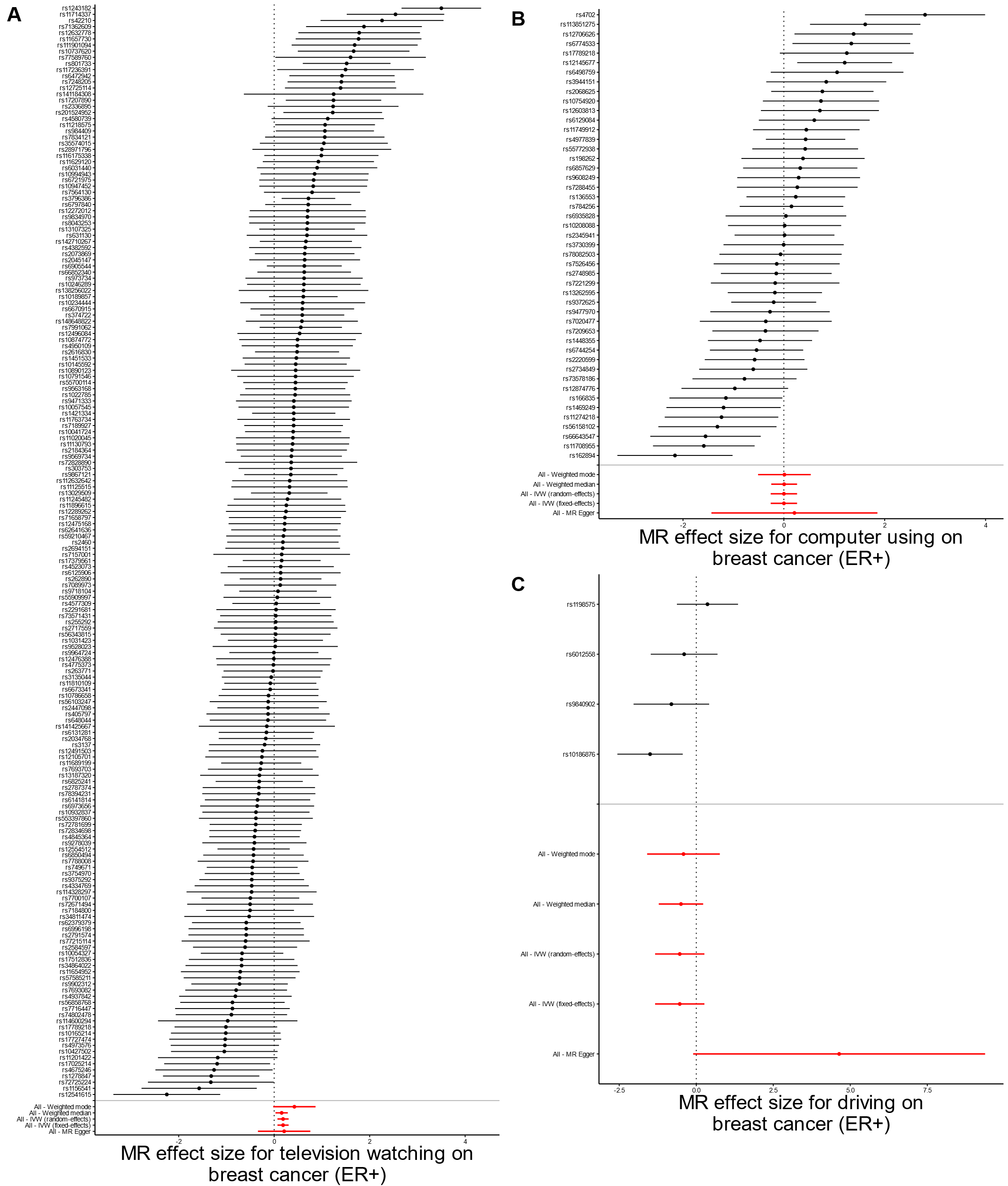


a. breast cancer (ER+) stands for estrogen receptor positive breast cancer.

The MR single-SNP analysis plots the Wald estimate of causal association between (A) television watching and ER+ breast cancer, (B) computer using and ER+ breast cancer, (C) driving and ER+ breast cancer.

## eFigures of ovarian cancer (low grade and low malignant potential serous subtype)

### eFigure 28. Funnel plots of leisure sedentary behaviors and ovarian cancer (LGS+LMPS)^a^


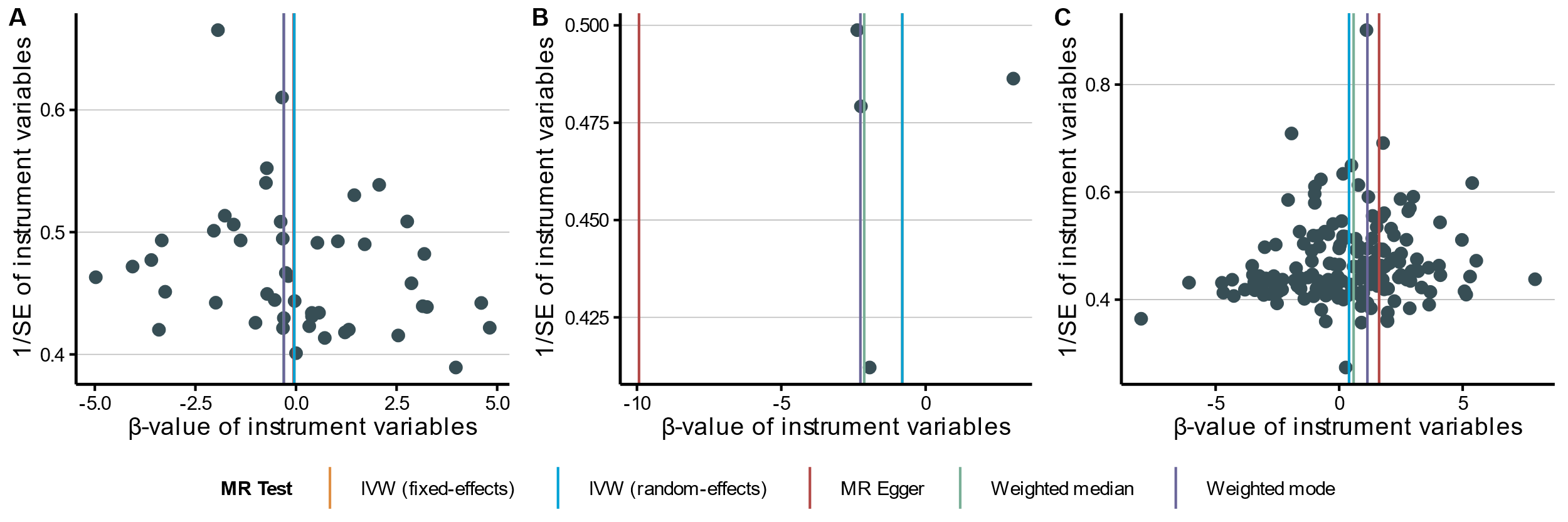


1. ovarian cancer (LGS+LMPS) stands for low grade and low malignant potential serous ovarian cancer.

Funnel plots with colored vertical lines representing total MR estimation of causal associations between (A) computer using and ovarian cancer (LGS+LMPS), (B) driving and ovarian cancer (LGS+LMPS), (C) television watching and ovarian cancer (LGS+LMPS).

### eFigure 29. Scatter plots of leisure sedentary behaviors and ovarian cancer (LGS+LMPS)^a^


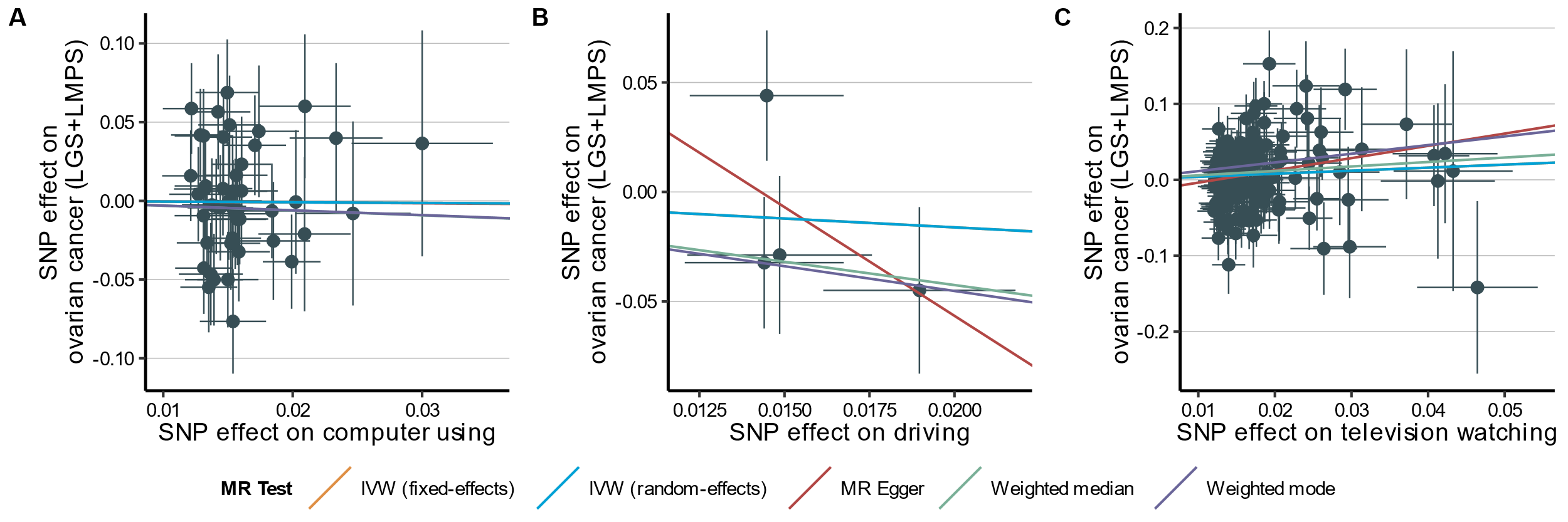


1. ovarian cancer (LGS+LMPS) stands for low grade and low malignant potential serous ovarian cancer.

Scatter plots with colored lines representing results of each mendelian randomization sensitivity analysis between (A) computer using and ovarian cancer (LGS+LMPS), (B) driving and ovarian cancer (LGS+LMPS), (C) television watching and ovarian cancer (LGS+LMPS).

### eFigure 30. Leave-one-out plots of leisure sedentary behaviors and ovarian cancer (LGS+LMPS)^a^


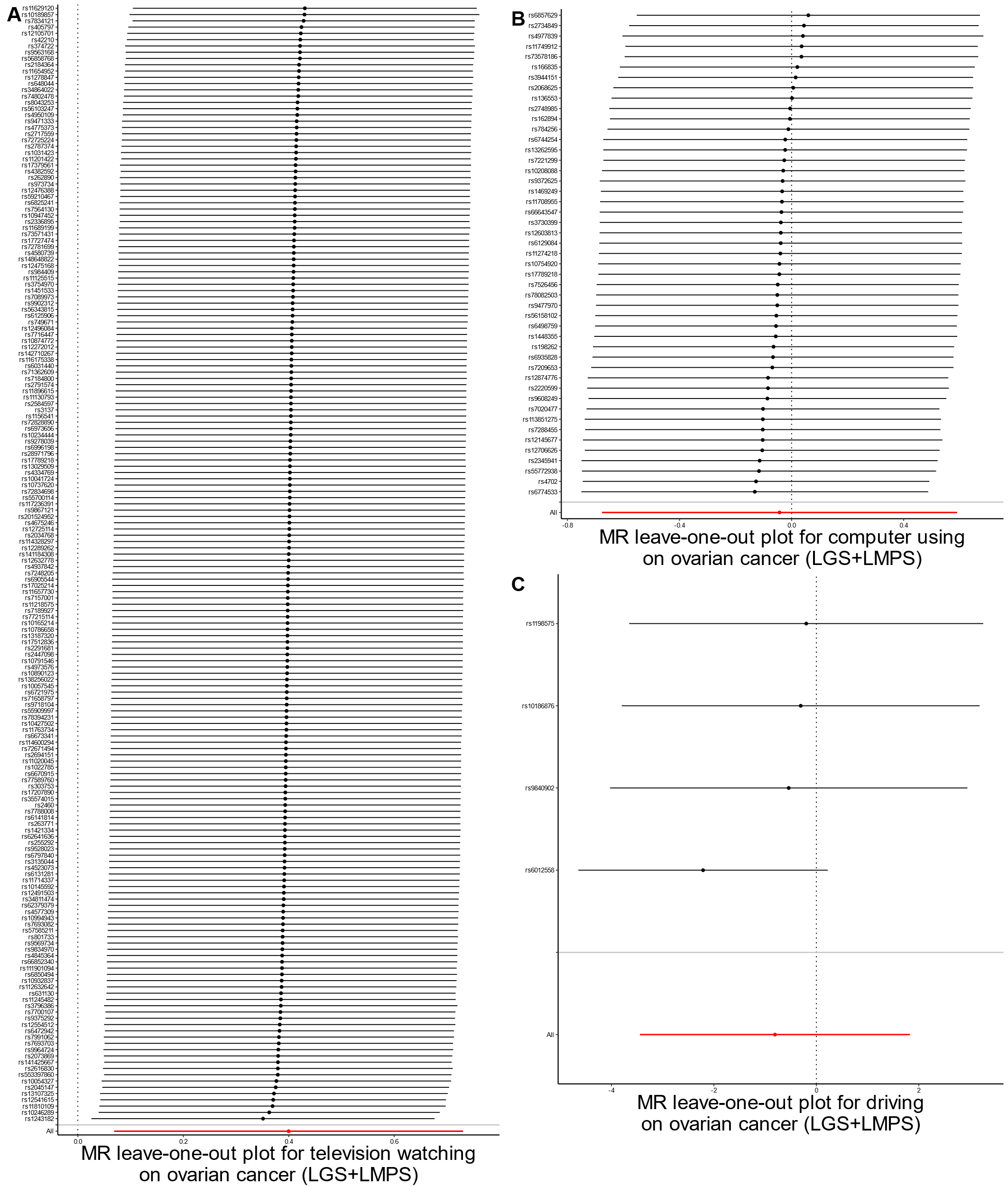


1. ovarian cancer (LGS+LMPS) stands for low grade and low malignant potential serous ovarian cancer.

Leave-one-out plot of Mendelian randomization sensitivity analysis between (A) television watching and ovarian cancer (LGS+LMPS), (B) computer using and ovarian cancer (LGS+LMPS), (C) driving and ovarian cancer (LGS+LMPS).

### eFigure 31. Forest plots of single-SNP analysis of leisure sedentary behaviors and ovarian cancer (LGS+LMPS)^a^


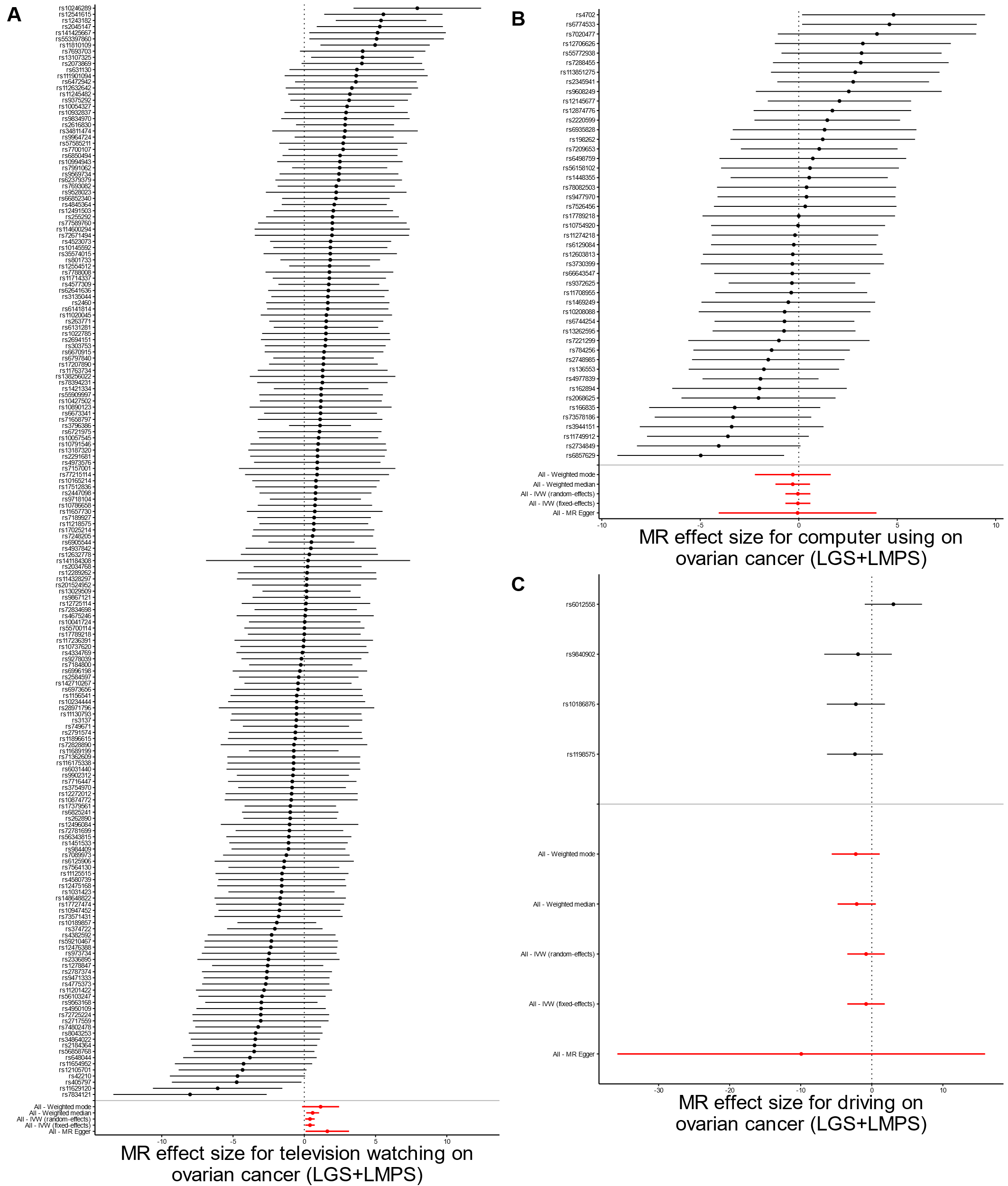


a. ovarian cancer (LGS+LMPS) stands for low grade and low malignant potential serous ovarian cancer

The MR single-SNP analysis plots the Wald estimate of causal association between (A) television watching and ovarian cancer (LGS+LMPS), (B) computer using and ovarian cancer (LGS+LMPS), (C) driving and ovarian cancer (LGS+LMPS).

## eFigures of ovarian cancer (overall)

### eFigure 32. Funnel plots of leisure sedentary behaviors and ovarian cancer (overall)^a^

^
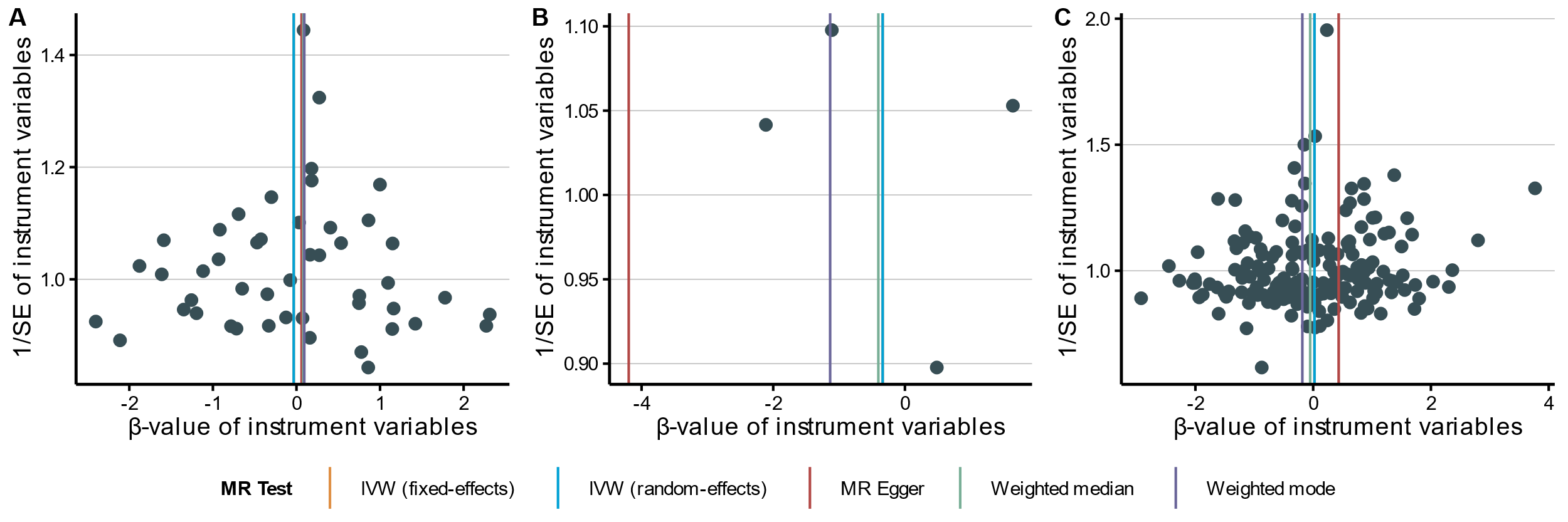
^

1. ovarian cancer (overall) stands for ovarian cancer including low grade and low malignant potential serous ovarian cancer, high grade serous ovarian cancer, clear cell ovarian cancer, endometrioid ovarian cancer, mucinous ovarian cancer.

Funnel plots with colored vertical lines representing total MR estimation of causal associations between (A) computer using and overall ovarian cancer, (B) driving and overall ovarian cancer, (C) television watching and overall ovarian cancer.

### eFigure 33. Scatter plots of leisure sedentary behaviors and ovarian cancer (overall)^a^


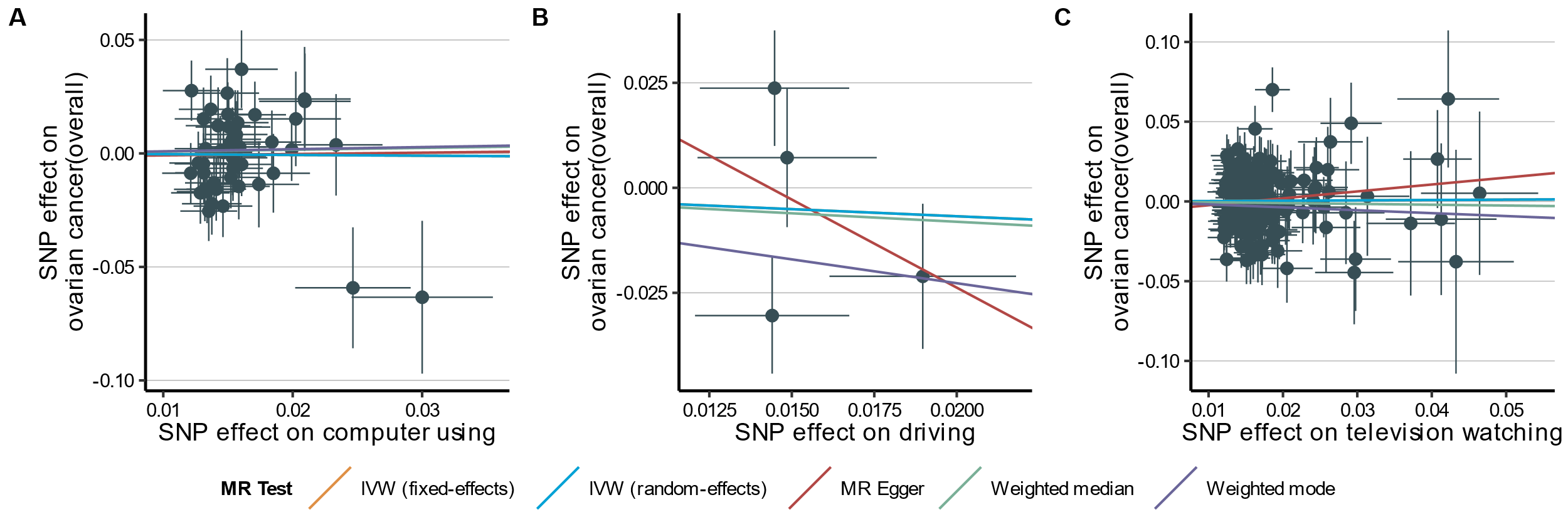


1. ovarian cancer (overall) stands for breast cancer including low grade and low malignant potential serous ovarian cancer, high grade serous ovarian cancer, clear cell ovarian cancer, endometrioid ovarian cancer, mucinous ovarian cancer.

Scatter plots with colored lines representing results of each mendelian randomization sensitivity analysis between (A) computer using and overall ovarian cancer, (B) driving and overall ovarian cancer, (C) television watching and overall ovarian cancer.

### eFigure 34. Leave-one-out plots of leisure sedentary behaviors and ovarian cancer (overall)^a^


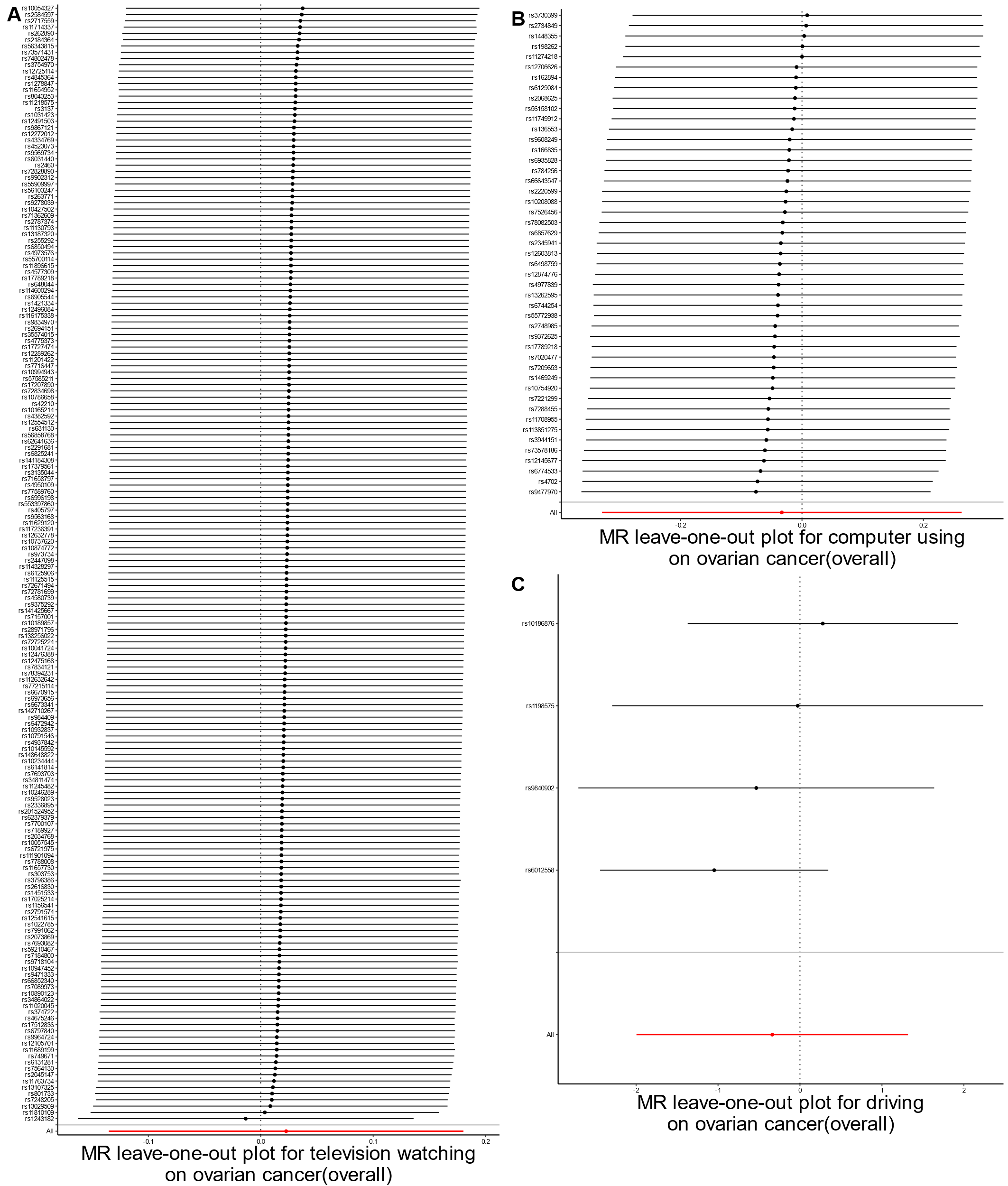


1. ovarian cancer (overall) stands for ovarian cancer including low grade and low malignant potential serous ovarian cancer, high grade serous ovarian cancer, clear cell ovarian cancer, endometrioid ovarian cancer, mucinous ovarian cancer.

Leave-one-out plot of Mendelian randomization sensitivity analysis between (A) television watching and overall endometrial cancer, (B) computer using and overall endometrial cancer, (C) driving and overall endometrial cancer.

### eFigure 35. Forest plots of single-SNP analysis of leisure sedentary behaviors and ovarian cancer (overall)^a^


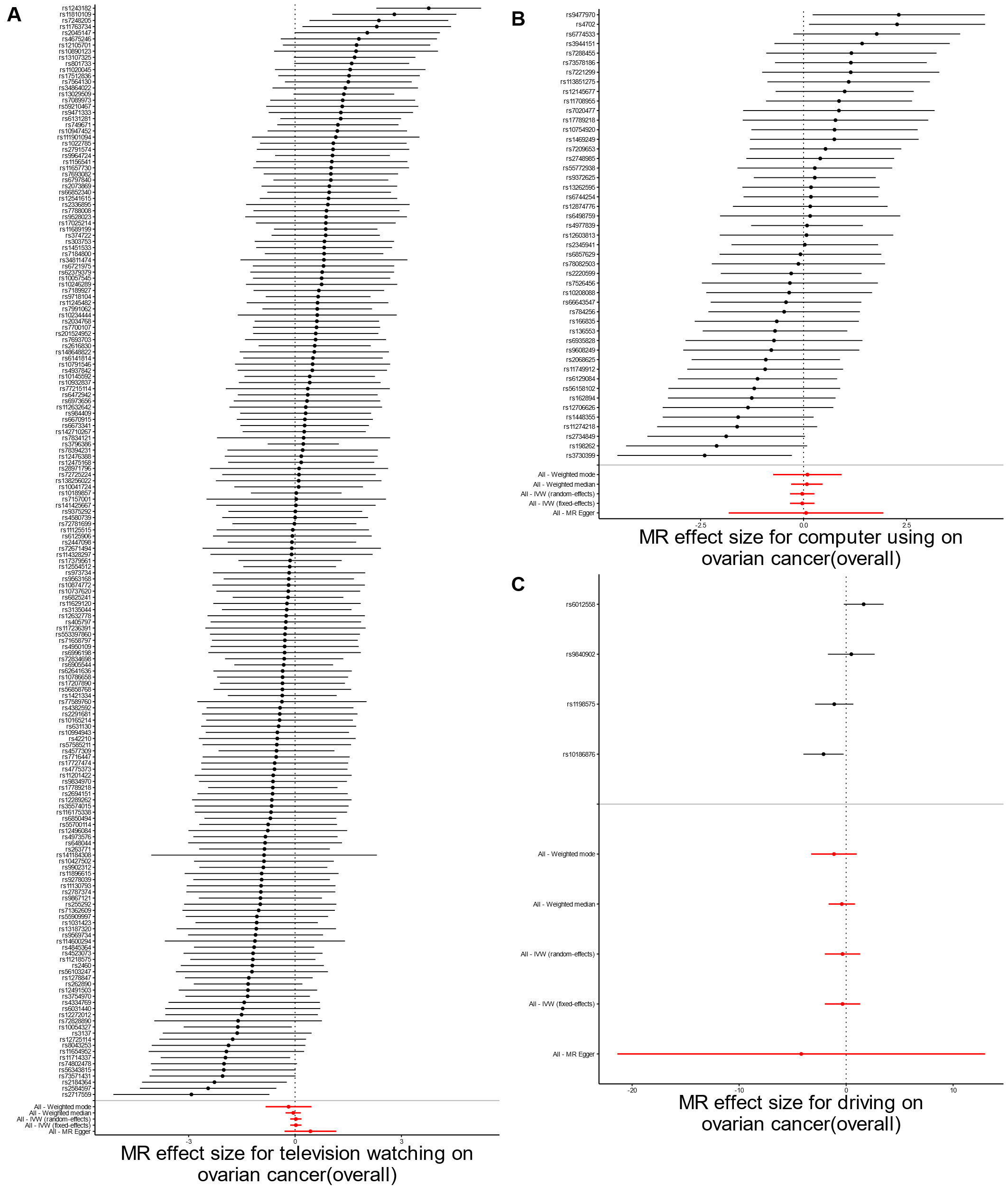


a. ovarian cancer (overall) stands for ovarian cancer including low grade and low malignant potential serous ovarian cancer, high grade serous ovarian cancer, clear cell ovarian cancer endometrioid ovarian cancer and mucinous ovarian cancer

The MR single-SNP analysis plots the Wald estimate of causal association between (A) television watching and overall ovarian cancer, (B) computer using and overall ovarian cancer, (C) driving and overall ovarian cancer.

## eFigures of ovarian cancer (high grade serous subtype)

### eFigure 36. Funnel plots of leisure sedentary behaviors and high grade serous ovarian cancer


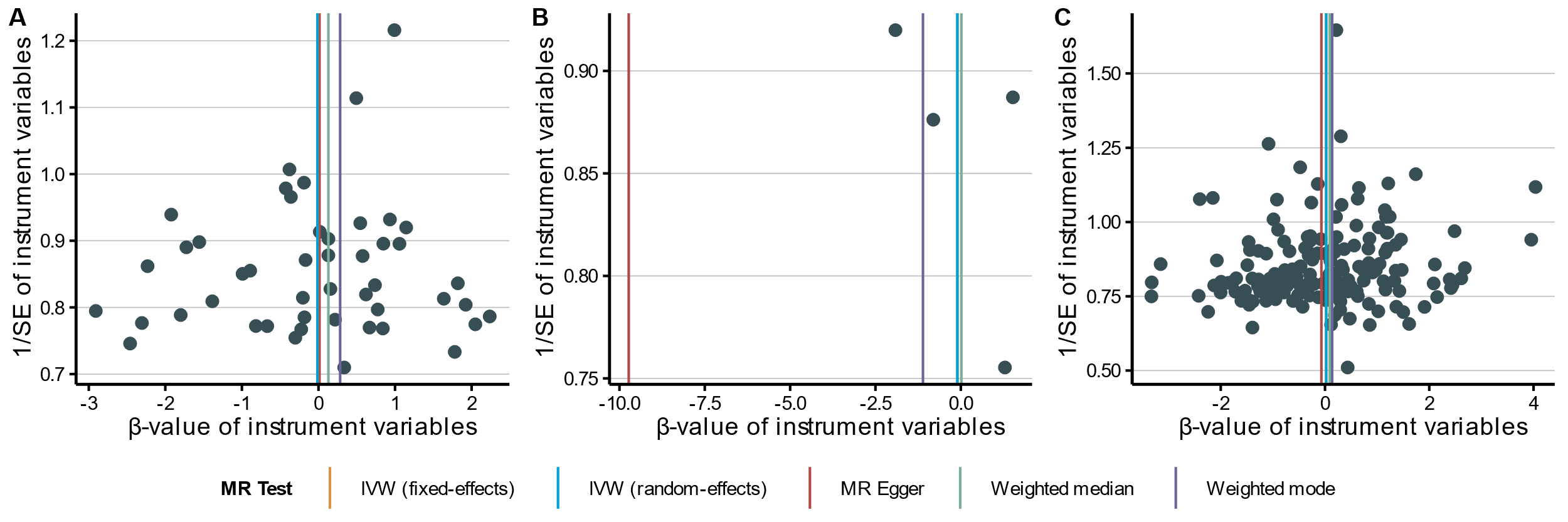


Funnel plots with colored vertical lines representing total MR estimation of causal associations between (A) computer using and high grade serous ovarian cancer, (B) driving and high grade serous ovarian cancer, (C) television watching and high grade serous ovarian cancer.

### eFigure 37. Scatter plots of leisure sedentary behaviors and high grade serous ovarian cancer


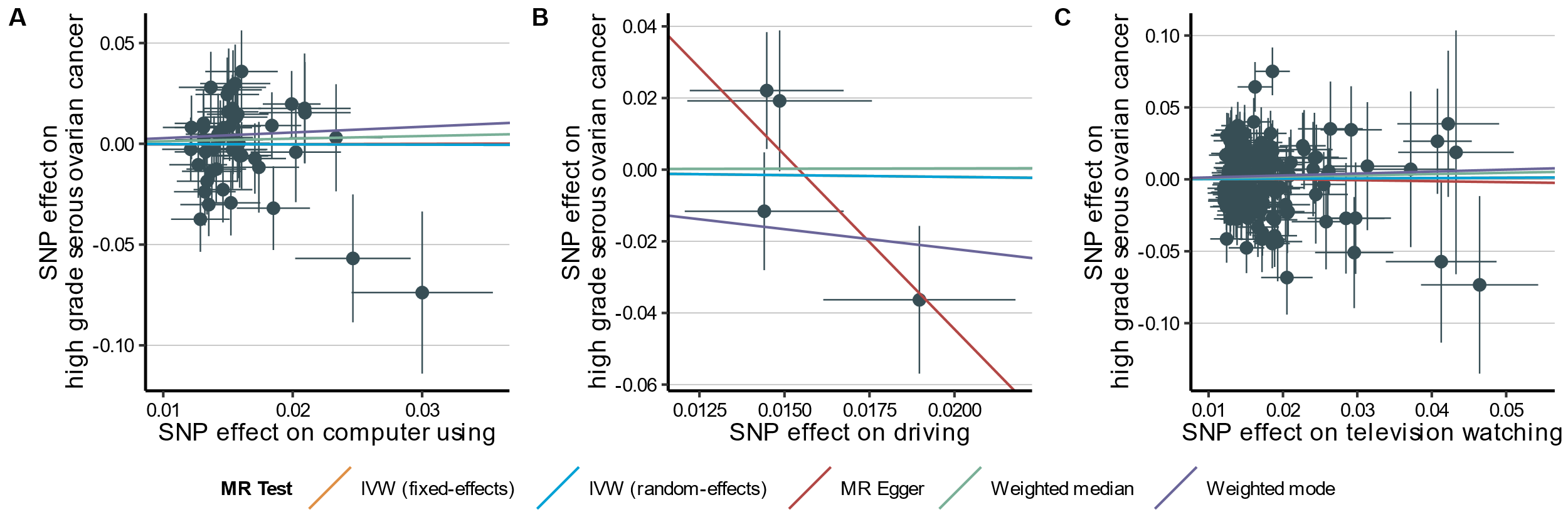


Scatter plots with colored lines representing results of each mendelian randomization sensitivity analysis between (A) computer using and high grade serous ovarian cancer, (B) driving and high grade serous ovarian cancer, (C) television watching and high grade serous ovarian cancer.

### eFigure 38. Leave-one-out plots of leisure sedentary behaviors and high grade serous ovarian cancer


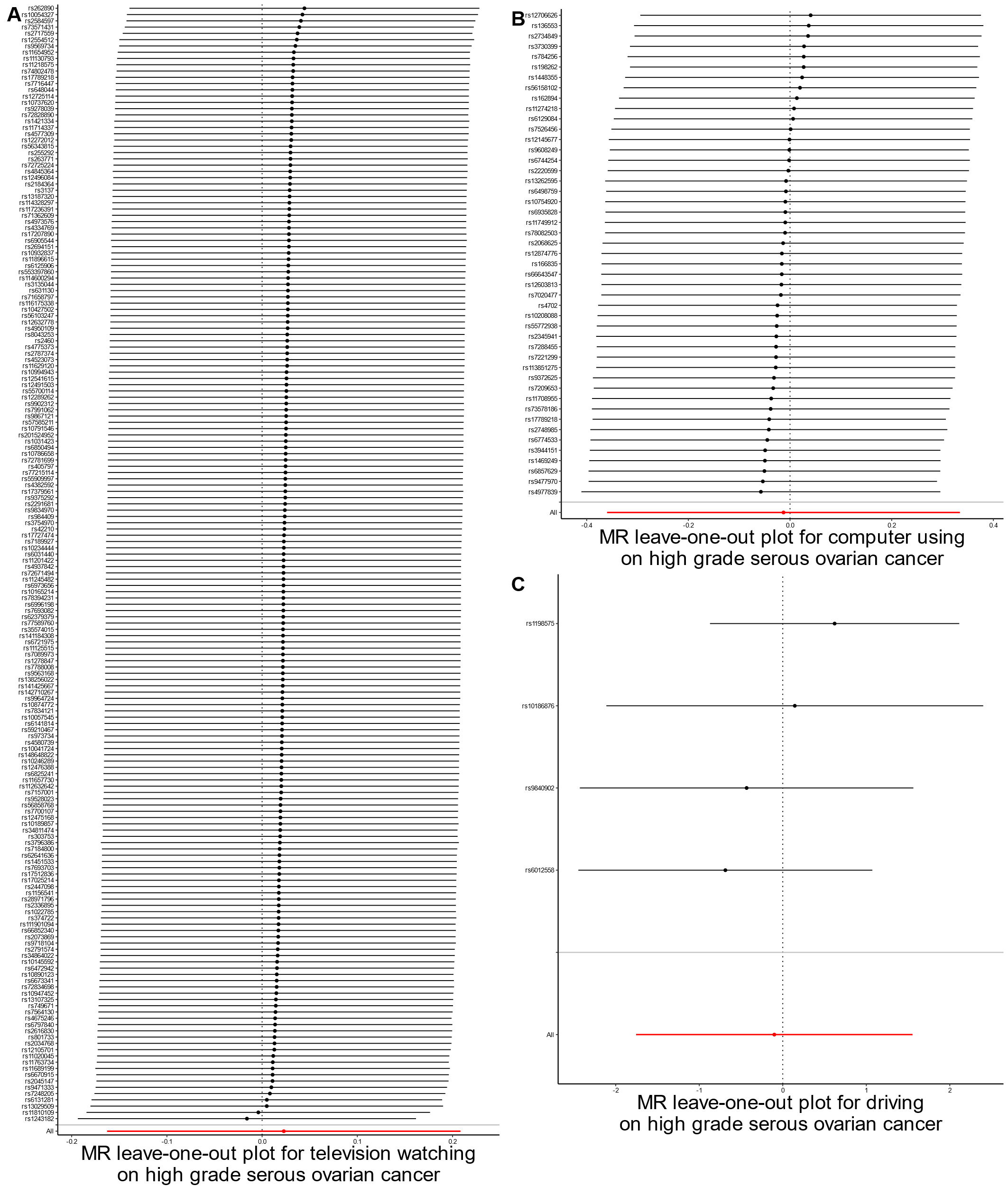


Leave-one-out plot of Mendelian randomization sensitivity analysis between (A) television watching and high grade serous ovarian cancer, (B) computer using and high grade serous ovarian cancer, (C) driving and high grade serous ovarian cancer.

### eFigure 39. Forest plots of single-SNP analysis of leisure sedentary behaviors and high grade serous ovarian cancer


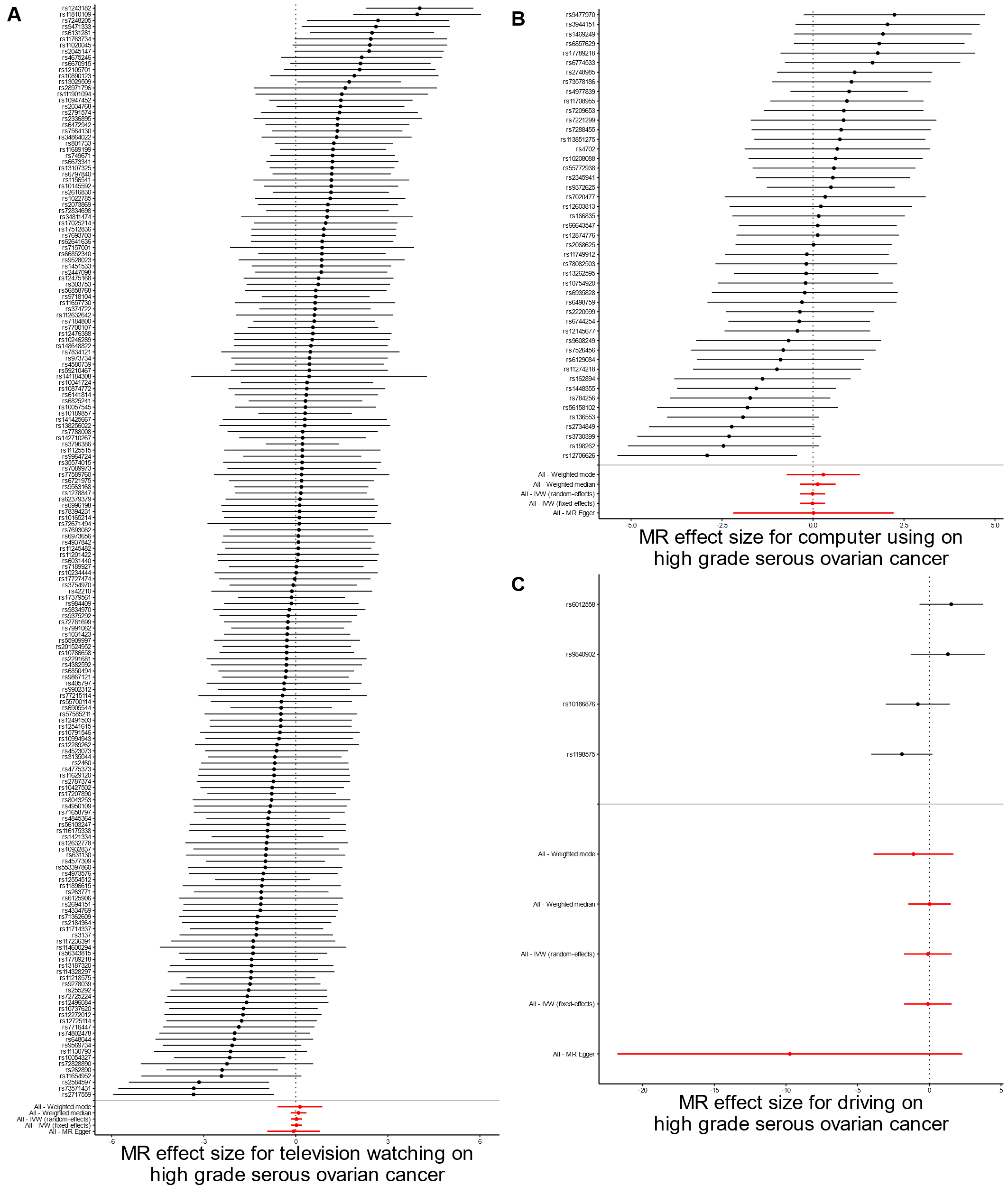


The MR single-SNP analysis plots the Wald estimate of causal association between (A) television watching and high grade serous ovarian cancer, (B) computer using and high grade serous ovarian cancer, (C) driving and high grade serous ovarian cancer.

## eFigures of ovarian cancer (clear cell subtype)

### eFigure 40. Funnel plots of leisure sedentary behaviors and clear cell ovarian cancer


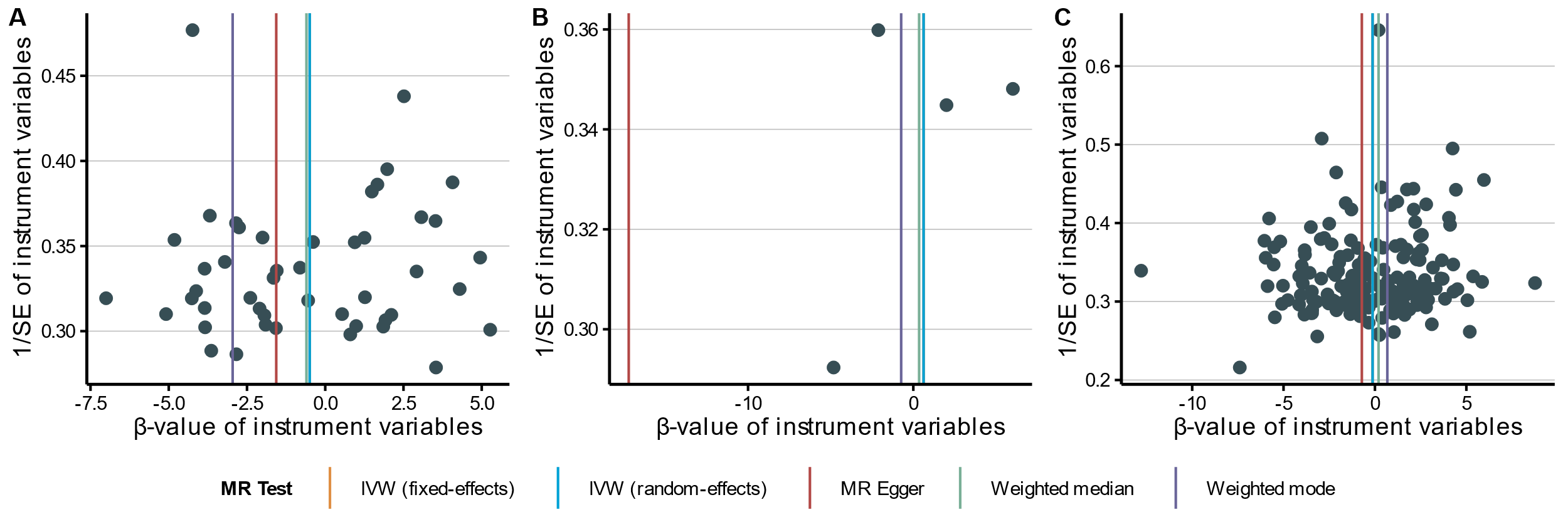


Funnel plots with colored vertical lines representing total MR estimation of causal associations between (A) computer using and clear cell ovarian cancer, (B) driving and clear cell ovarian cancer, (C) television watching and clear cell ovarian cancer.

### eFigure 41. Scatter plots of leisure sedentary behaviors and clear cell ovarian cancer


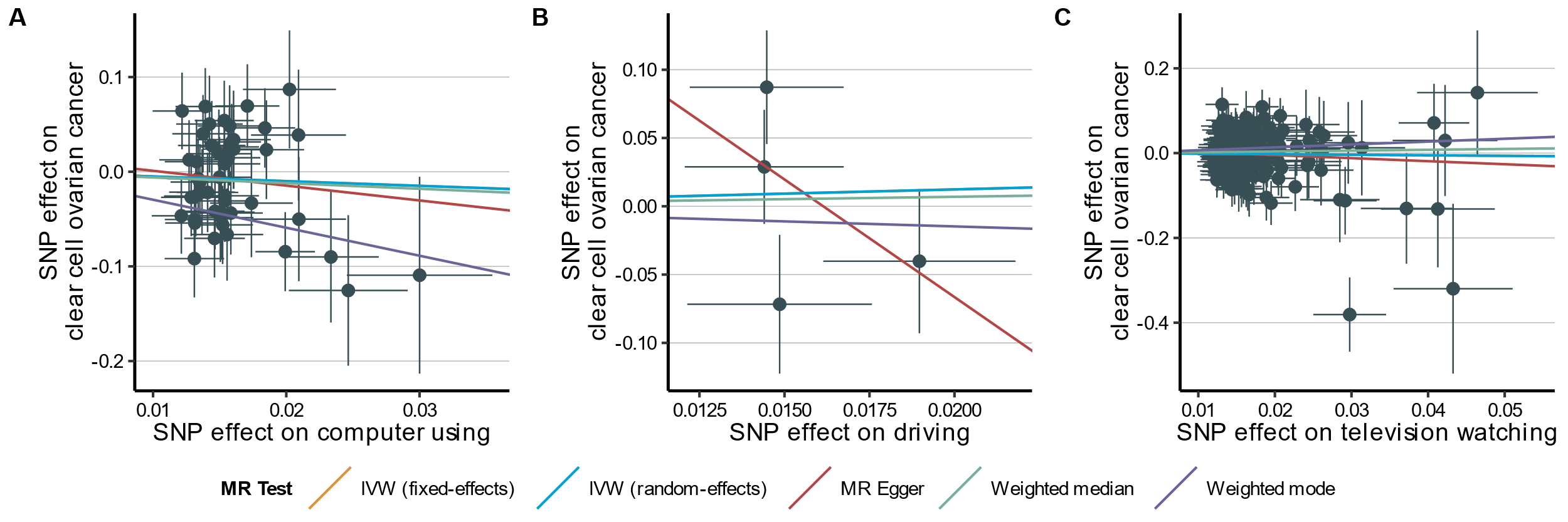


Scatter plots with colored lines representing results of each mendelian randomization sensitivity analysis between (A) computer using and clear cell ovarian cancer, (B) driving and clear cell ovarian cancer, (C) television watching and clear cell ovarian cancer.

### eFigure 42. Leave-one-out plots of leisure sedentary behaviors and clear cell ovarian cancer


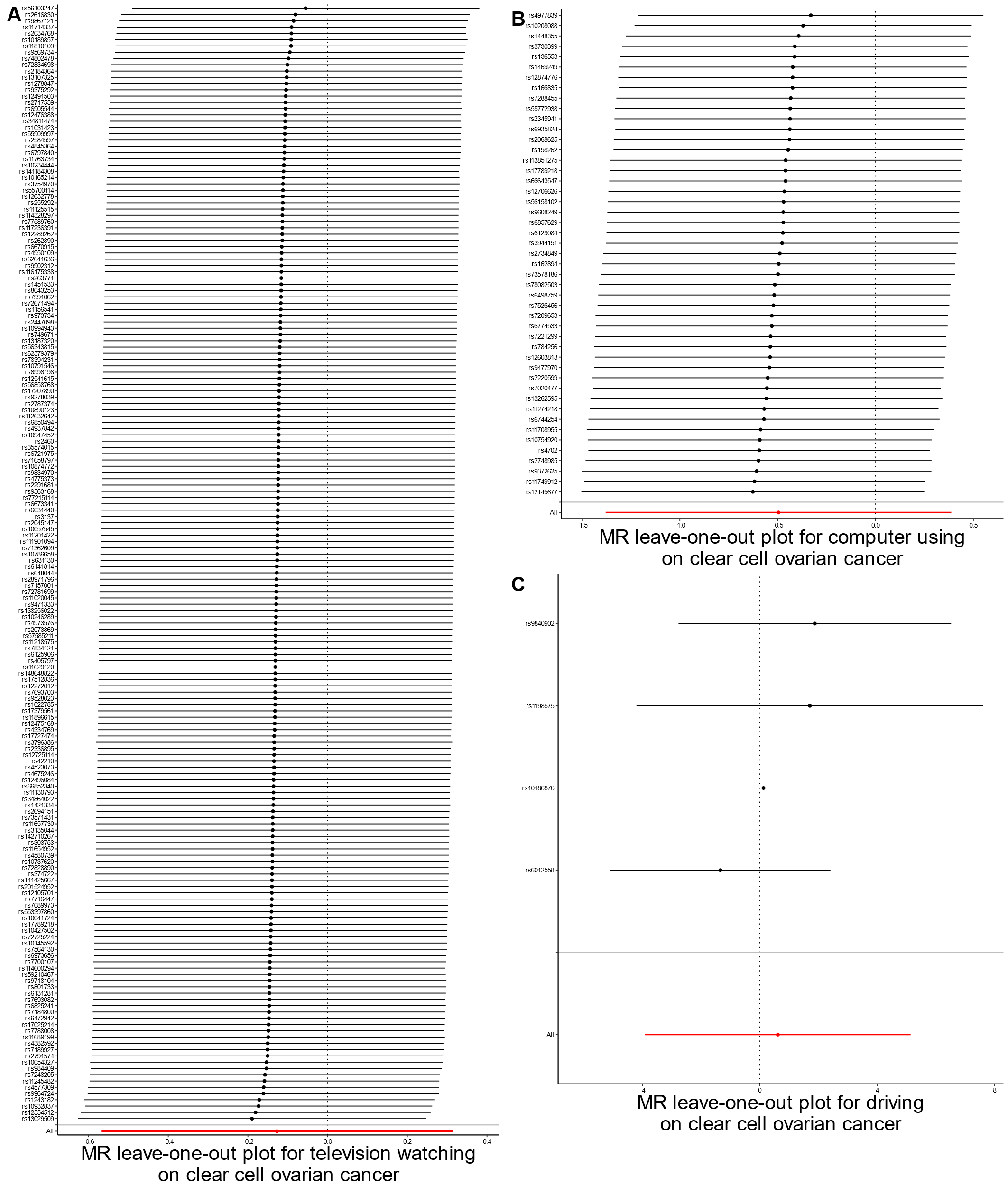


Leave-one-out plot of Mendelian randomization sensitivity analysis between (A) television watching and clear cell ovarian cancer, (B) computer using and clear cell ovarian cancer, (C) driving and clear cell ovarian cancer.

### eFigure 43. Forest plots of single-SNP analysis of leisure sedentary behaviors and clear cell ovarian cancer


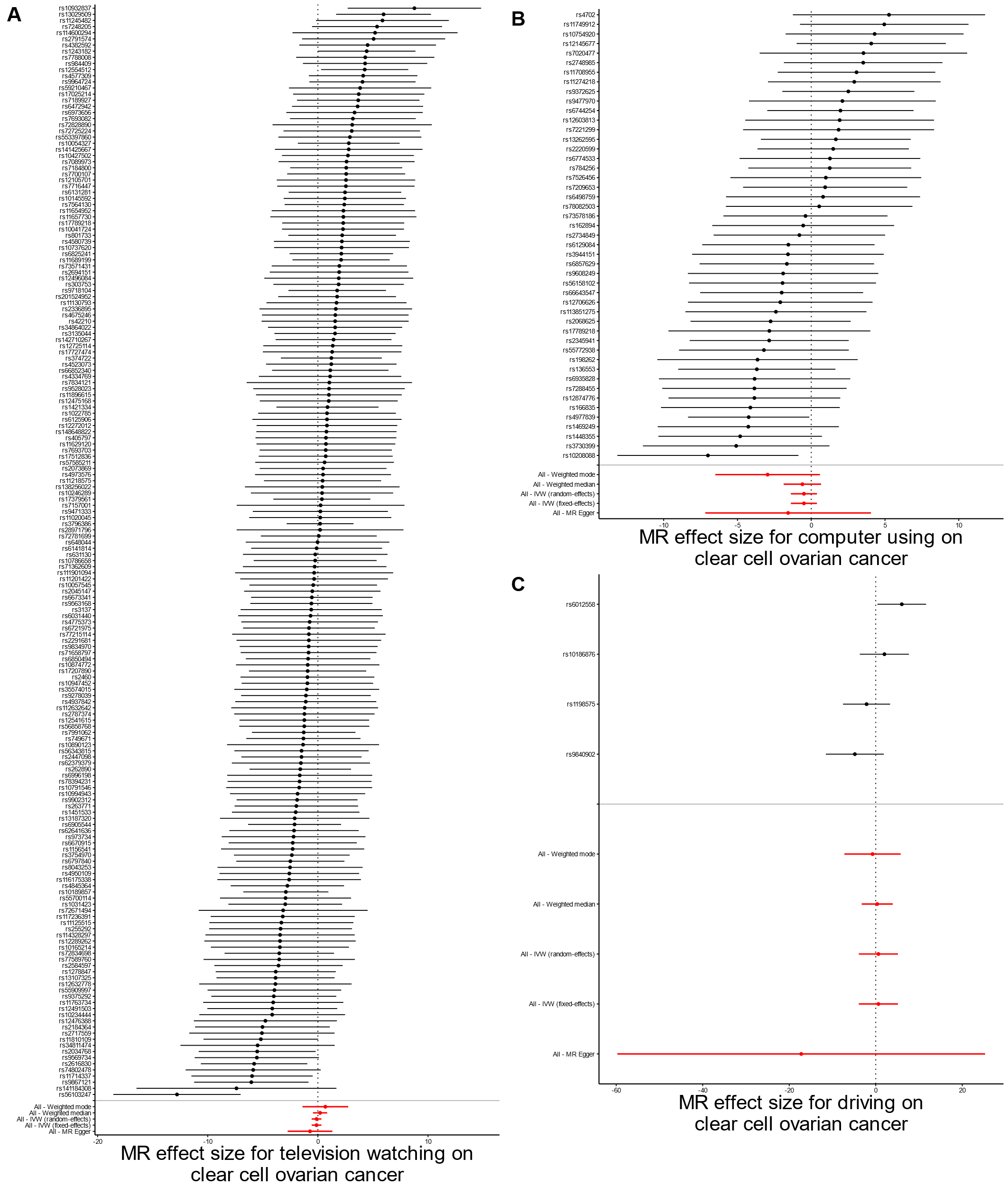


The MR single-SNP analysis plots the Wald estimate of causal association between (A) television watching and clear cell ovarian cancer, (B) computer using and clear cell ovarian cancer, (C) driving and clear cell ovarian cancer.

## eFigures of ovarian cancer (endometrioid subtype)

### eFigure 44. Funnel plots of leisure sedentary behaviors and endometrioid ovarian cancer


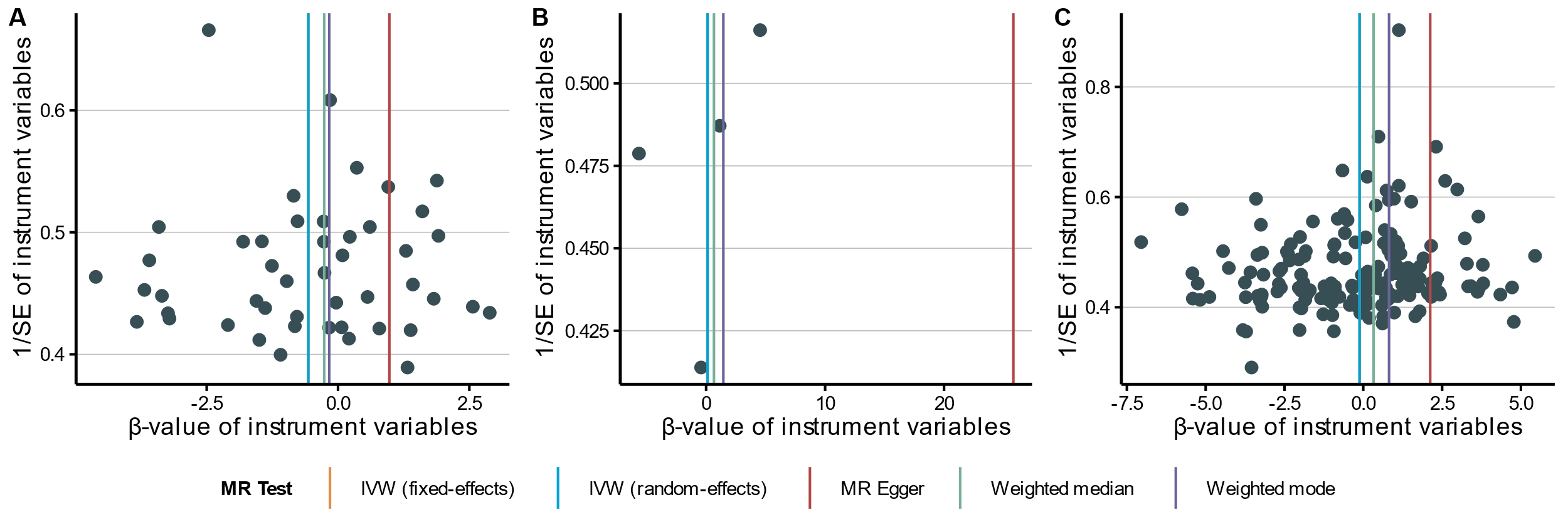


Funnel plots with colored vertical lines representing total MR estimation of causal associations between (A) computer using and endometrioid ovarian cancer, (B) driving and endometrioid ovarian cancer, (C) television watching and endometrioid ovarian cancer.

### eFigure 45. Scatter plots of leisure sedentary behaviors and endometrioid ovarian cancer


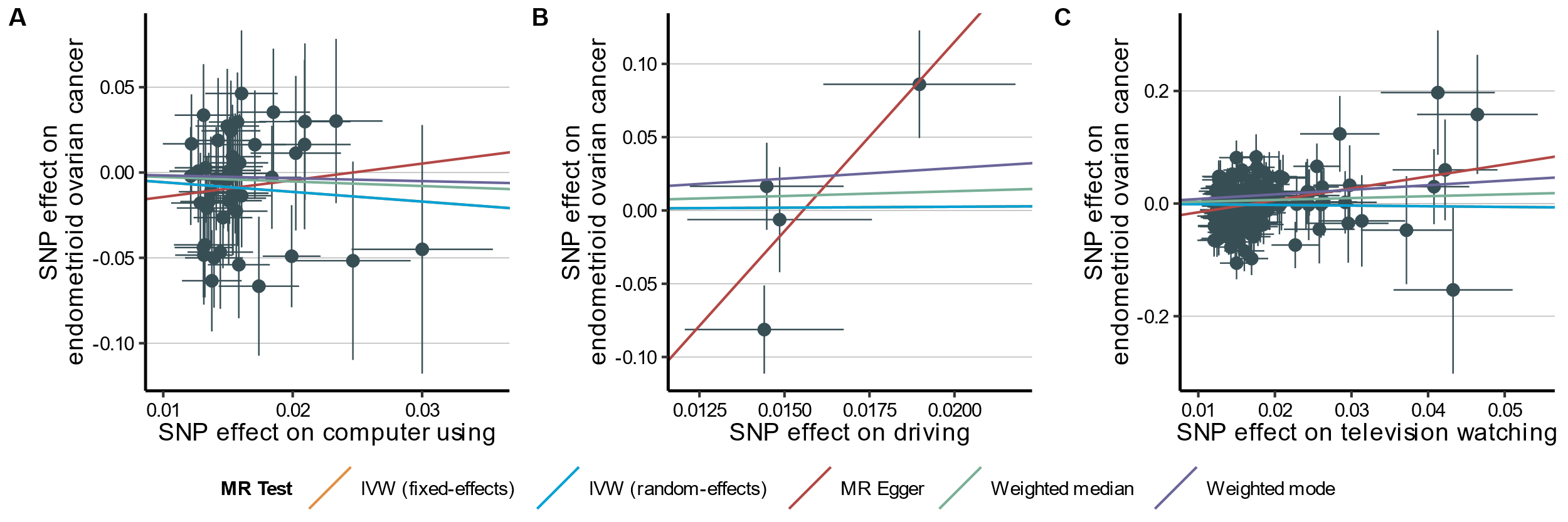


Scatter plots with colored lines representing results of each mendelian randomization sensitivity analysis between (A) computer using and endometrioid ovarian cancer, (B) driving and endometrioid ovarian cancer, (C) television watching and endometrioid ovarian cancer.

### eFigure 46. Leave-one-out plots of leisure sedentary behaviors and endometrioid ovarian cancer


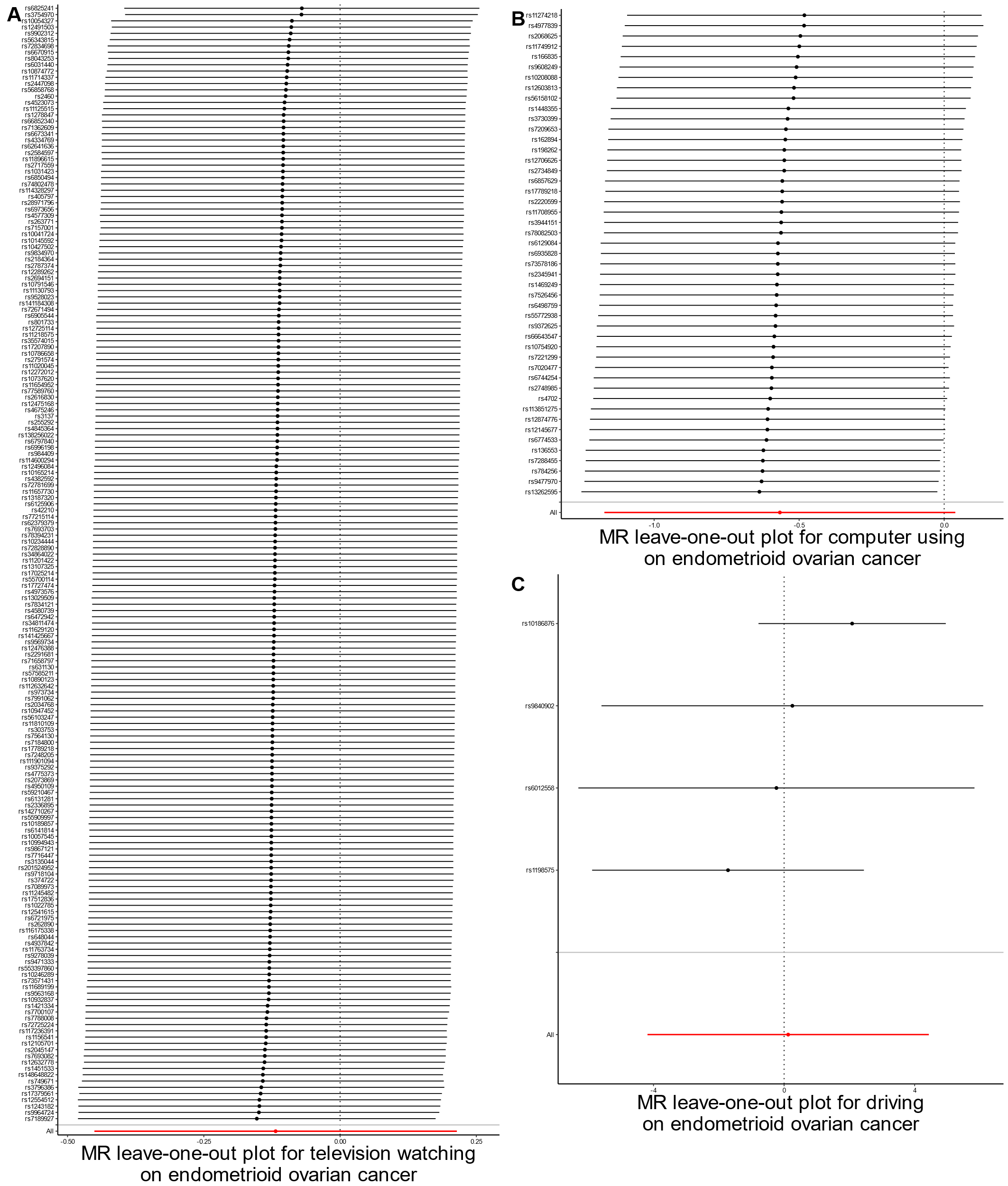


Leave-one-out plot of Mendelian randomization sensitivity analysis between (A) television watching and endometrioid ovarian cancer, (B) computer using and endometrioid ovarian cancer, (C) driving and endometrioid ovarian cancer.

### eFigure 47. Forest plots of single-SNP analysis of leisure sedentary behaviors and endometrioid ovarian cancer


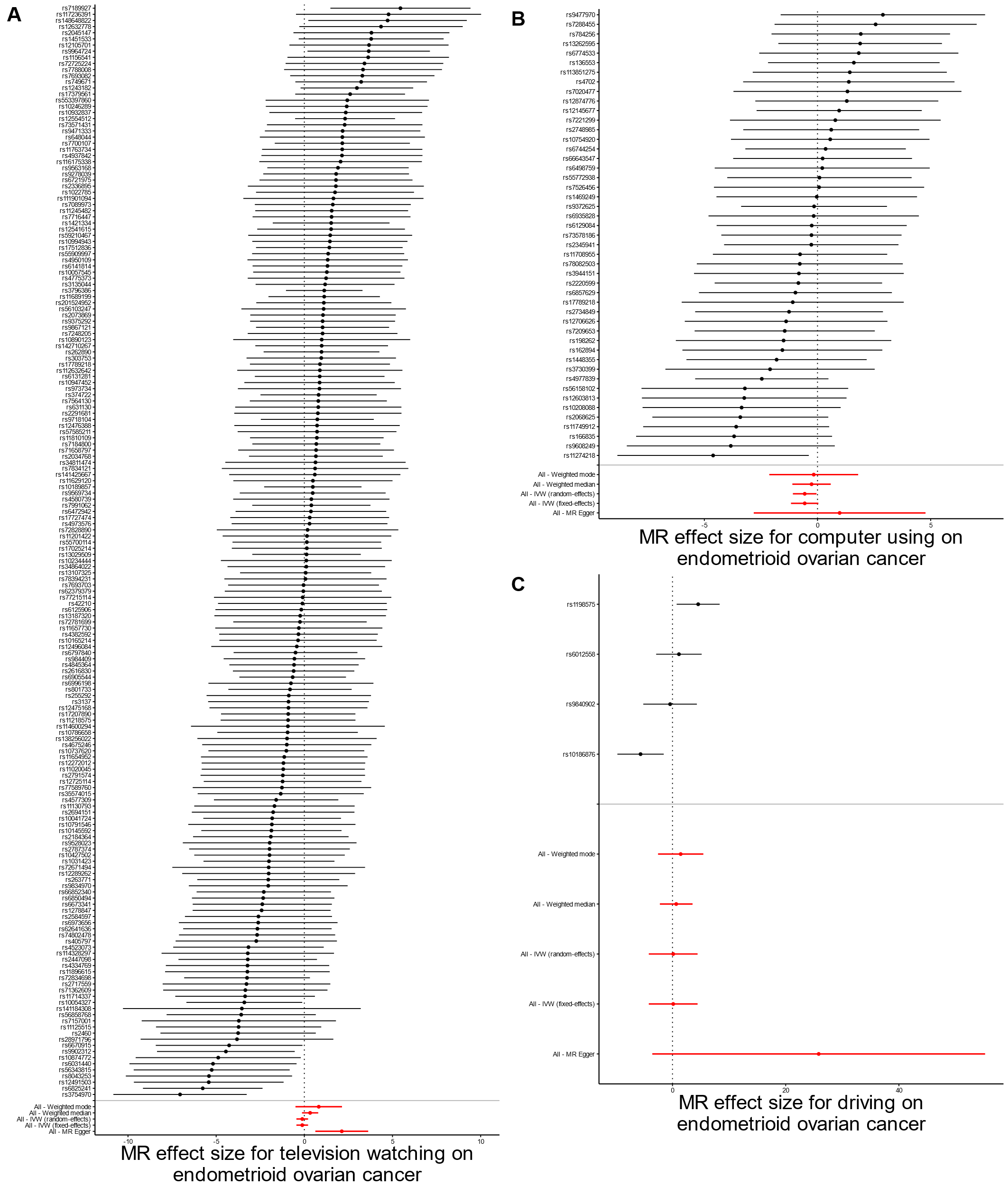


The MR single-SNP analysis plots the Wald estimate of causal association between (A) television watching and endometrioid ovarian cancer, (B) computer using and endometrioid ovarian cancer, (C) driving and endometrioid ovarian cancer.

## eFigures of ovarian cancer (mucinous subtype)

### eFigure 48. Scatter plots of leisure sedentary behaviors and mucinous ovarian cancer


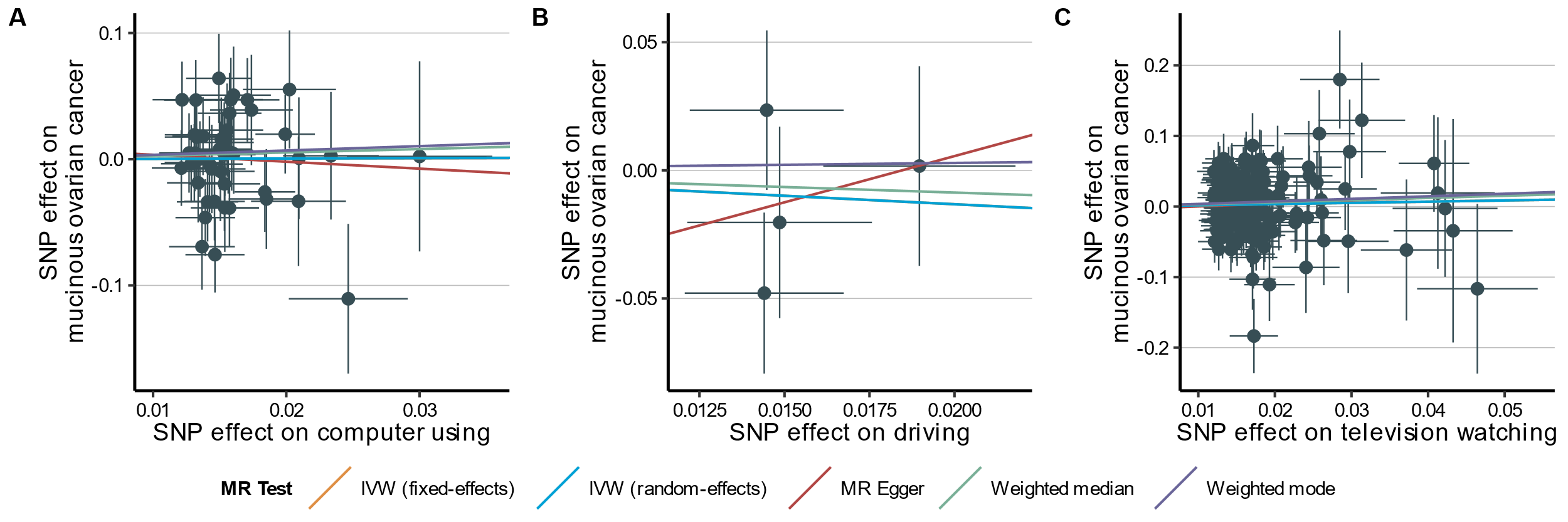


Scatter plots with colored lines representing results of each mendelian randomization sensitivity analysis between (A) computer using and mucinous ovarian cancer, (B) driving and mucinous ovarian cancer, (C) television watching and mucinous ovarian cancer.

### eFigure 49. Funnel plots of leisure sedentary behaviors and mucinous ovarian cancer


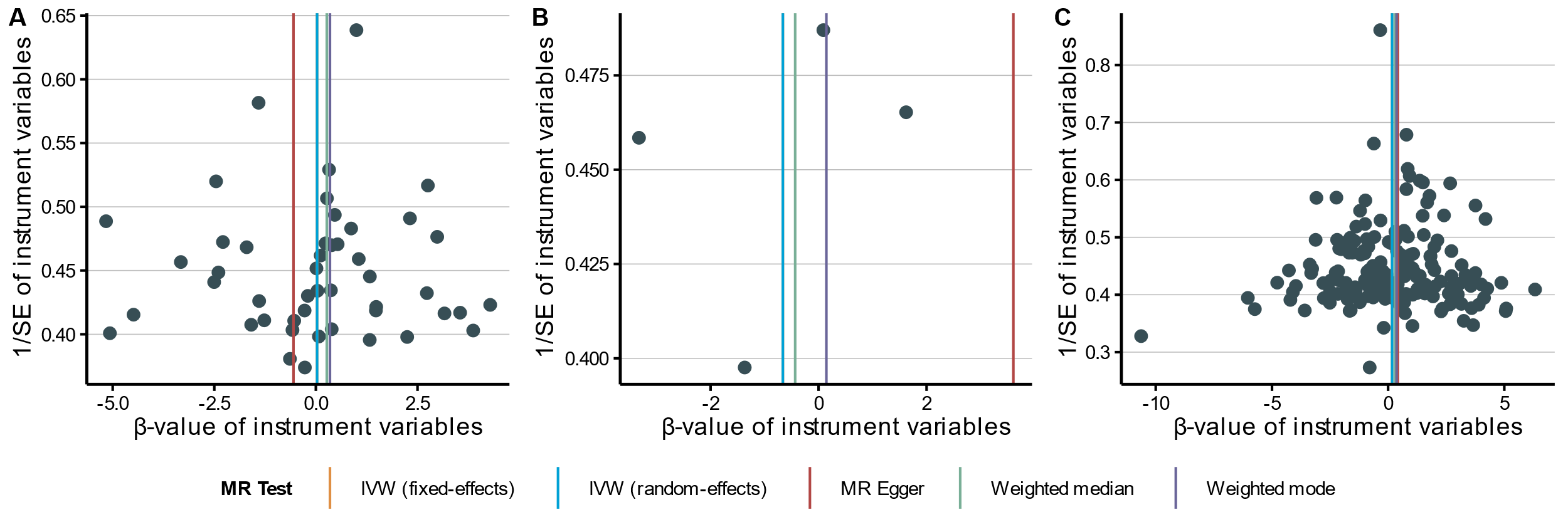


Funnel plots with colored vertical lines representing total MR estimation of causal associations between (A) computer using and mucinous ovarian cancer, (B) driving and mucinous ovarian cancer, (C) television watching and mucinous ovarian cancer.

### eFigure 50. Leave-one-out plots of leisure sedentary behaviors and mucinous ovarian cancer


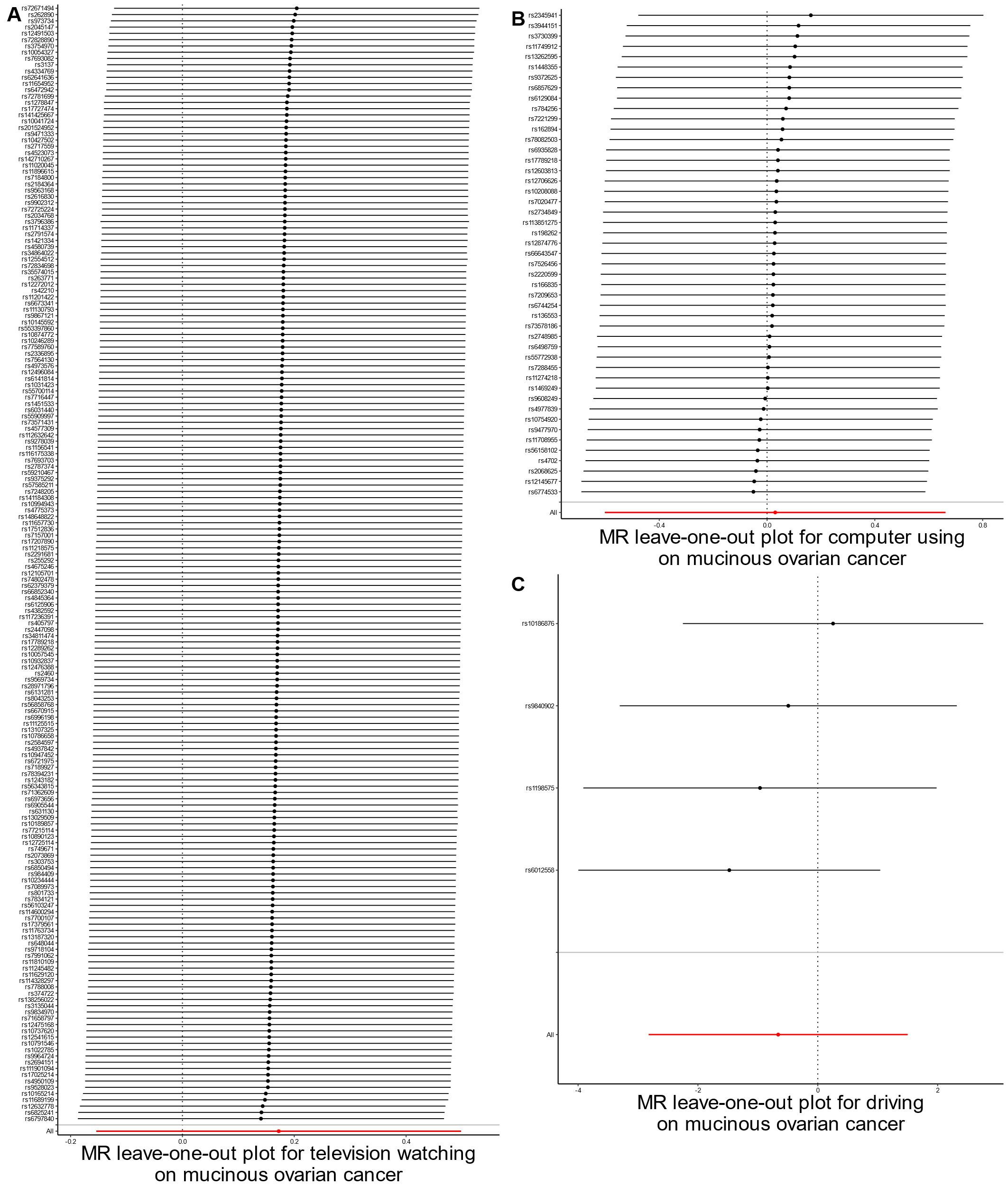


Leave-one-out plot of Mendelian randomization sensitivity analysis between (A) television watching and mucinous ovarian cancer, (B) computer using and mucinous ovarian cancer, (C) driving and mucinous ovarian cancer.

### eFigure 51. Forest plots of single-SNP analysis of leisure sedentary behaviors and mucinous ovarian cancer


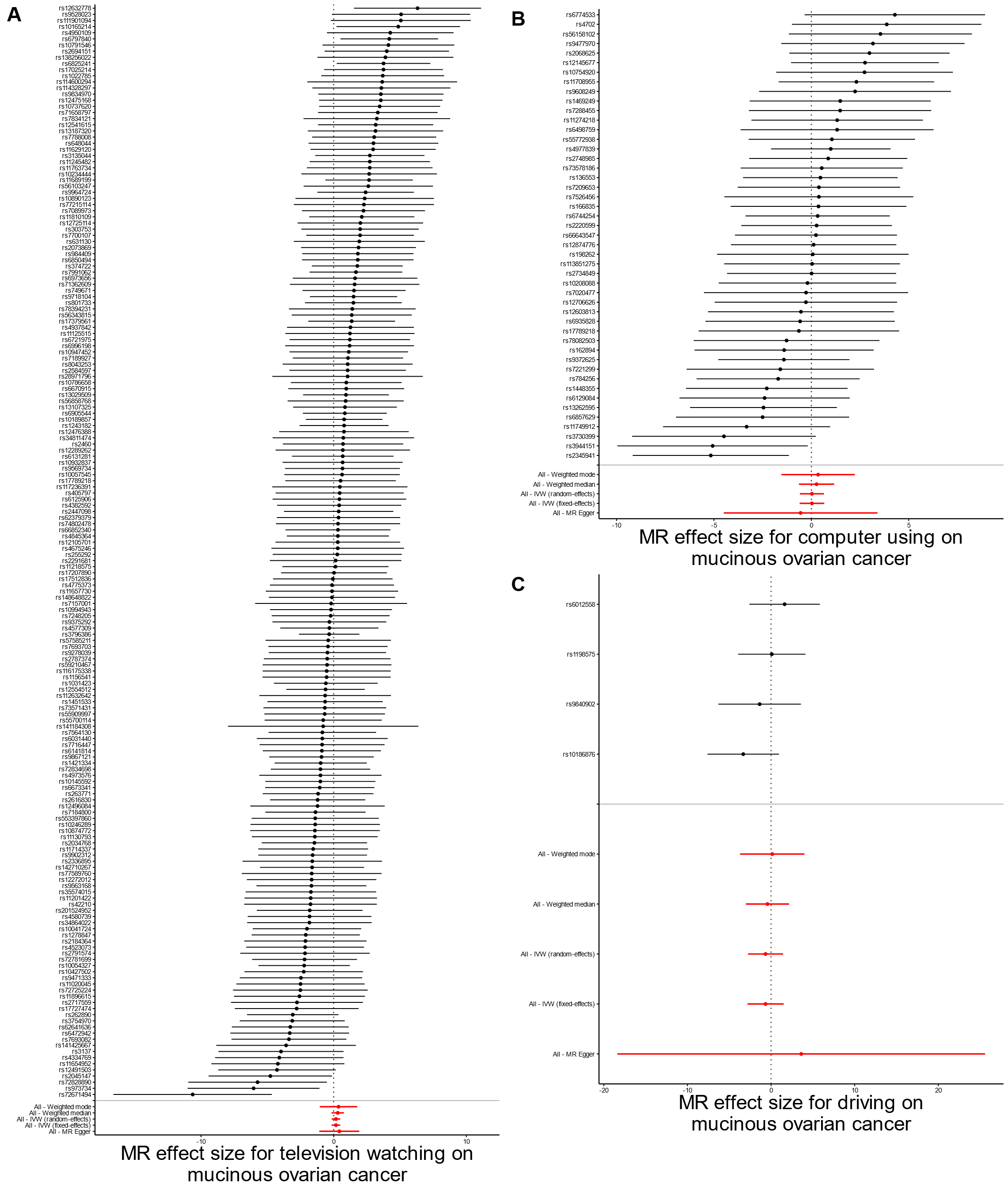


The MR single-SNP analysis plots the Wald estimate of causal association between (A) television watching and mucinous ovarian cancer, (B) computer using and mucinous ovarian cancer, (C) driving and mucinous ovarian cancer.

## eFigures of cervical cancer

### eFigure 52. Funnel plots of leisure sedentary behaviors and cervical cancer


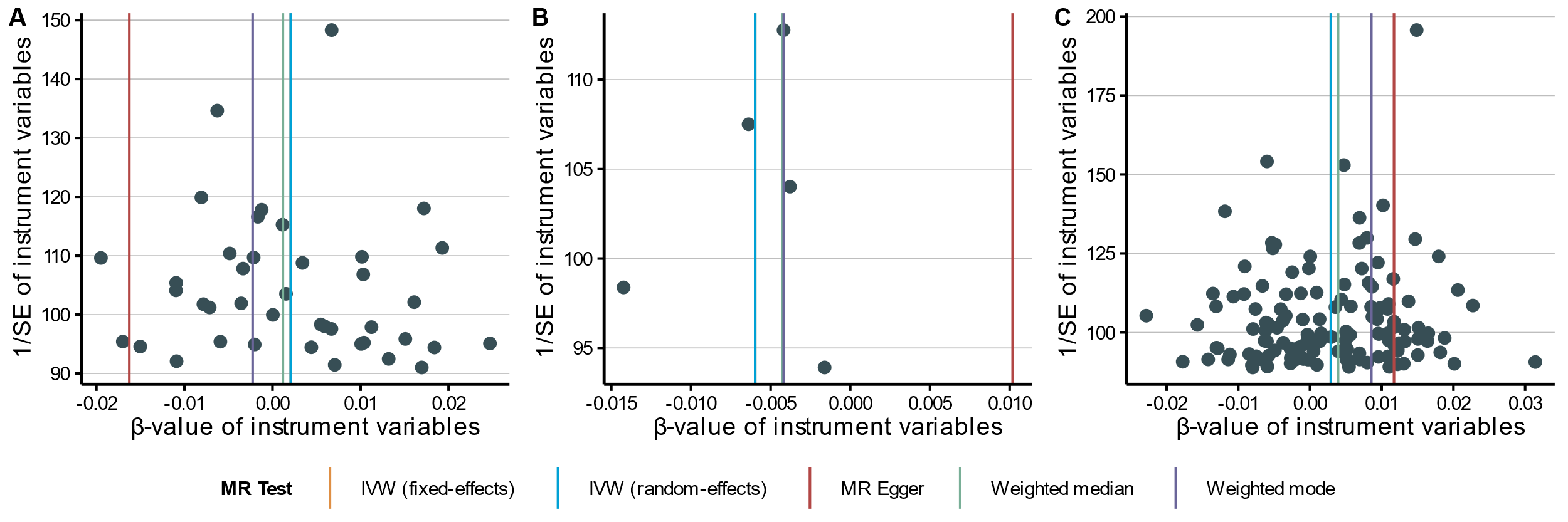


Funnel plots with colored vertical lines representing total MR estimation of causal associations between (A) computer using and cervical cancer, (B) driving and cervical cancer, (C) television watching and cervical cancer.

### eFigure 53. Scatter plots of leisure sedentary behaviors and cervical cancer


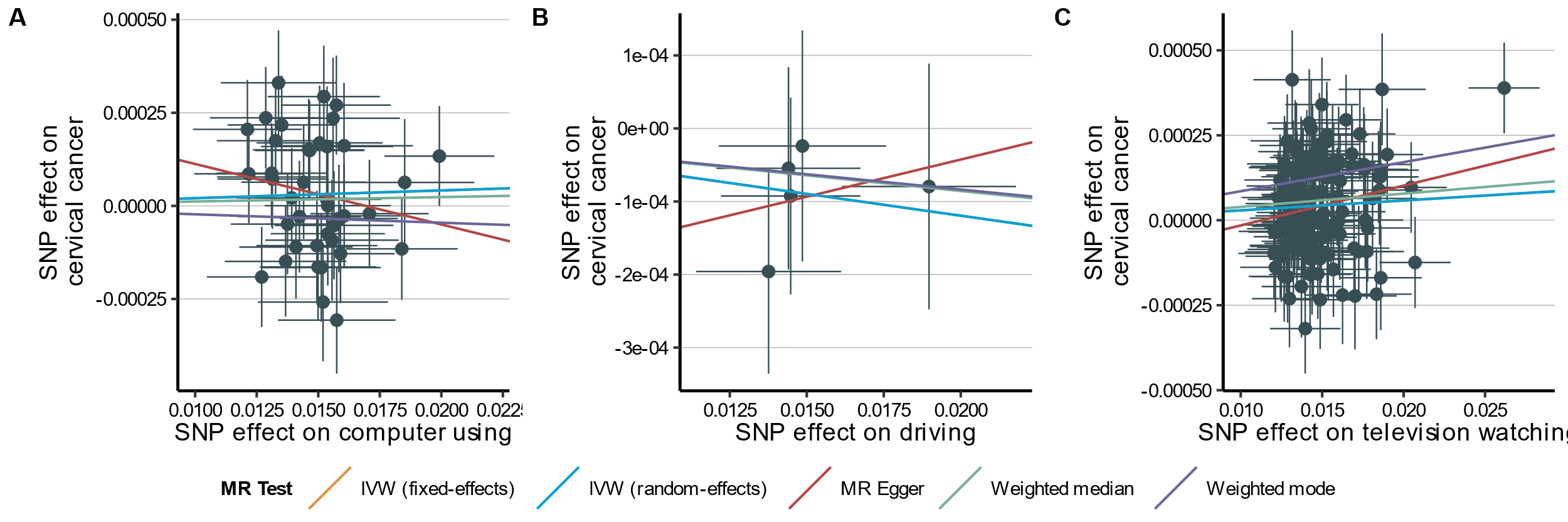


Scatter plots with colored lines representing results of each mendelian randomization sensitivity analysis between (A) computer using and cervical cancer, (B) driving and cervical cancer, (C) television watching and cervical cancer.

### eFigure 54. Leave-one-out plots of leisure sedentary behaviors and cervical cancer


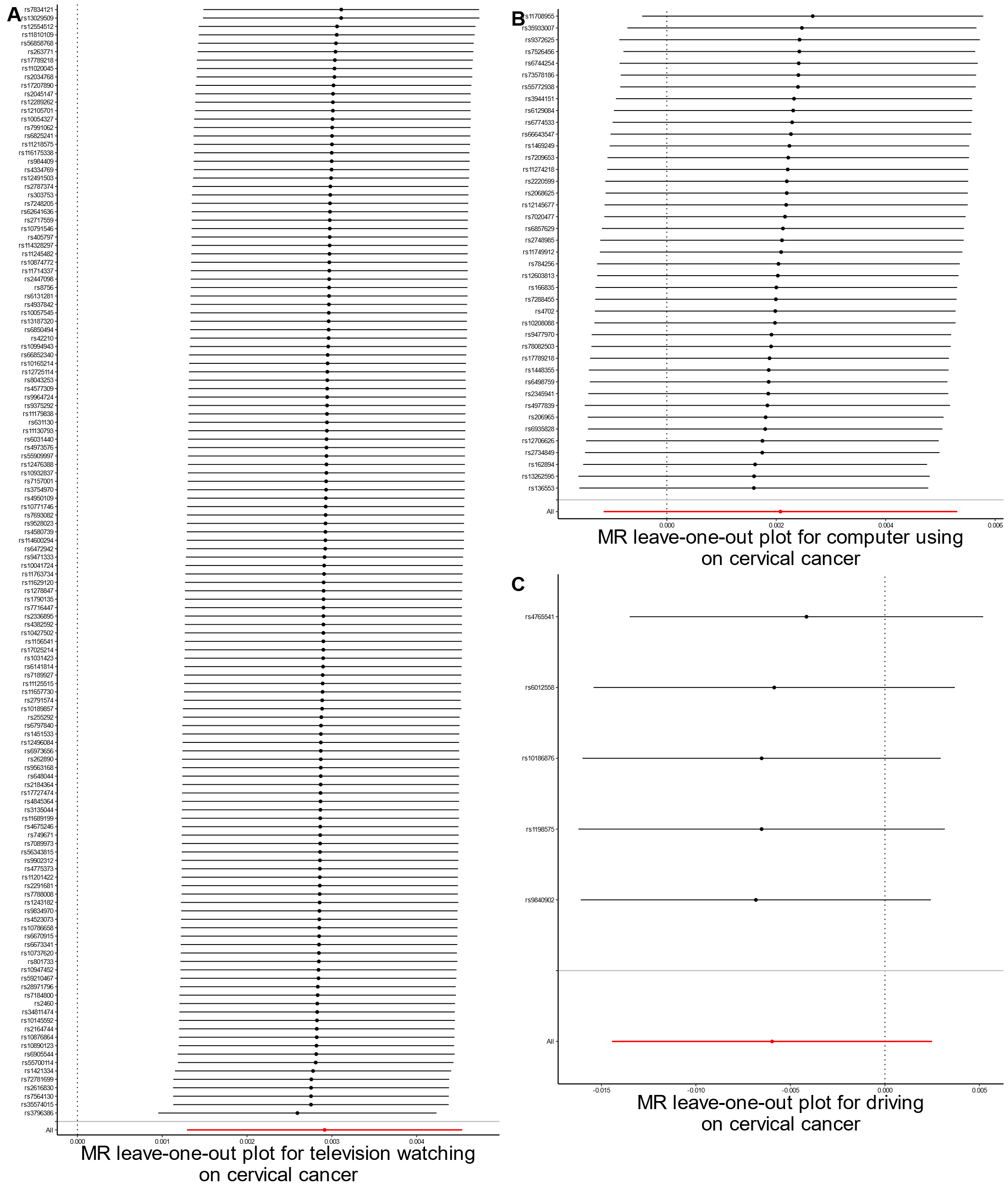


Leave-one-out plot of Mendelian randomization sensitivity analysis between (A) television watching and cervical cancer, (B) computer using and cervical cancer, (C) driving and cervical cancer.

### eFigure 55. Forest plots of single-SNP analysis of leisure sedentary behaviors and cervical cancer


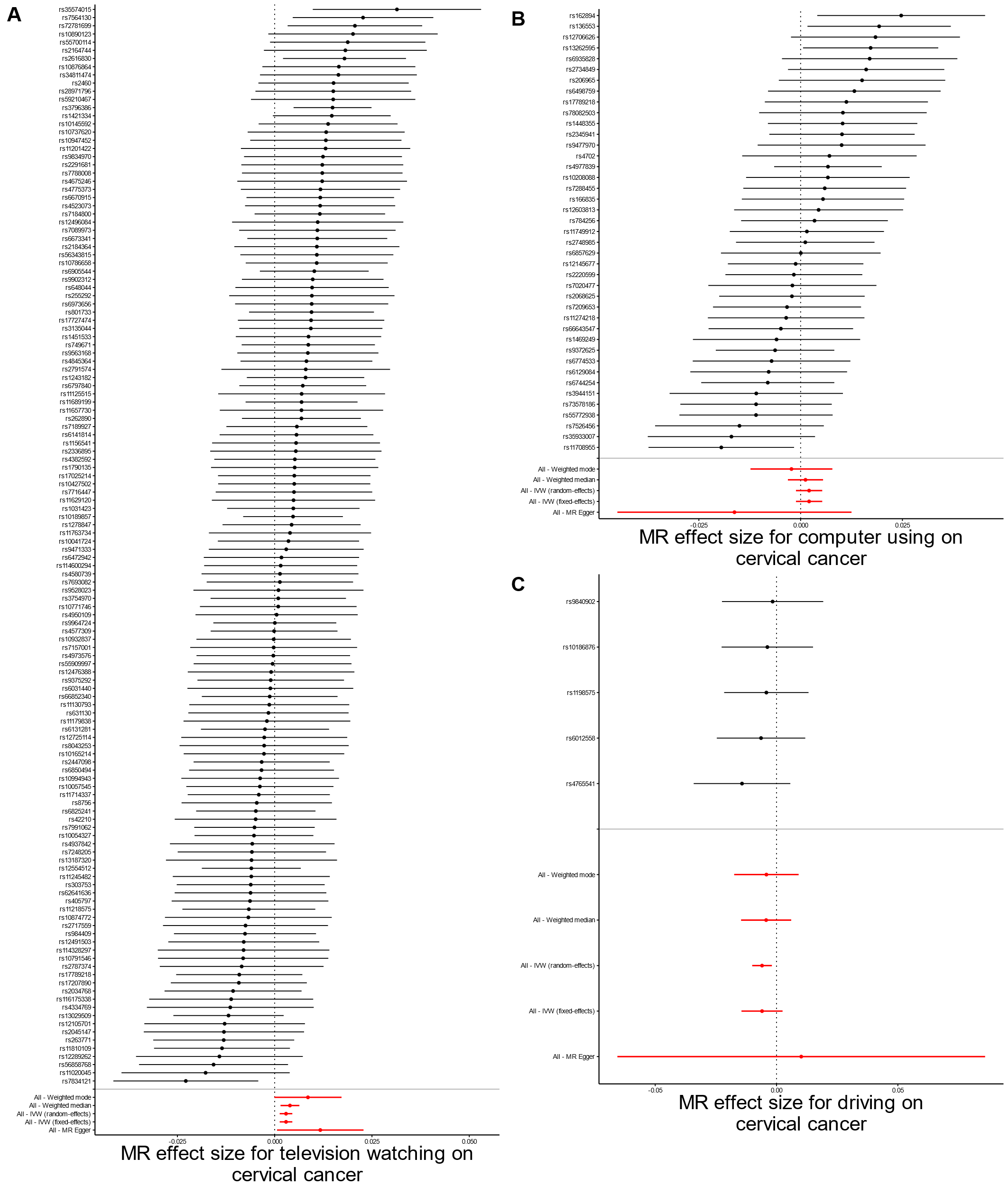


The MR single-SNP analysis plots the Wald estimate of causal association between (A) television watching and cervical cancer, (B) computer using and cervical cancer, (C) driving and cervical cancer.

## eFigures of prostate cancer

### eFigure 56. Funnel plots of leisure sedentary behaviors and prostate cancer


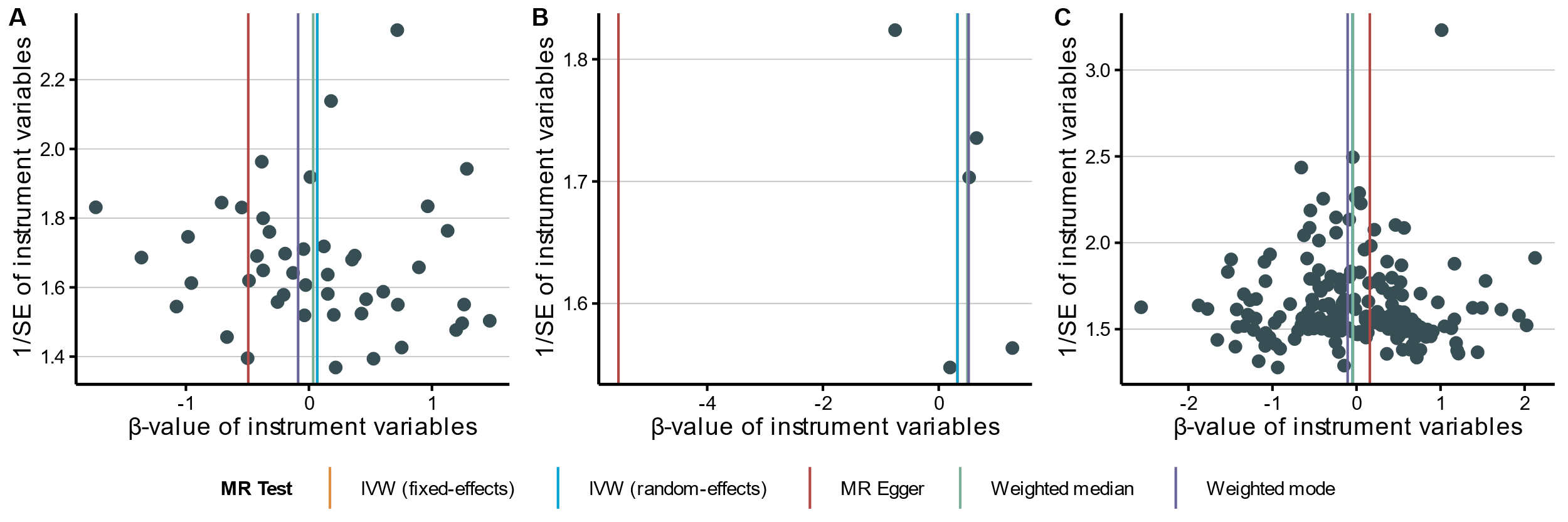


Funnel plots with colored vertical lines representing total MR estimation of causal associations between (A) computer using and prostate cancer, (B) driving and prostate cancer, (C) television watching and prostate cancer.

### eFigure 57. Scatter plots of leisure sedentary behaviors and prostate cancer


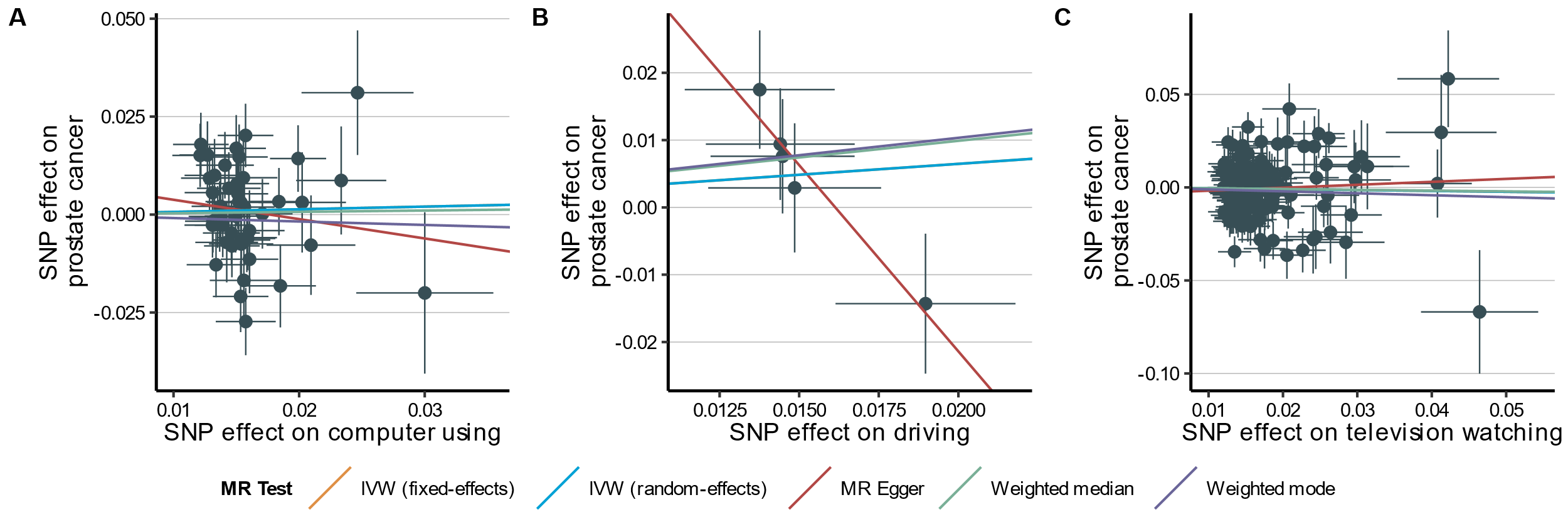


Scatter plots with colored lines representing results of each mendelian randomization sensitivity analysis between (A) computer using and prostate cancer, (B) driving and prostate cancer, (C) television watching and prostate cancer.

### eFigure 58. Leave-one-out plots of leisure sedentary behaviors and prostate cancer


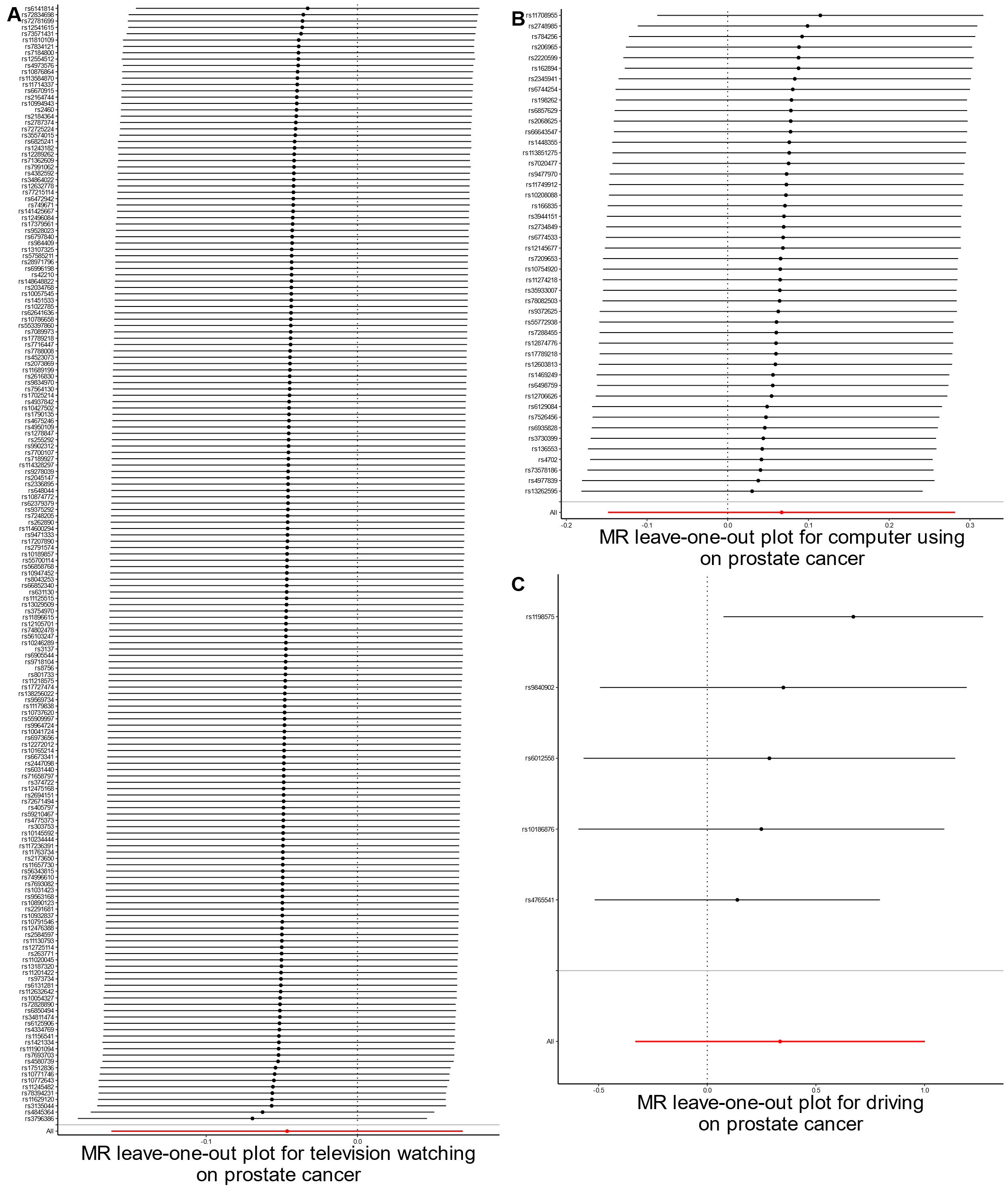


Leave-one-out plot of Mendelian randomization sensitivity analysis between (A) television watching and prostate cancer, (B) computer using and prostate cancer, (C) driving and prostate cancer.

### eFigure 59. Forest plots of single-SNP analysis of leisure sedentary behaviors and prostate cancer


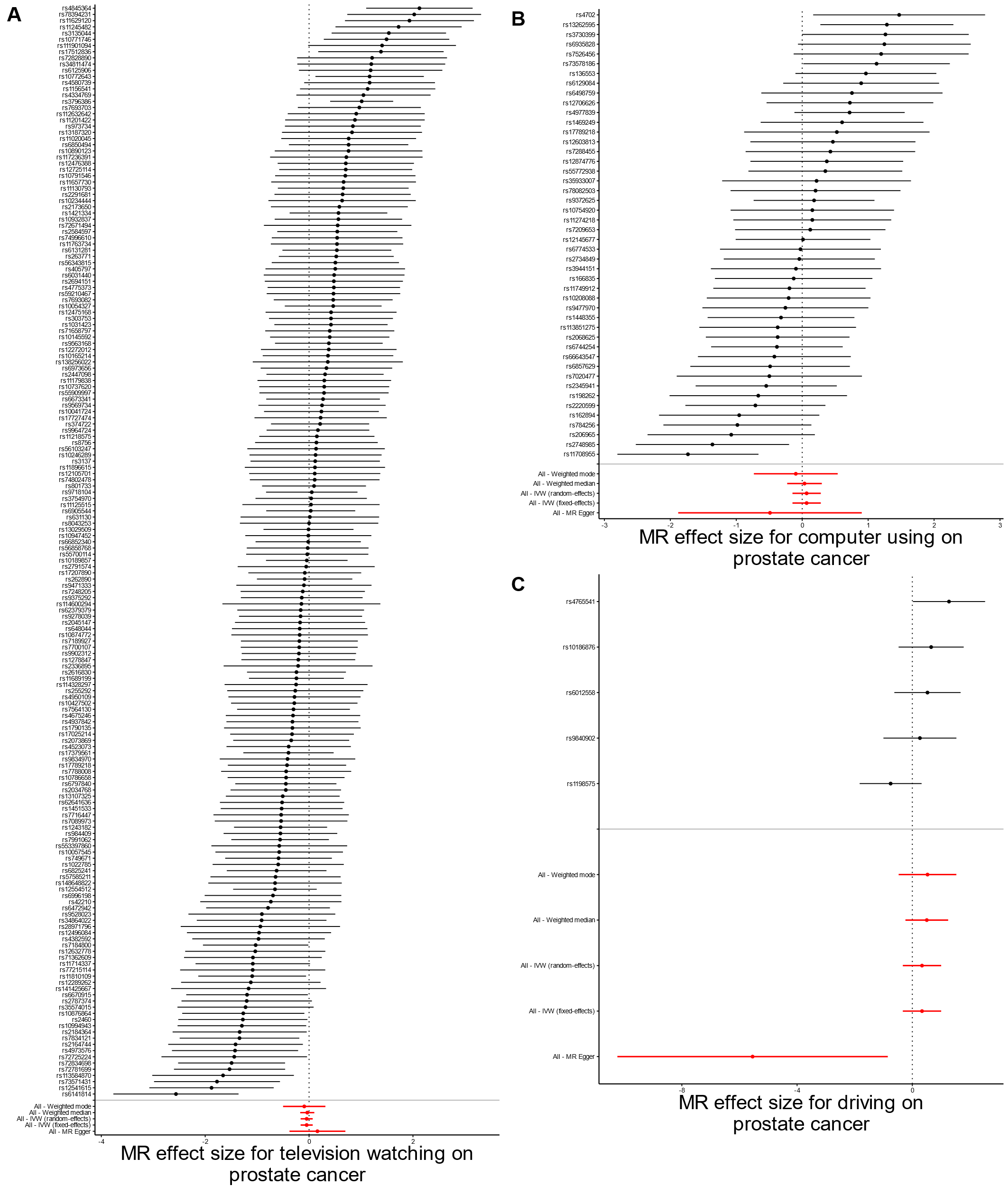


The MR single-SNP analysis plots the Wald estimate of causal association between (A) television watching and prostate cancer, (B) computer using and prostate cancer, (C) driving and prostate cancer.

## eFigures of esophageal cancer

### eFigure 60. Funnel plots of leisure sedentary behaviors and esophageal cancer


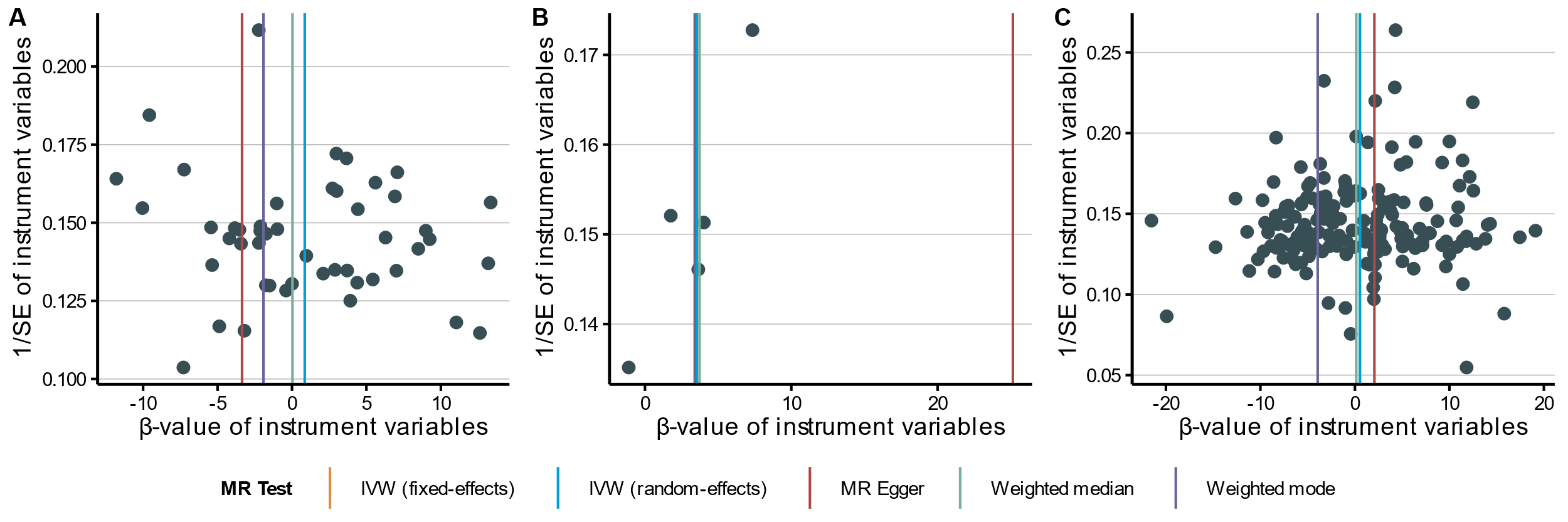


Funnel plots with colored vertical lines representing total MR estimation of causal associations between (A) computer using and esophageal cancer, (B) driving and esophageal cancer, (C) television watching and esophageal cancer.

### eFigure 61. Scatter plots of leisure sedentary behaviors and esophageal cancer


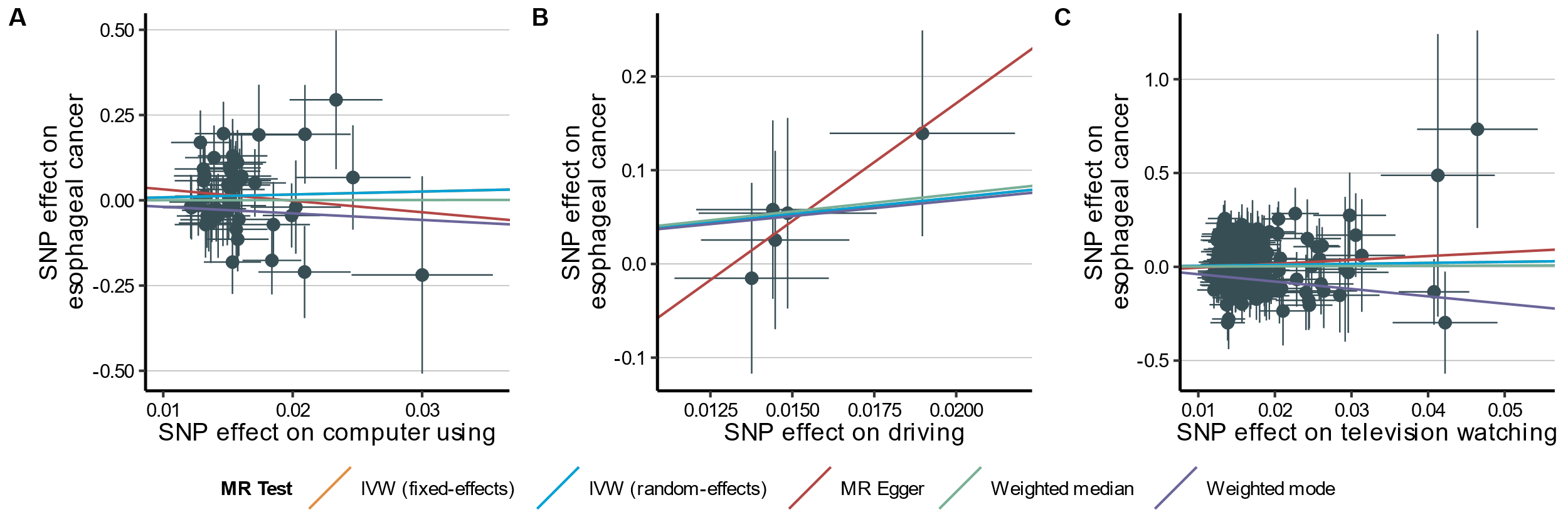


Scatter plots with colored lines representing results of each mendelian randomization sensitivity analysis between (A) computer using and esophageal cancer, (B) driving and esophageal cancer, (C) television watching and esophageal cancer.

### eFigure 62. Leave-one-out plots of leisure sedentary behaviors and esophageal cancer


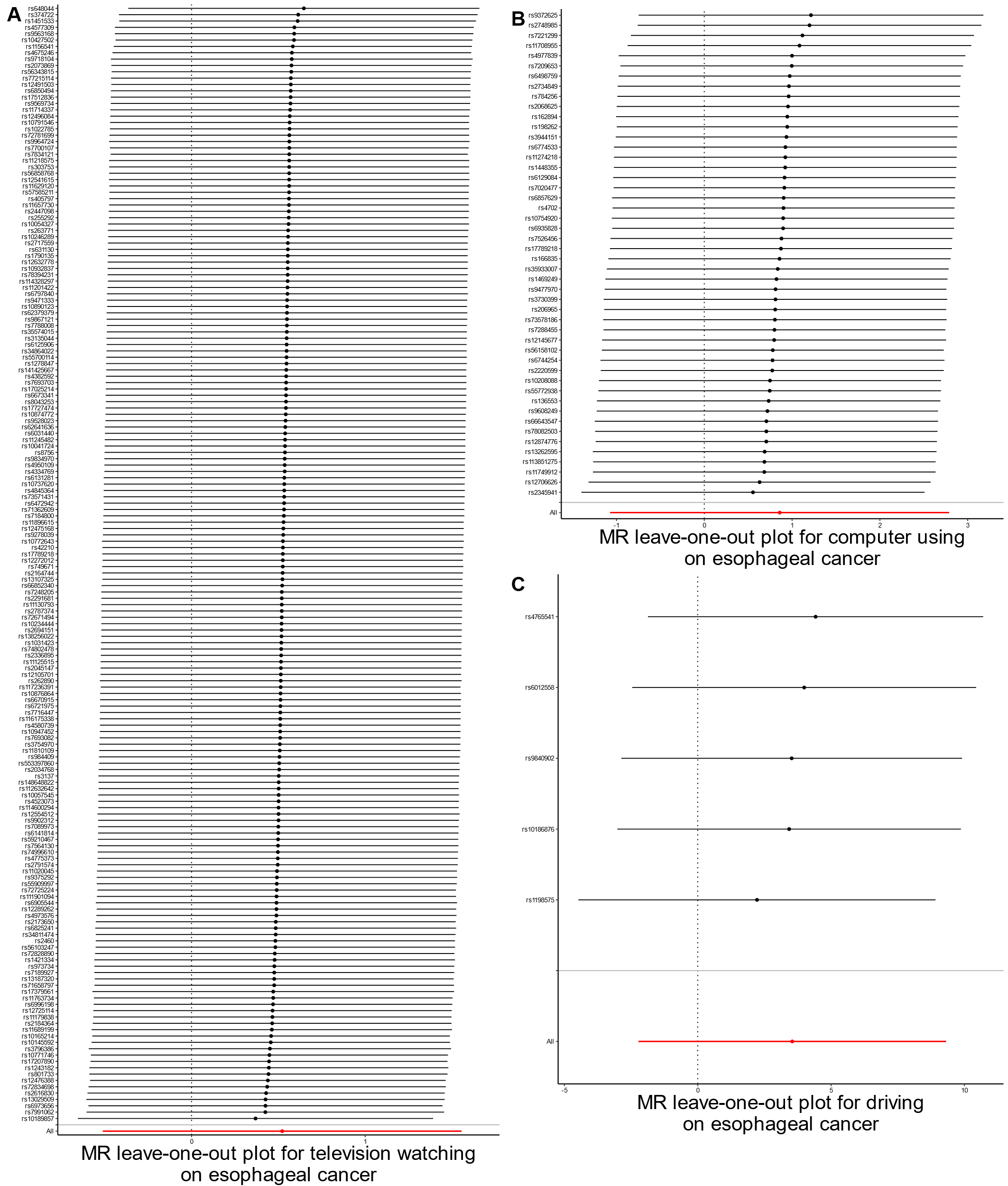


Leave-one-out plot of Mendelian randomization sensitivity analysis between (A) television watching and esophageal cancer, (B) computer using and esophageal cancer, (C) driving and esophageal cancer.

### eFigure 63. Forest plots of single-SNP analysis of leisure sedentary behaviors and esophageal cancer


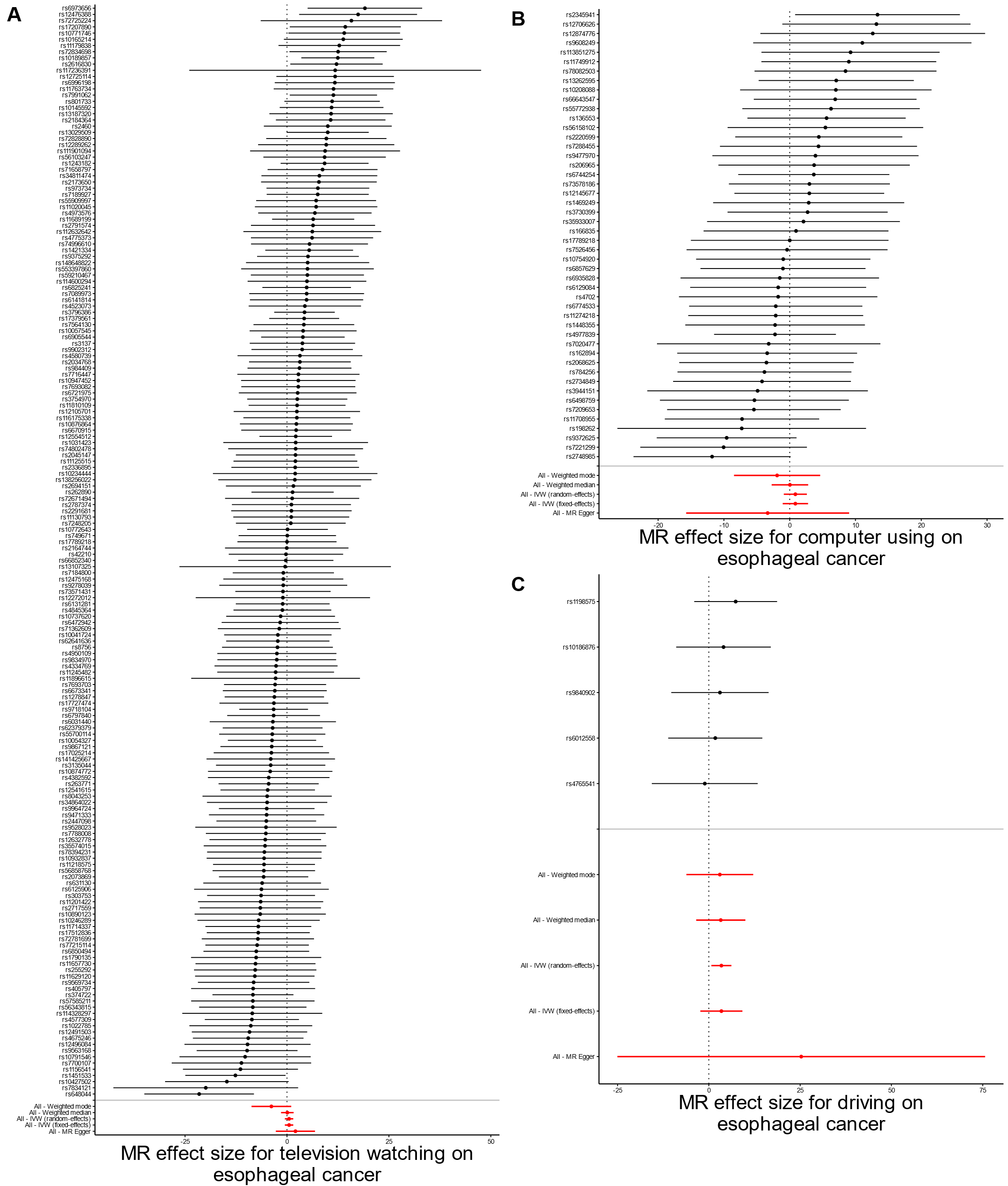


The MR single-SNP analysis plots the Wald estimate of causal association between (A) television watching and esophageal cancer, (B) computer using and esophageal cancer, (C) driving and esophageal cancer.

## eFigures of liver and intrahepatic bile ducts cancer

### eFigure 64. Funnel plots of leisure sedentary behaviors and cancer of liver and intrahepatic bile ducts


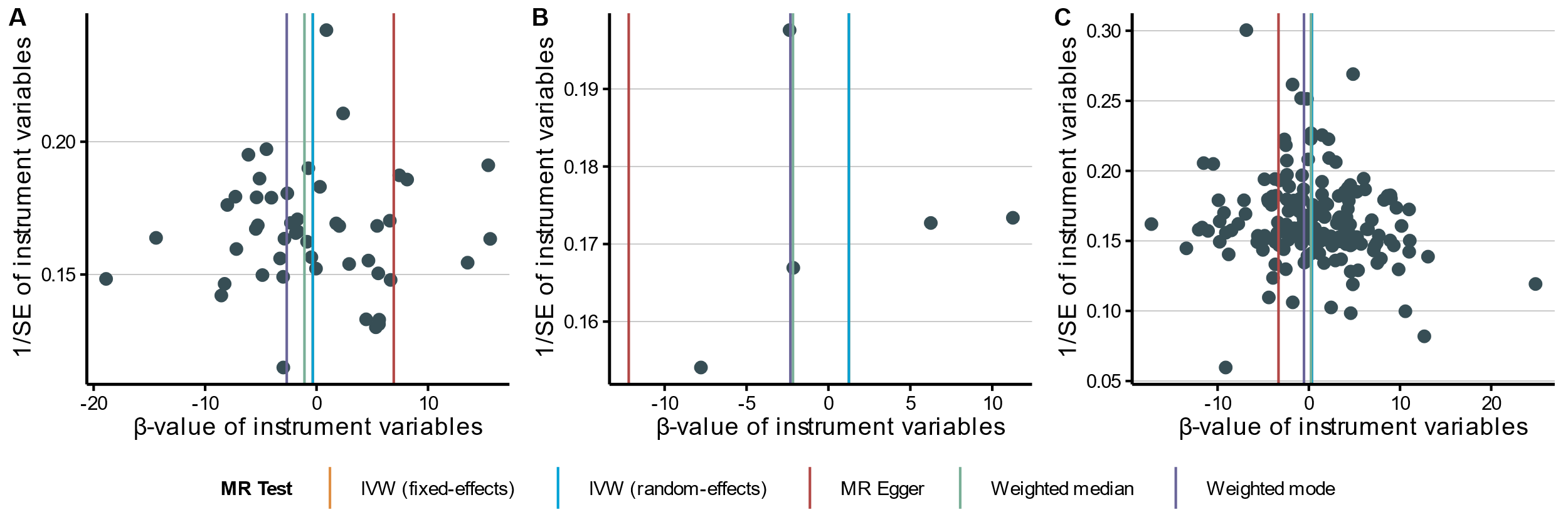


Funnel plots with colored vertical lines representing total MR estimation of causal associations between (A) computer using and cancer of liver and intrahepatic bile ducts, (B) driving and cancer of liver and intrahepatic bile ducts, (C) television watching and cancer of liver and intrahepatic bile ducts.

### eFigure 65. Scatter plots of leisure sedentary behaviors and cancer of liver and intrahepatic bile ducts


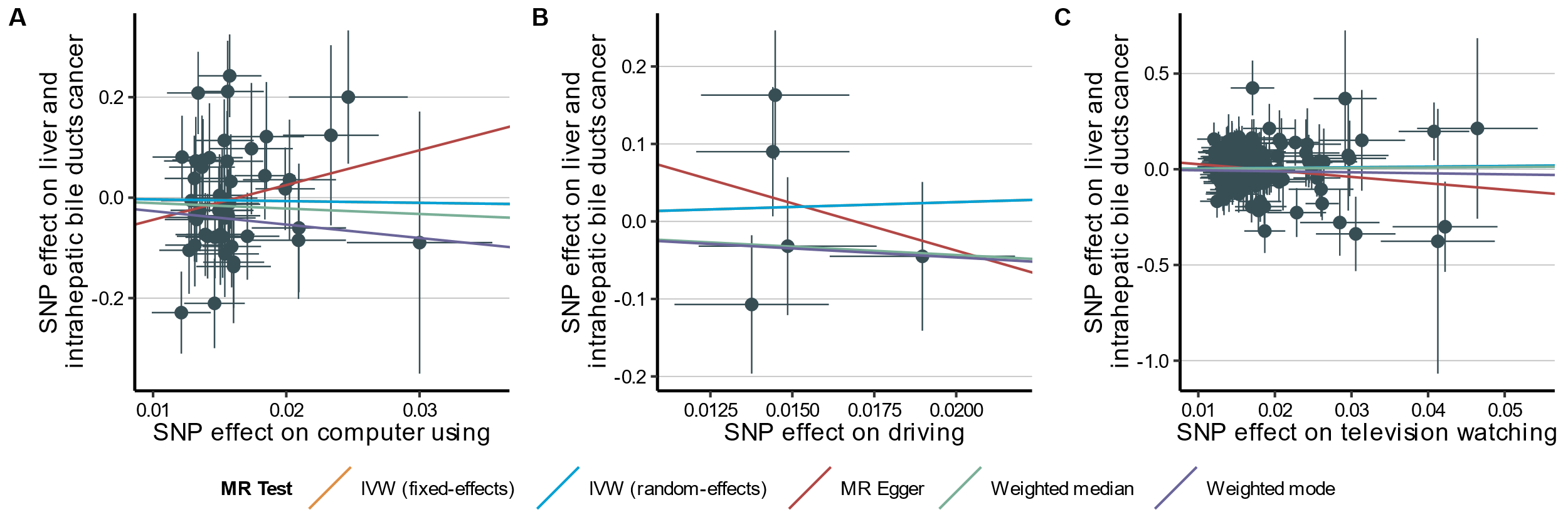


Scatter plots with colored lines representing results of each mendelian randomization sensitivity analysis between (A) computer using and cancer of liver and intrahepatic bile ducts, (B) driving and cancer of liver and intrahepatic bile ducts, (C) television watching and cancer of liver and intrahepatic bile ducts.

### eFigure 66. Leave-one-out plots of leisure sedentary behaviors and cancer of liver and intrahepatic bile ducts


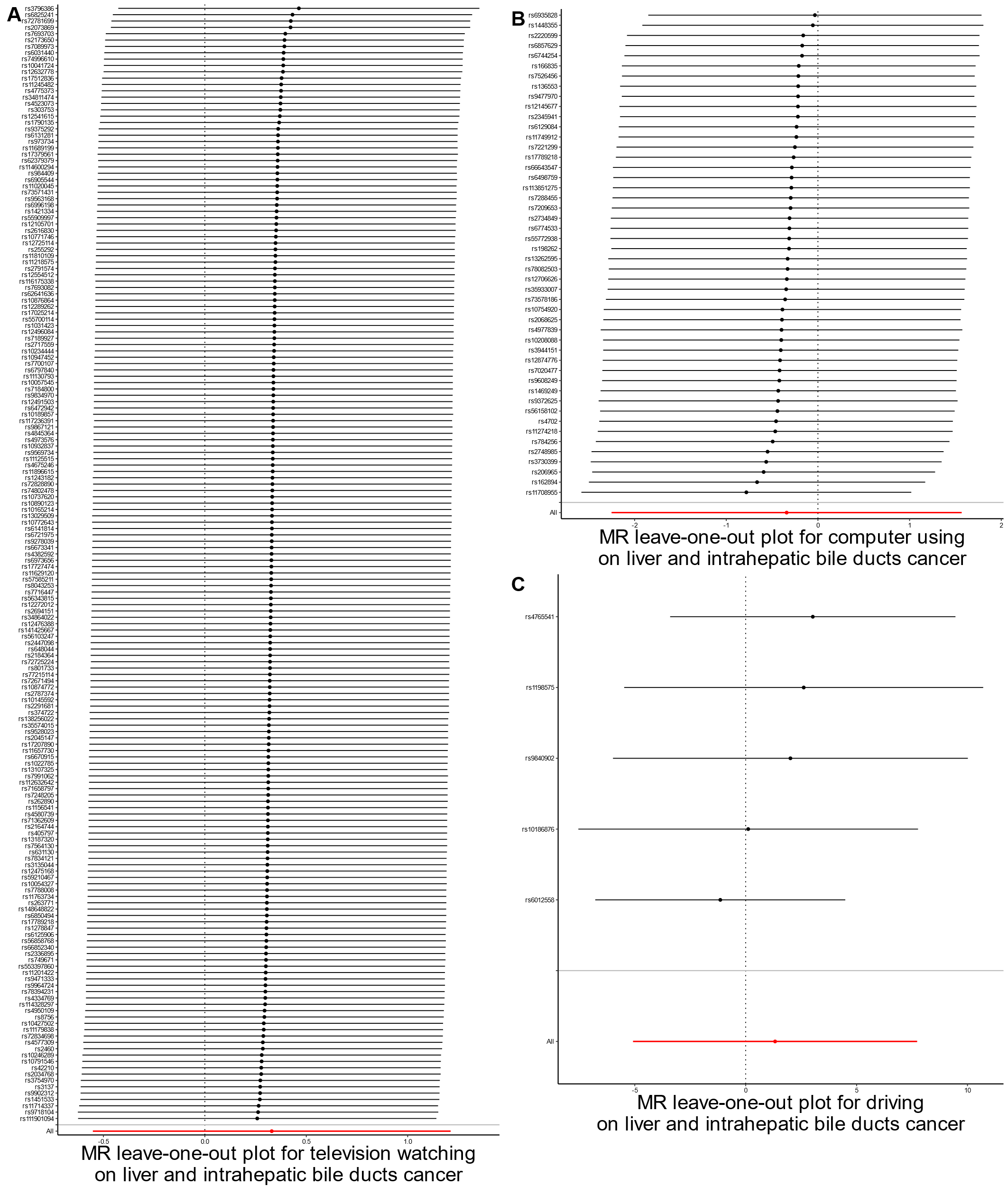


Leave-one-out plot of Mendelian randomization sensitivity analysis between (A) television watching and cancer of liver and intrahepatic bile ducts, (B) computer using and cancer of liver and intrahepatic bile ducts, (C) driving and cancer of liver and intrahepatic bile ducts.

### eFigure 67. Forest plots of single-SNP analysis of leisure sedentary behaviors and cancer of liver and intrahepatic bile ducts


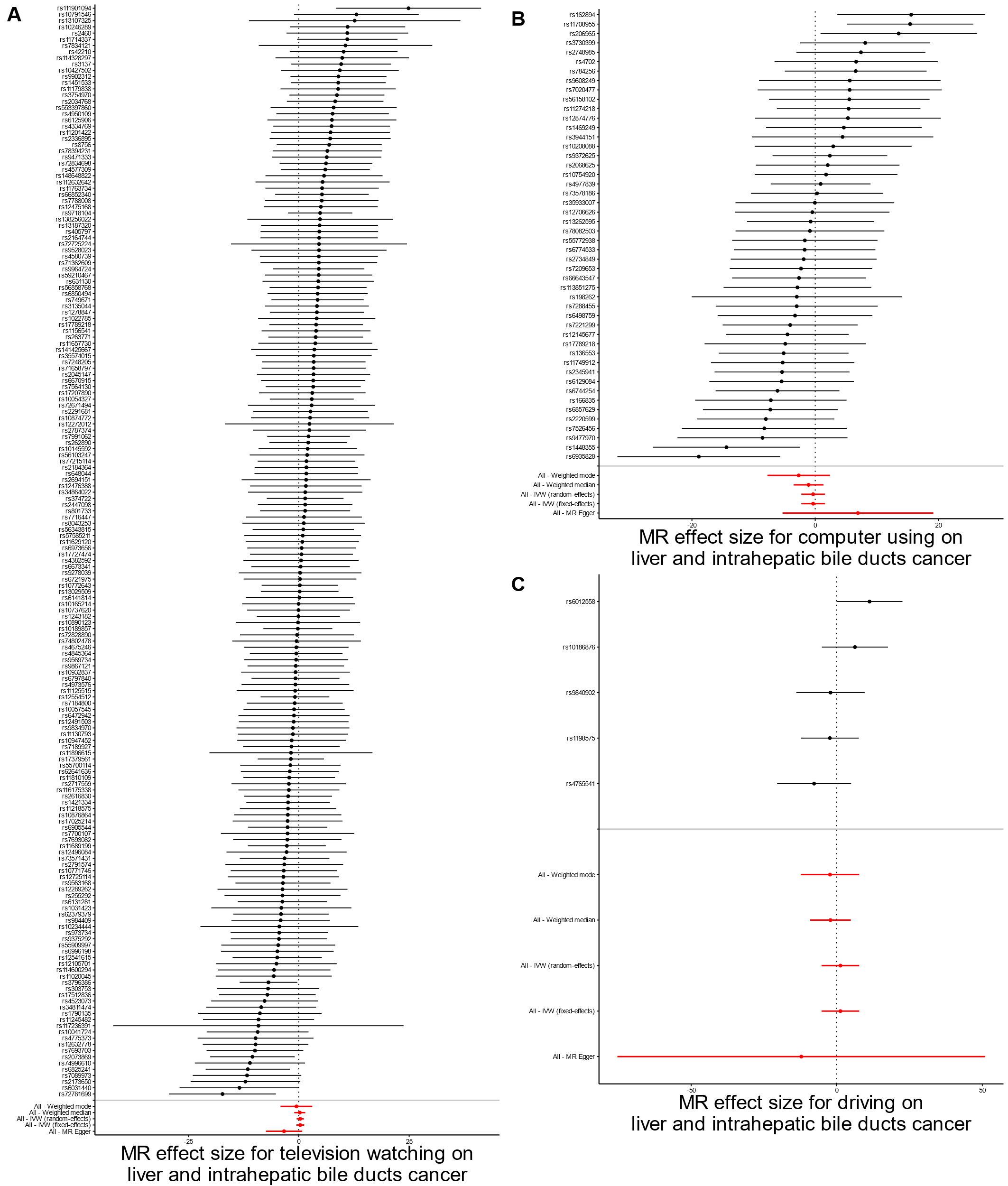


The MR single-SNP analysis plots the Wald estimate of causal association between (A) television watching and cancer of liver and intrahepatic bile ducts, (B) computer using and cancer of liver and intrahepatic bile ducts, (C) driving and cancer of liver and intrahepatic bile ducts.

## eFigures of liver and pancreatic cancer

### eFigure 68. Funnel plots of leisure sedentary behaviors and pancreatic cancer


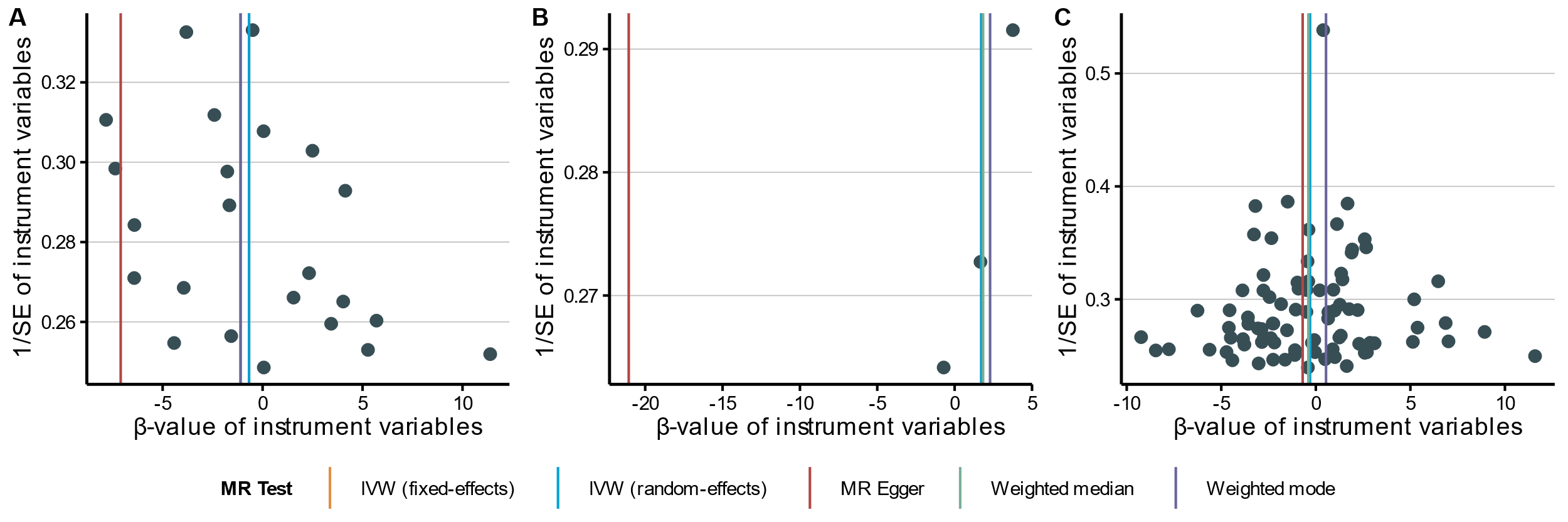


Funnel plots with colored vertical lines representing total MR estimation of causal associations between (A) computer using and pancreatic cancer, (B) driving and pancreatic cancer, (C) television watching and liver and pancreatic cancer.

### eFigure 69. Scatter plots of leisure sedentary behaviors and pancreatic cancer


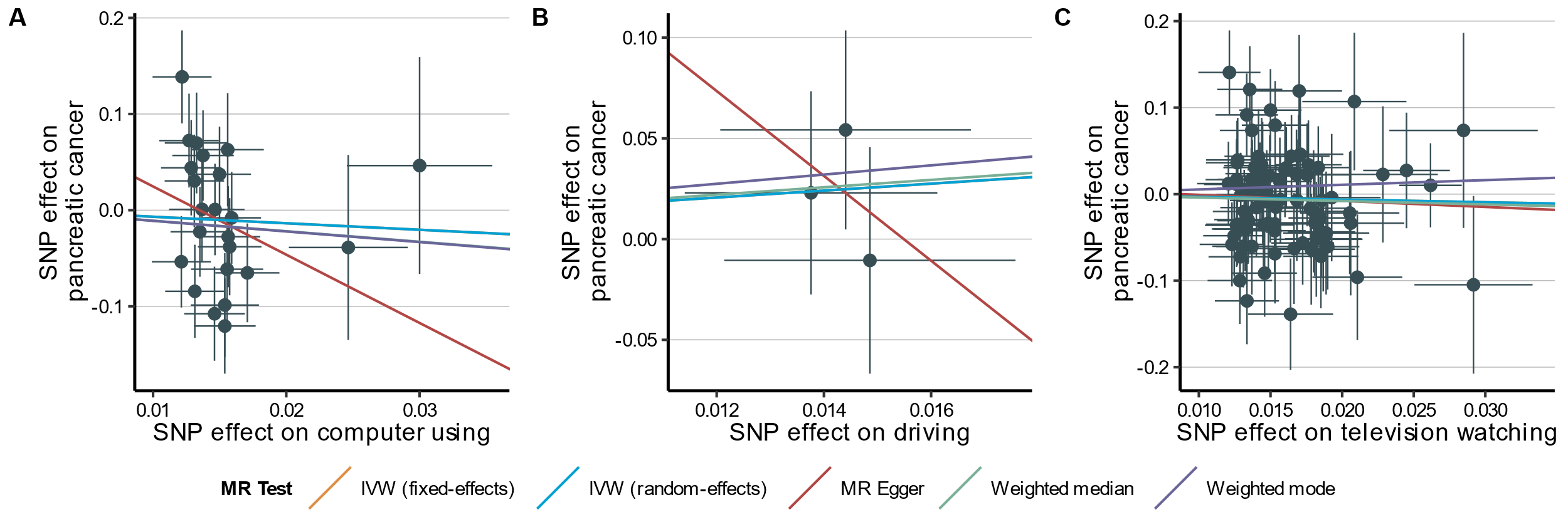


Scatter plots with colored lines representing results of each mendelian randomization sensitivity analysis between (A) computer using and pancreatic cancer, (B) driving and pancreatic cancer, (C) television watching and pancreatic cancer.

### eFigure 70. Leave-one-out plots of leisure sedentary behaviors and pancreatic cancer


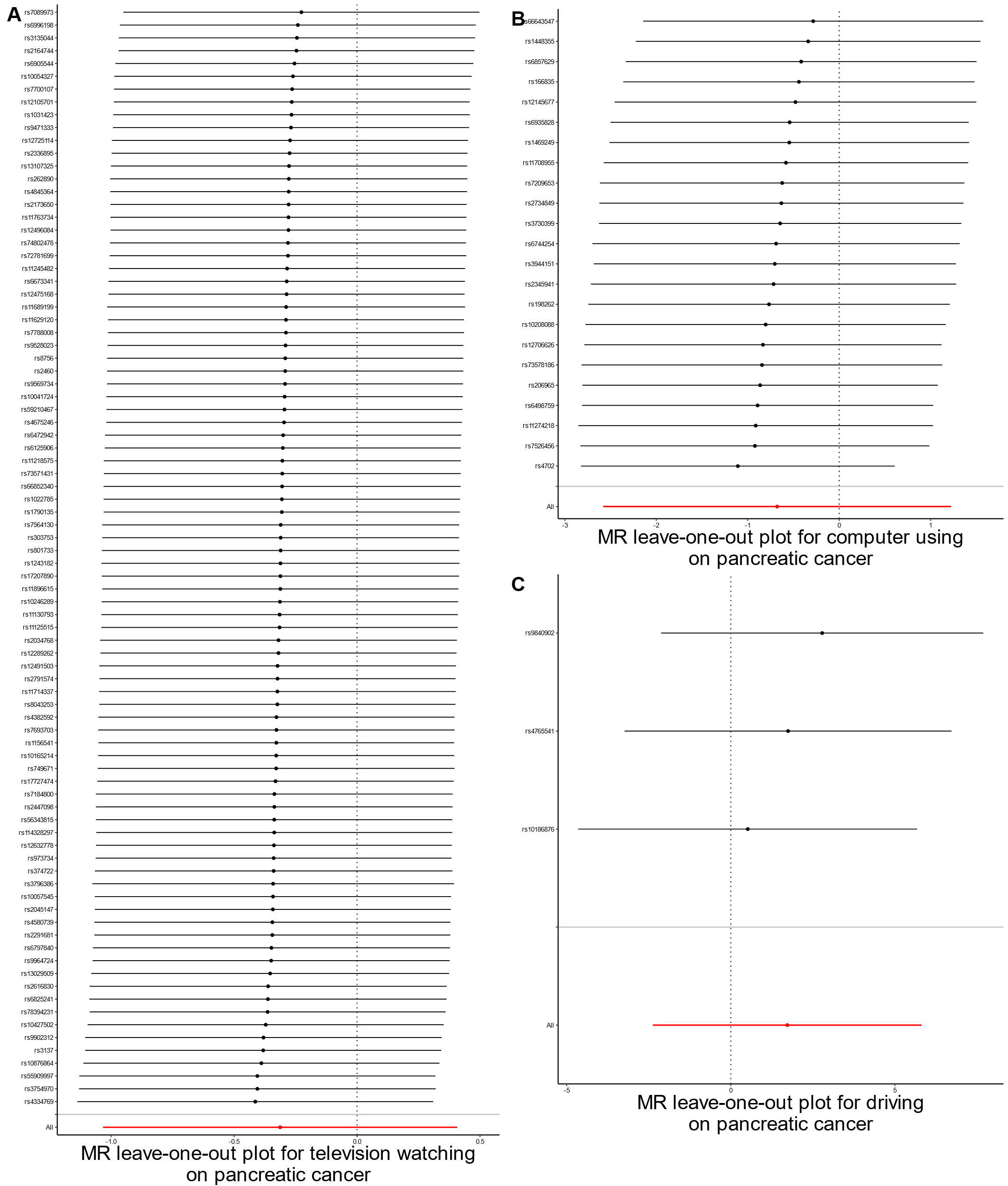


Leave-one-out plot of Mendelian randomization sensitivity analysis between (A) television watching and pancreatic cancer, (B) computer using and pancreatic cancer, (C) driving and pancreatic cancer.

### eFigure 71. Forest plots of single-SNP analysis of leisure sedentary behaviors and pancreatic cancer


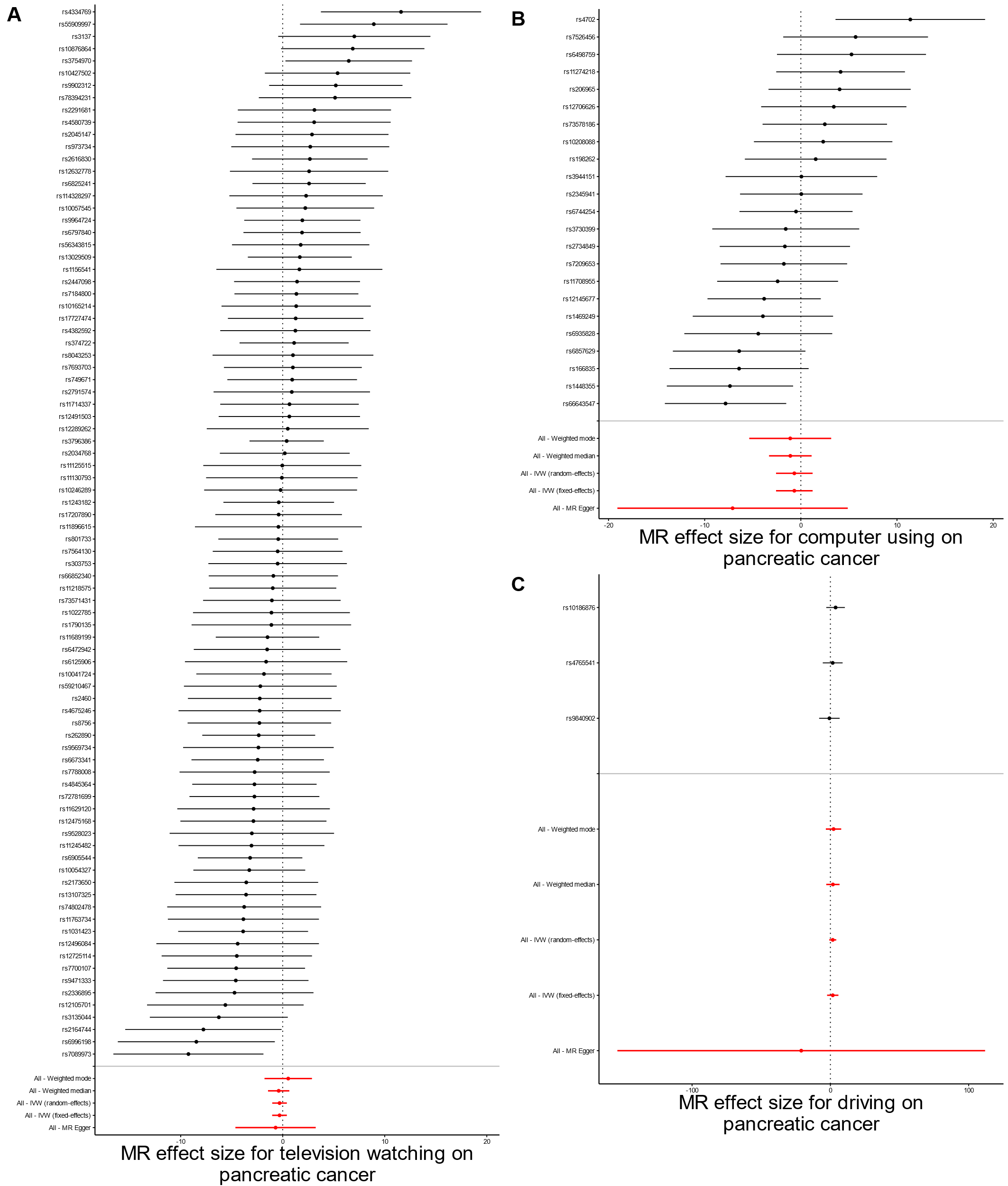


The MR single-SNP analysis plots the Wald estimate of causal association between (A) television watching and pancreatic cancer, (B) computer using and pancreatic cancer, (C) driving and pancreatic cancer.

## eFigures of oral and pharyngeal cancer

### eFigure 72. Funnel plots of leisure sedentary behaviors and cancer of oral cavity and pharynx


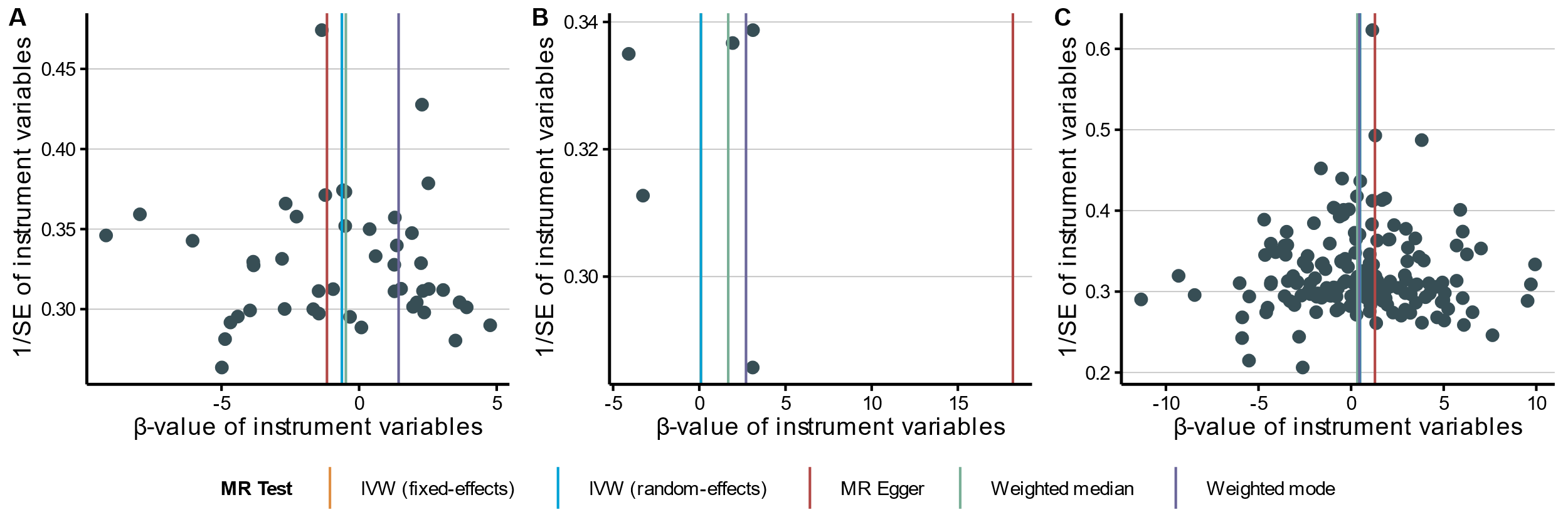


Funnel plots with colored vertical lines representing total MR estimation of causal associations between (A) computer using and cancer of oral cavity and pharynx, (B) driving and cancer of oral cavity and pharynx, (C) television watching and cancer of oral cavity and pharynx.

### eFigure 73. Scatter plots of leisure sedentary behaviors and cancer of oral cavity and pharyngeal


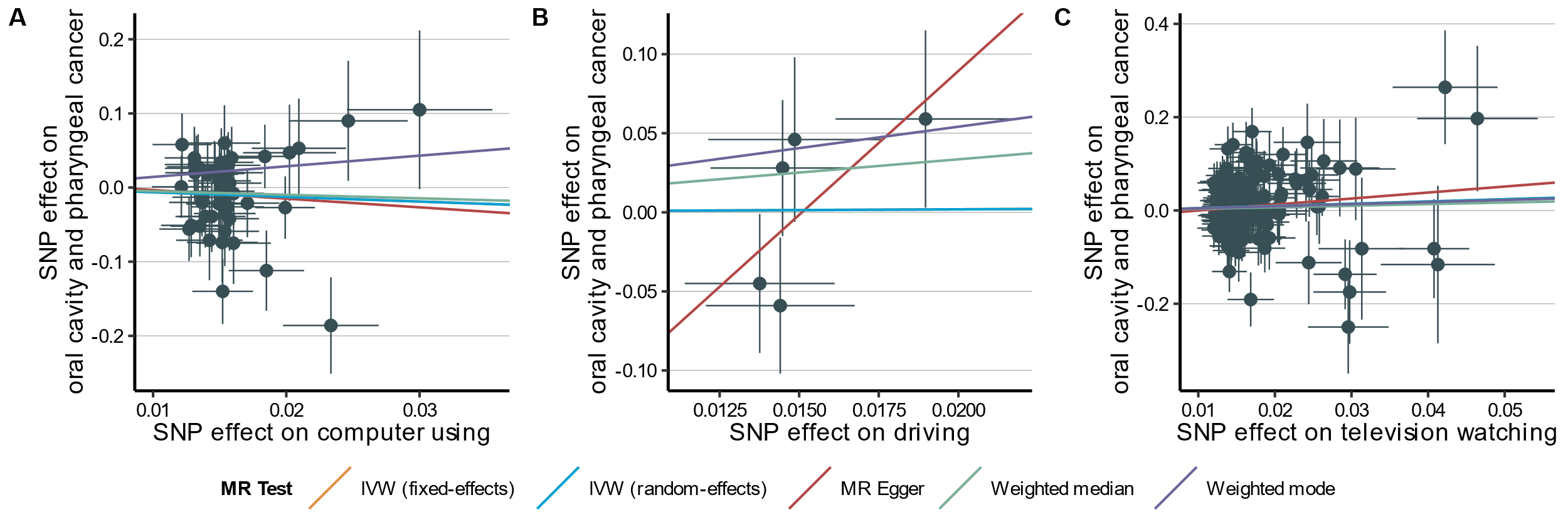


Scatter plots with colored lines representing results of each mendelian randomization sensitivity analysis between (A) computer using and cancer of oral cavity and pharynx, (B) driving and cancer of oral cavity and pharynx, (C) television watching and cancer of oral cavity and pharynx.

### eFigure 74. Leave-one-out plots of leisure sedentary behaviors and cancer of oral cavity and pharynx


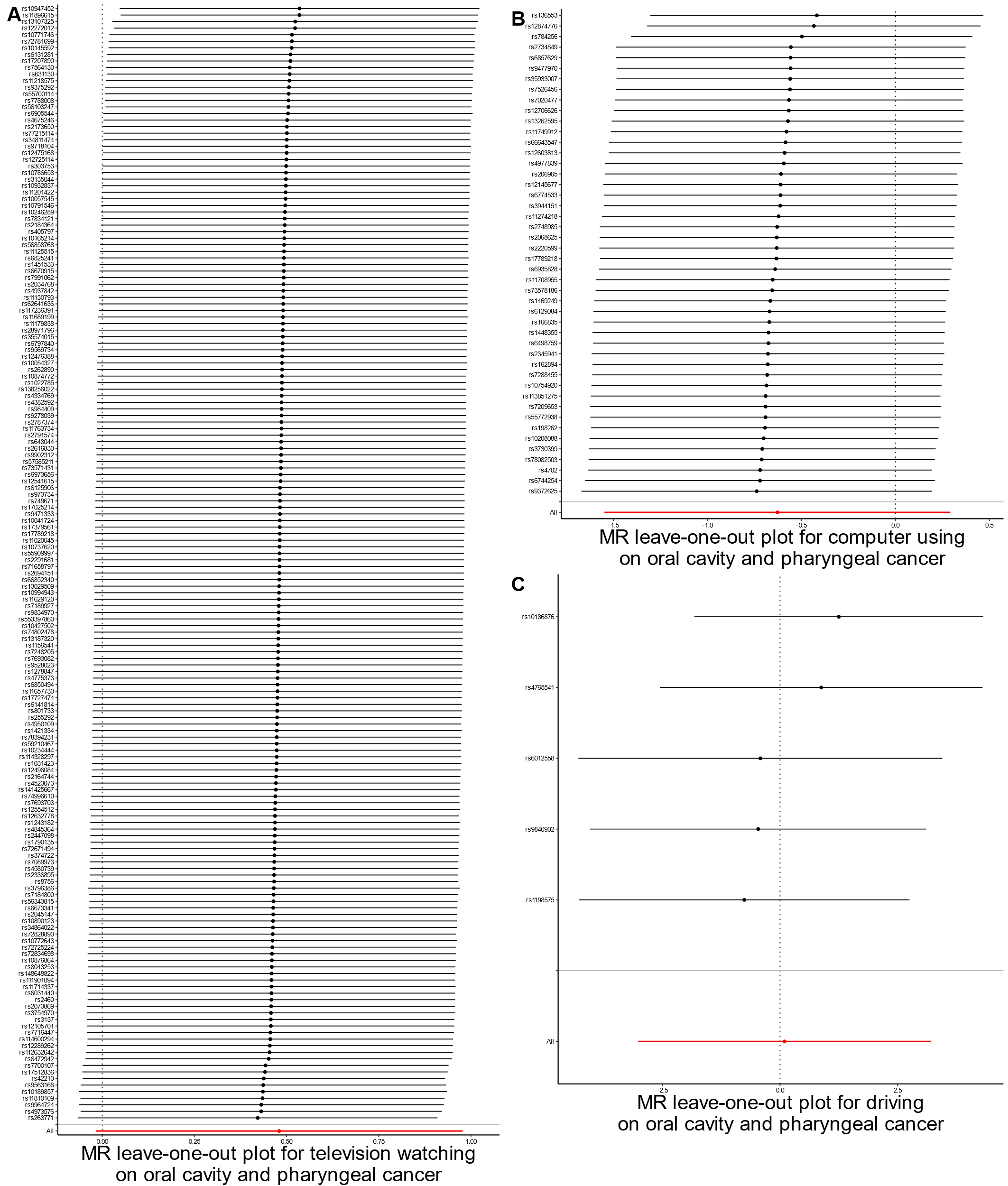


Leave-one-out plot of Mendelian randomization sensitivity analysis between (A) television watching and cancer of oral cavity and pharynx, (B) computer using and cancer of oral cavity and pharynx, (C) driving and cancer of oral cavity and pharynx.

### eFigure 75. Forest plots of single-SNP analysis of leisure sedentary behaviors and cancer of oral cavity and pharynx


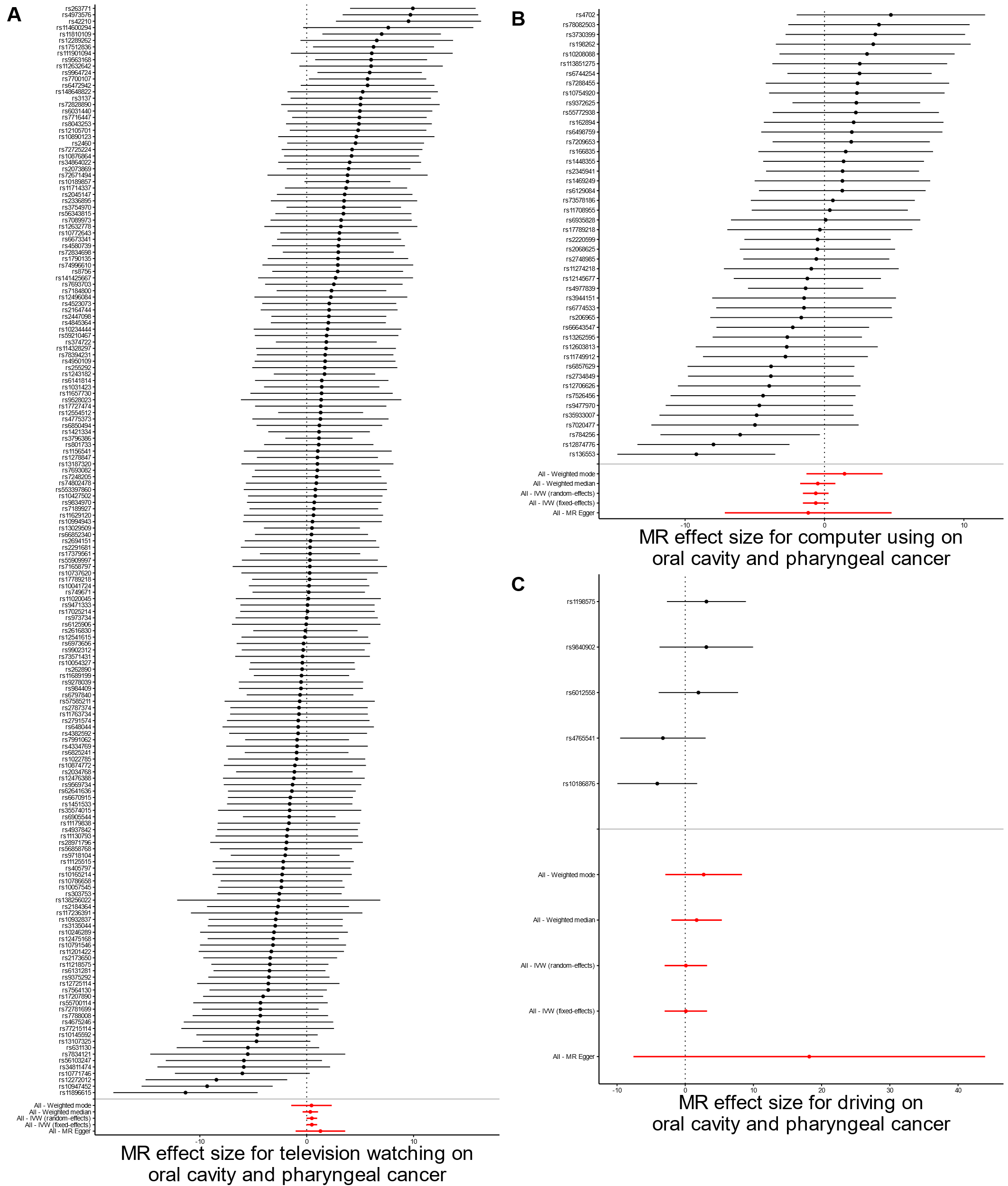


The MR single-SNP analysis plots the Wald estimate of causal association between (A) television watching and cancer of oral cavity and pharynx, (B) computer using and cancer of oral cavity and pharynx, (C) driving and cancer of oral cavity and pharynx.

## eFigures of urinary organs cancer

### eFigure 76. Scatter plots of leisure sedentary behaviors and cancer of urinary organs


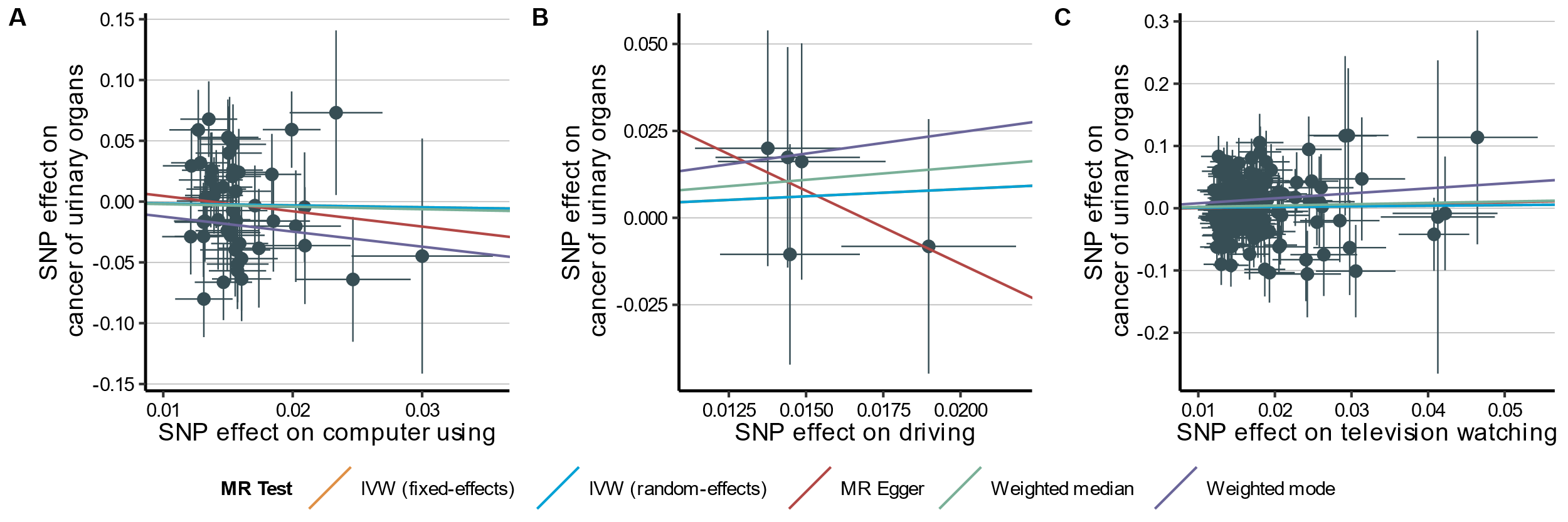


Scatter plots with colored lines representing results of each mendelian randomization sensitivity analysis between (A) computer using and cancer of urinary organs, (B) driving and cancer of urinary organs, (C) television watching and cancer of urinary organs.

### eFigure 77. Funnel plots of leisure sedentary behaviors and cancer of urinary organs


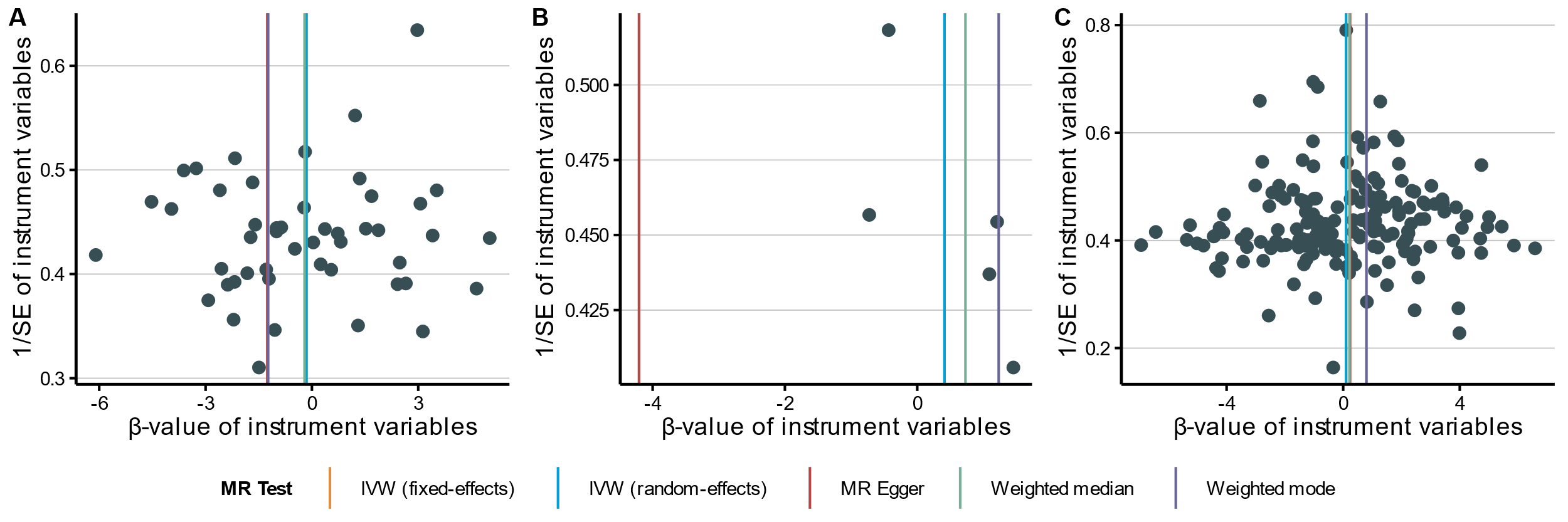


Funnel plots with colored vertical lines representing total MR estimation of causal associations between (A) computer using and cancer of urinary organs, (B) driving and cancer of urinary organs, (C) television watching and cancer of urinary organs.

### eFigure 78. Leave-one-out plots of leisure sedentary behaviors and cancer of urinary organs


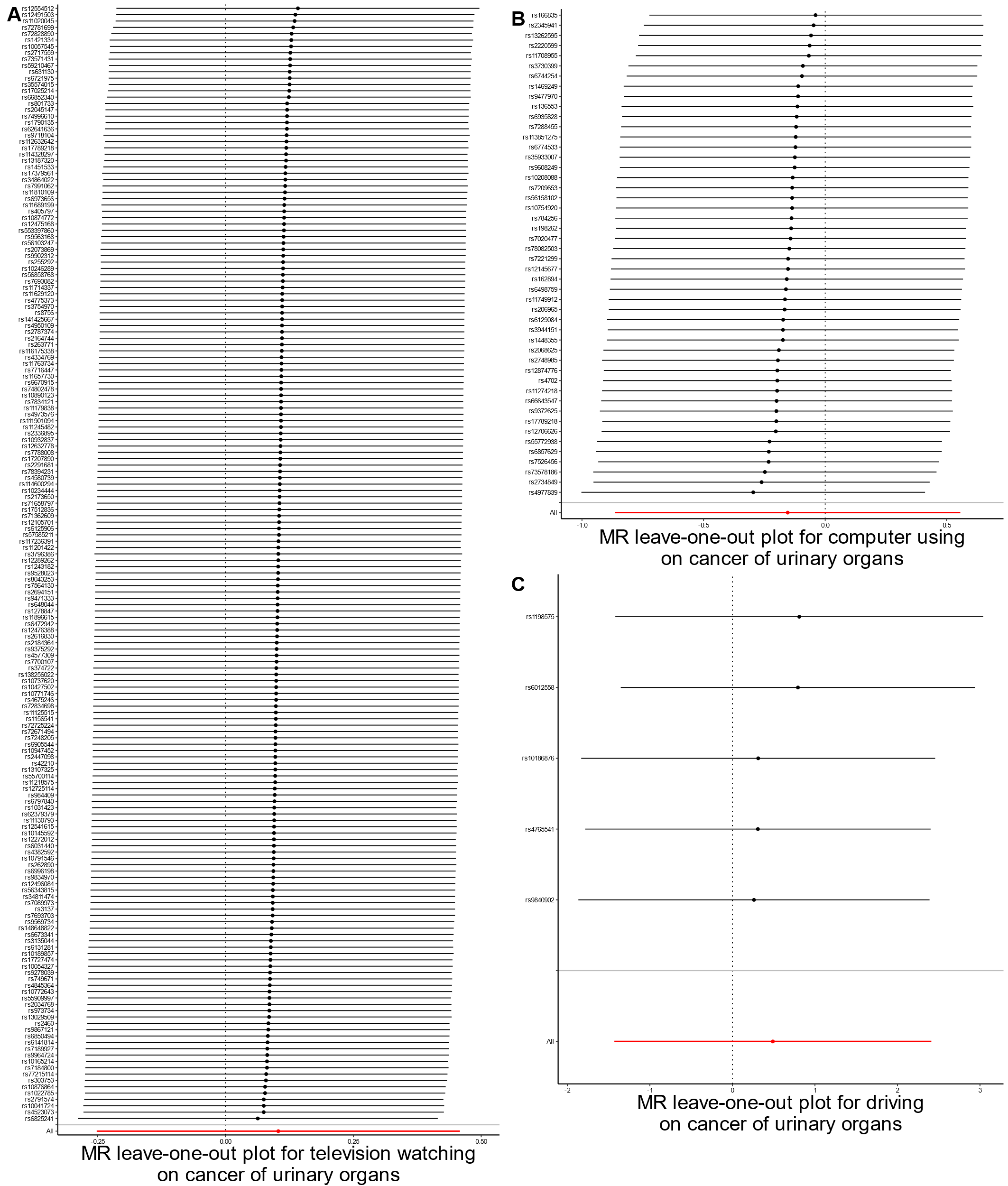


Leave-one-out plot of Mendelian randomization sensitivity analysis between (A) television watching and cancer of urinary organs, (B) computer using and cancer of urinary organs, (C) driving and cancer of urinary organs.

### eFigure 79. Forest plots of single-SNP analysis of leisure sedentary behaviors and cancer of urinary organs


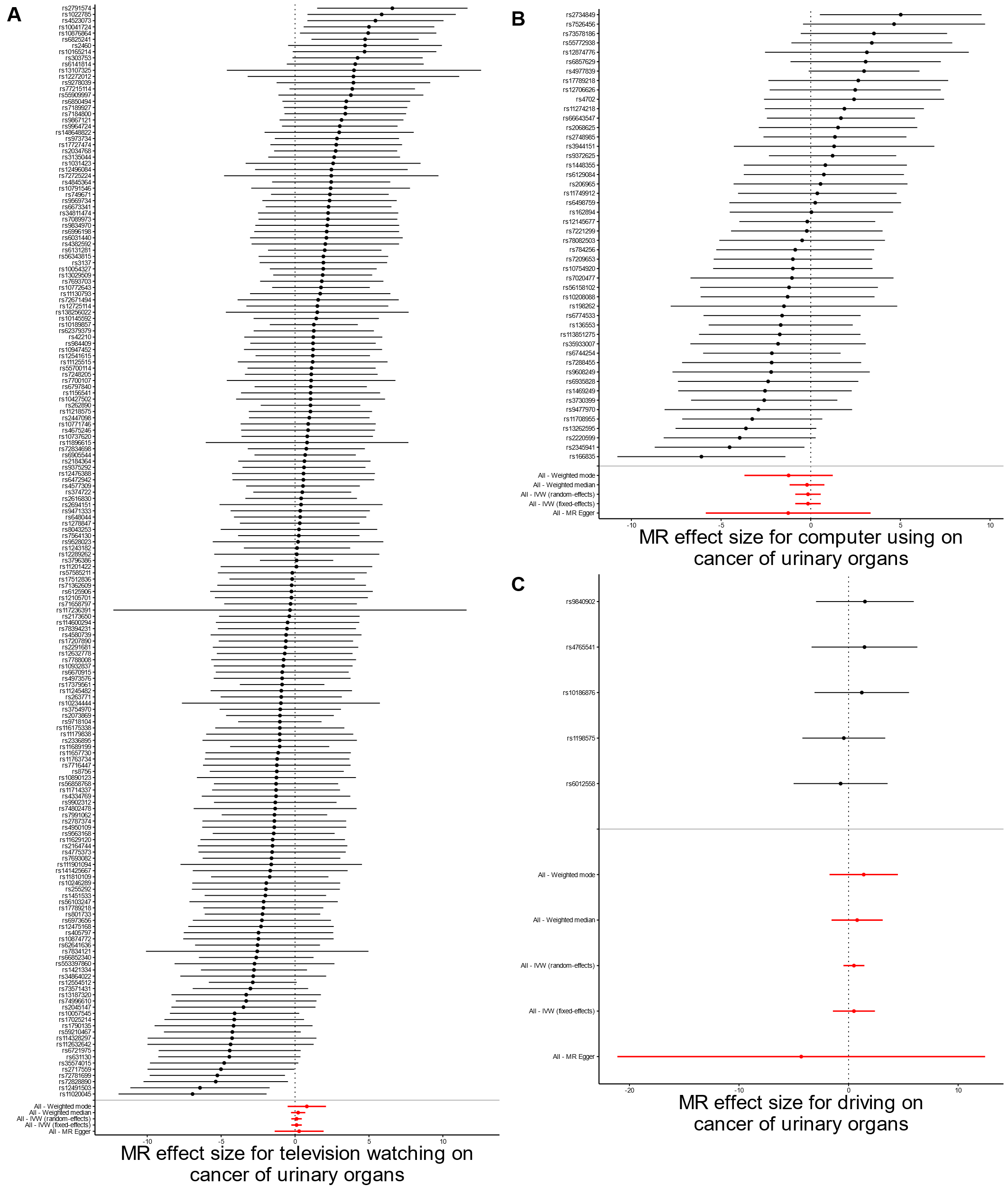


The MR single-SNP analysis plots the Wald estimate of causal association between (A) television watching and cancer of urinary organs, (B) computer using and cancer of urinary organs, (C) driving and cancer of urinary organs.

## eFigures of colon cancer

### eFigure 80. Scatter plots of leisure sedentary behaviors and colon cancer


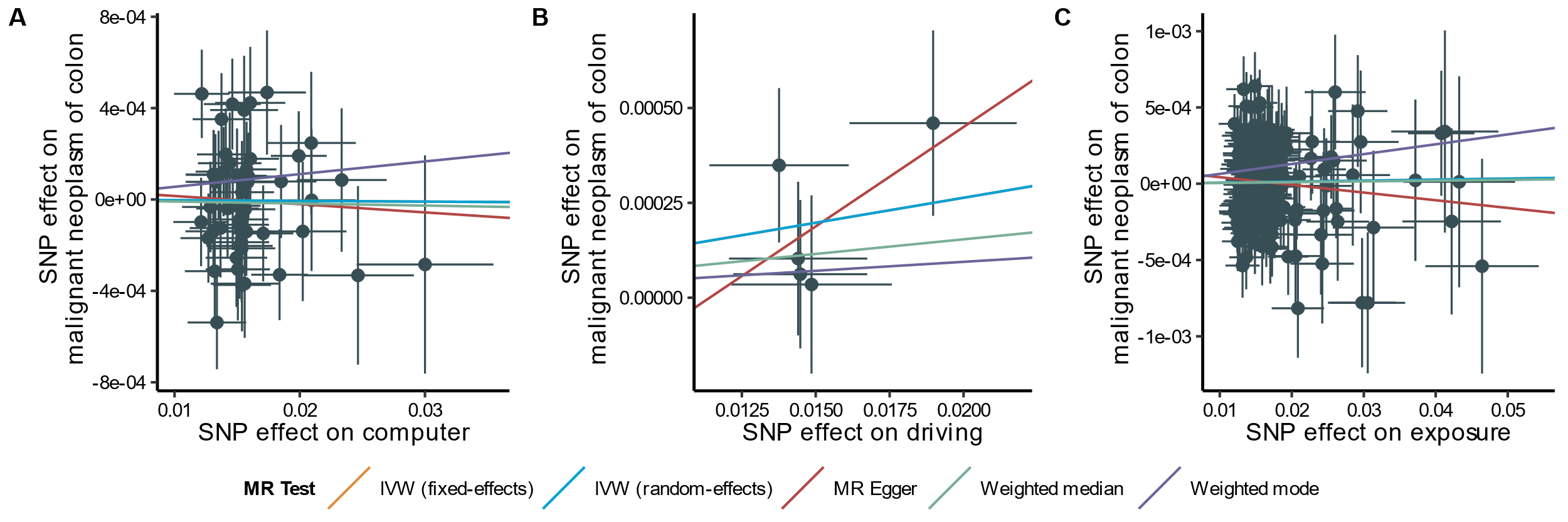


Scatter plots with colored lines representing results of each mendelian randomization sensitivity analysis between (A) computer using and colon cancer, (B) driving and colon cancer, (C) television watching and colon cancer.

### eFigure 81. Funnel plots of leisure sedentary behaviors and colon cancer


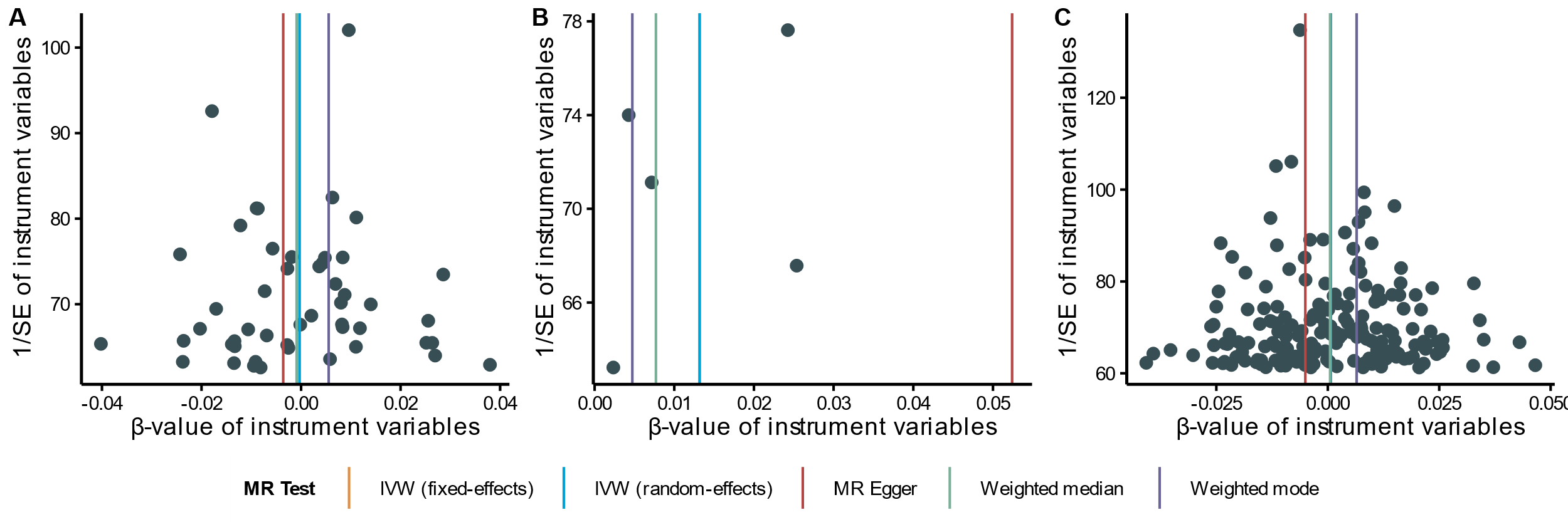


Funnel plots with colored vertical lines representing total MR estimation of causal associations between (A) computer using and colon cancer, (B) driving and colon cancer, (C) television watching and colon cancer.

### eFigure 82. Leave-one-out plots of leisure sedentary behaviors and colon cancer


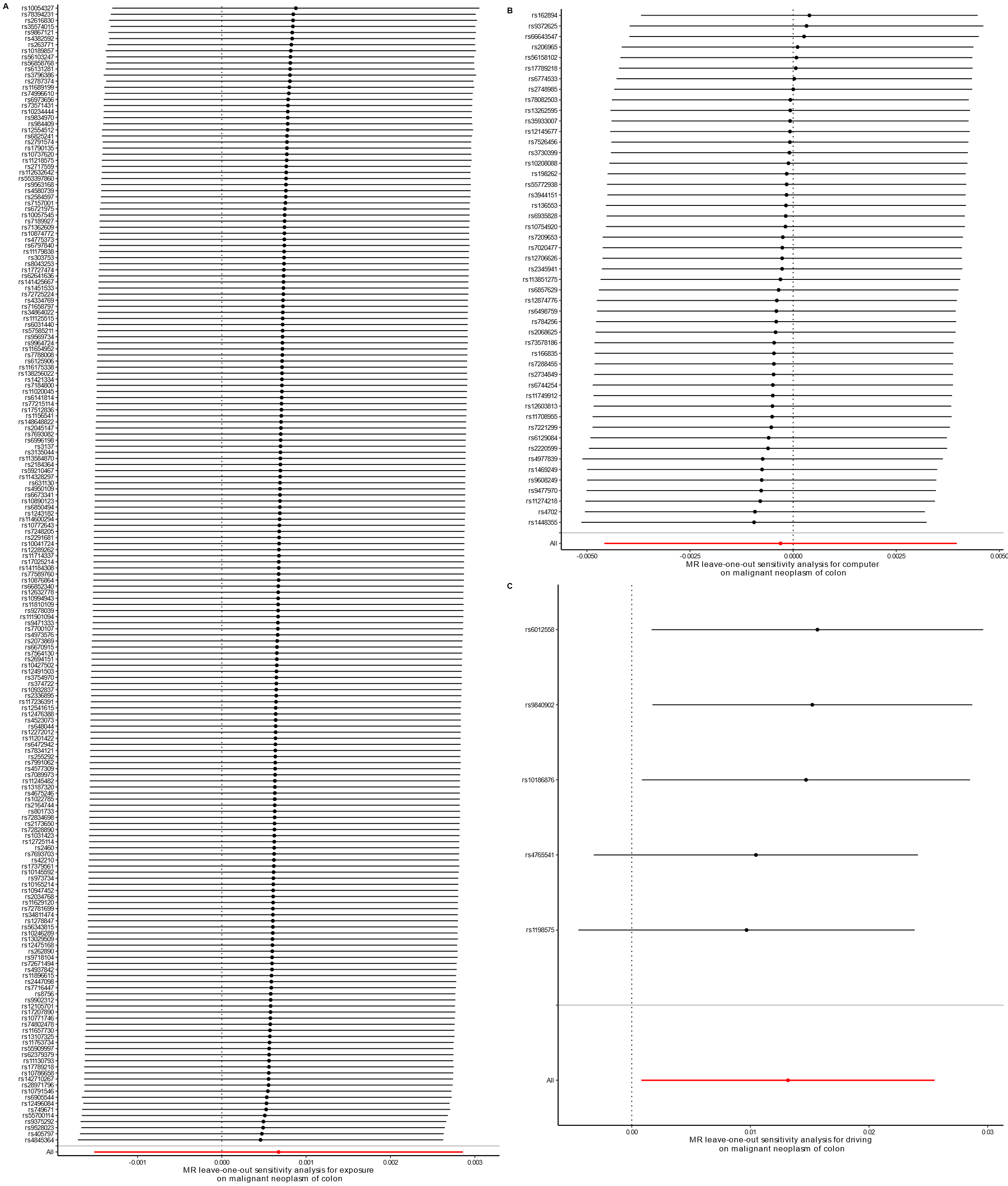


Leave-one-out plot of Mendelian randomization sensitivity analysis between (A) television watching and colon cancer, (B) computer using and colon cancer, (C) driving and colon cancer.

### eFigure 83. Forest plots of single-SNP analysis of leisure sedentary behaviors and colon cancer


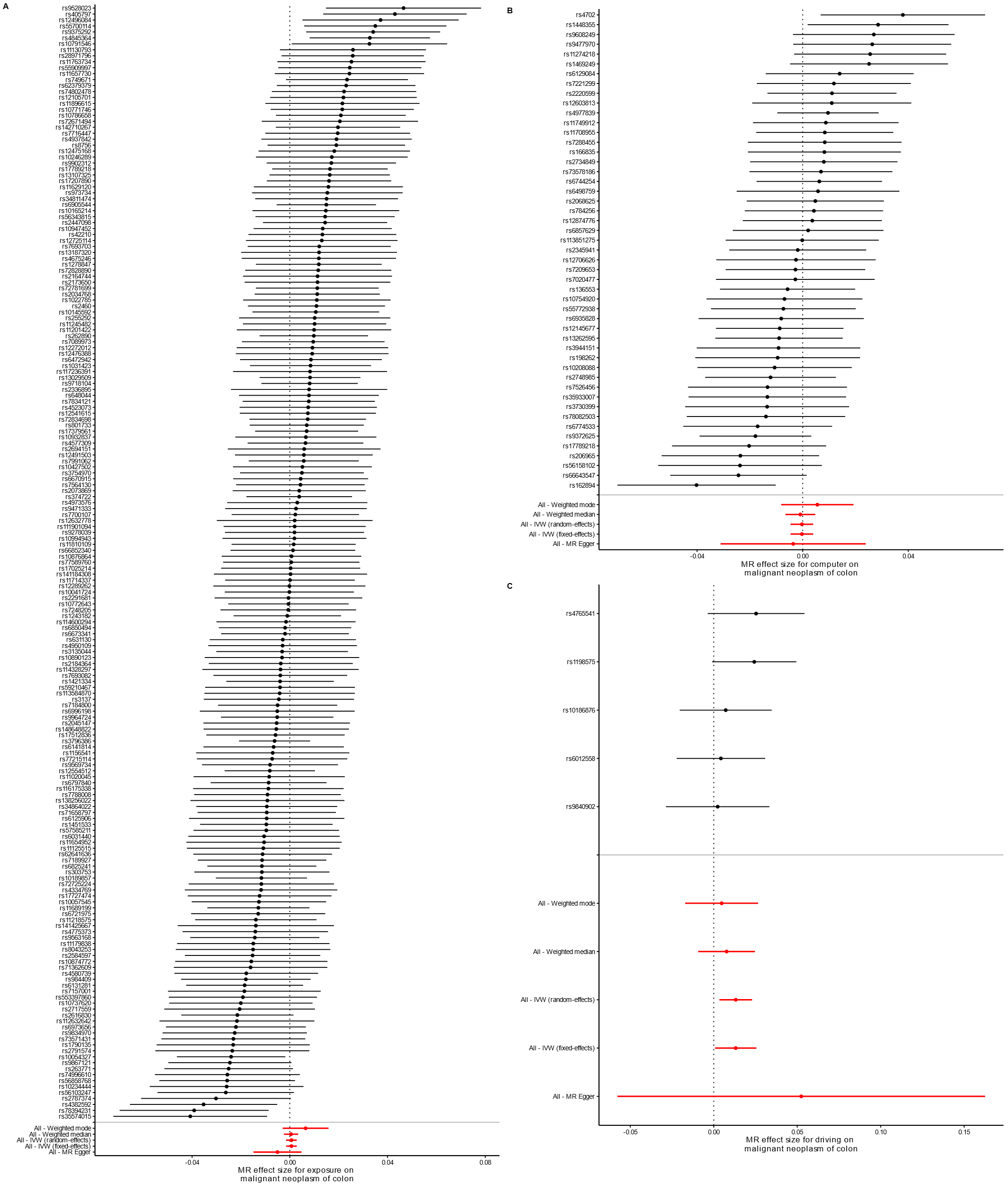


The MR single-SNP analysis plots the Wald estimate of causal association between (A) television watching and colon cancer, (B) computer using and colon cancer, (C) driving and colon cancer.

## eFigures of thyroid cancer

### eFigure 84. Scatter plots of leisure sedentary behaviors and thyroid cancer


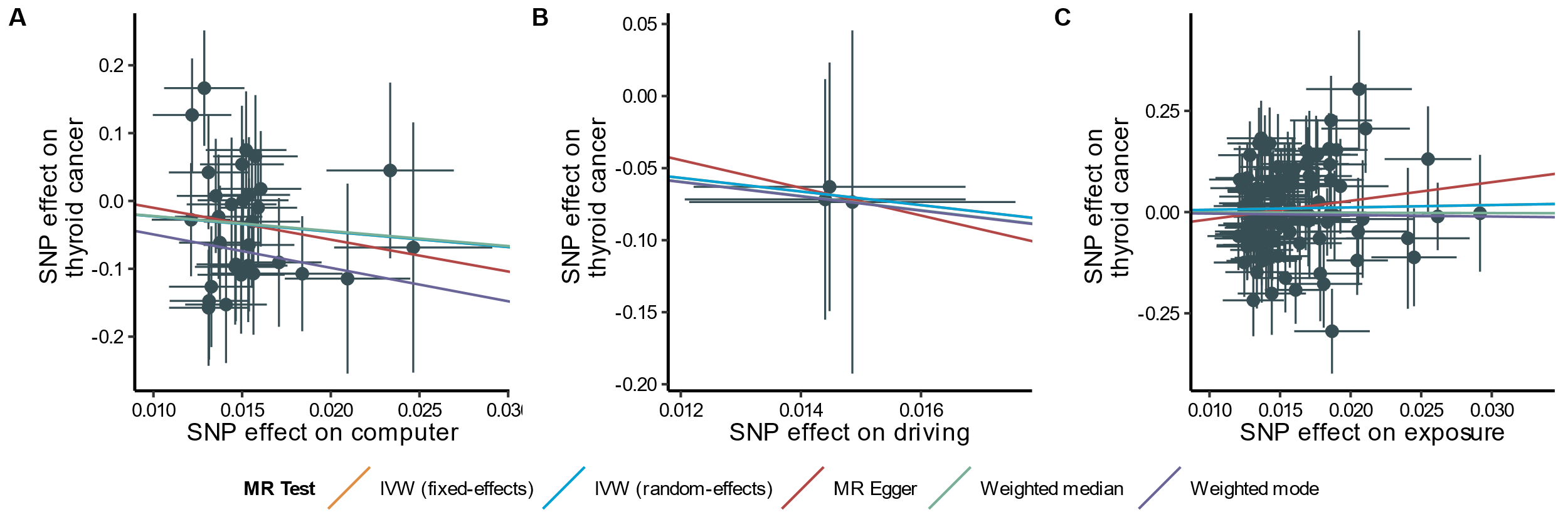


Scatter plots with colored lines representing results of each mendelian randomization sensitivity analysis between (A) computer using and thyroid cancer, (B) driving and thyroid cancer, (C) television watching and thyroid cancer.

### eFigure 85. Funnel plots of leisure sedentary behaviors and thyroid cancer


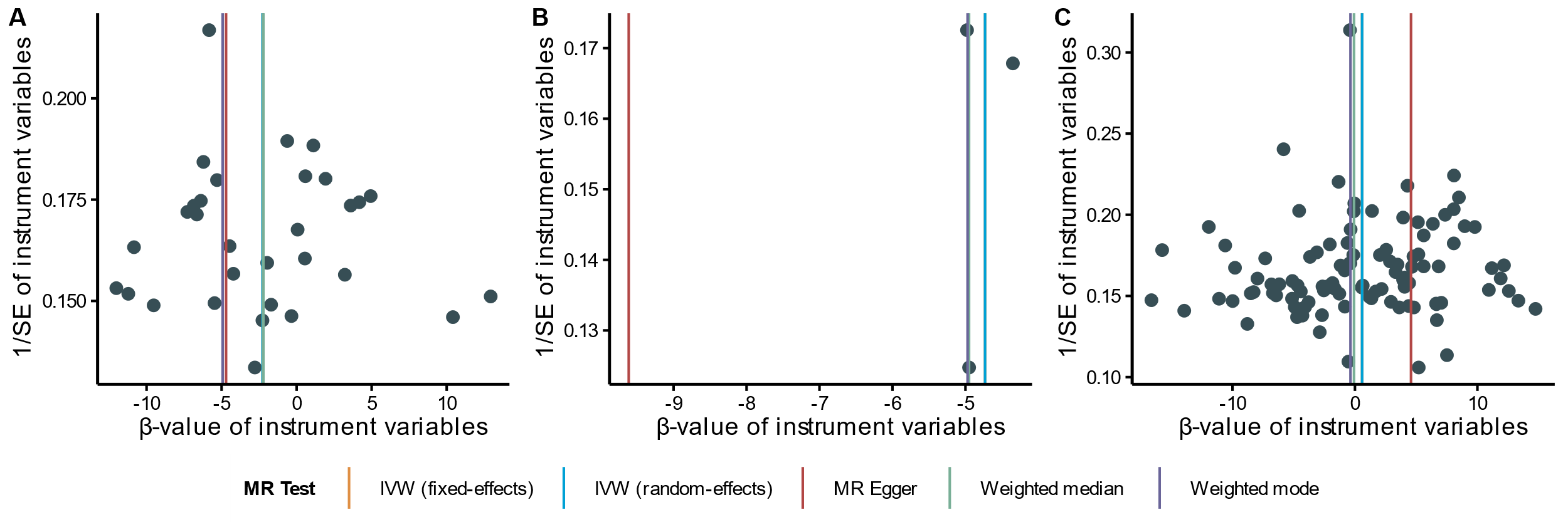


Funnel plots with colored vertical lines representing total MR estimation of causal associations between (A) computer using and thyroid cancer, (B) driving and thyroid cancer, (C) television watching and thyroid cancer.

### eFigure 86. Leave-one-out plots of leisure sedentary behaviors and thyroid cancer


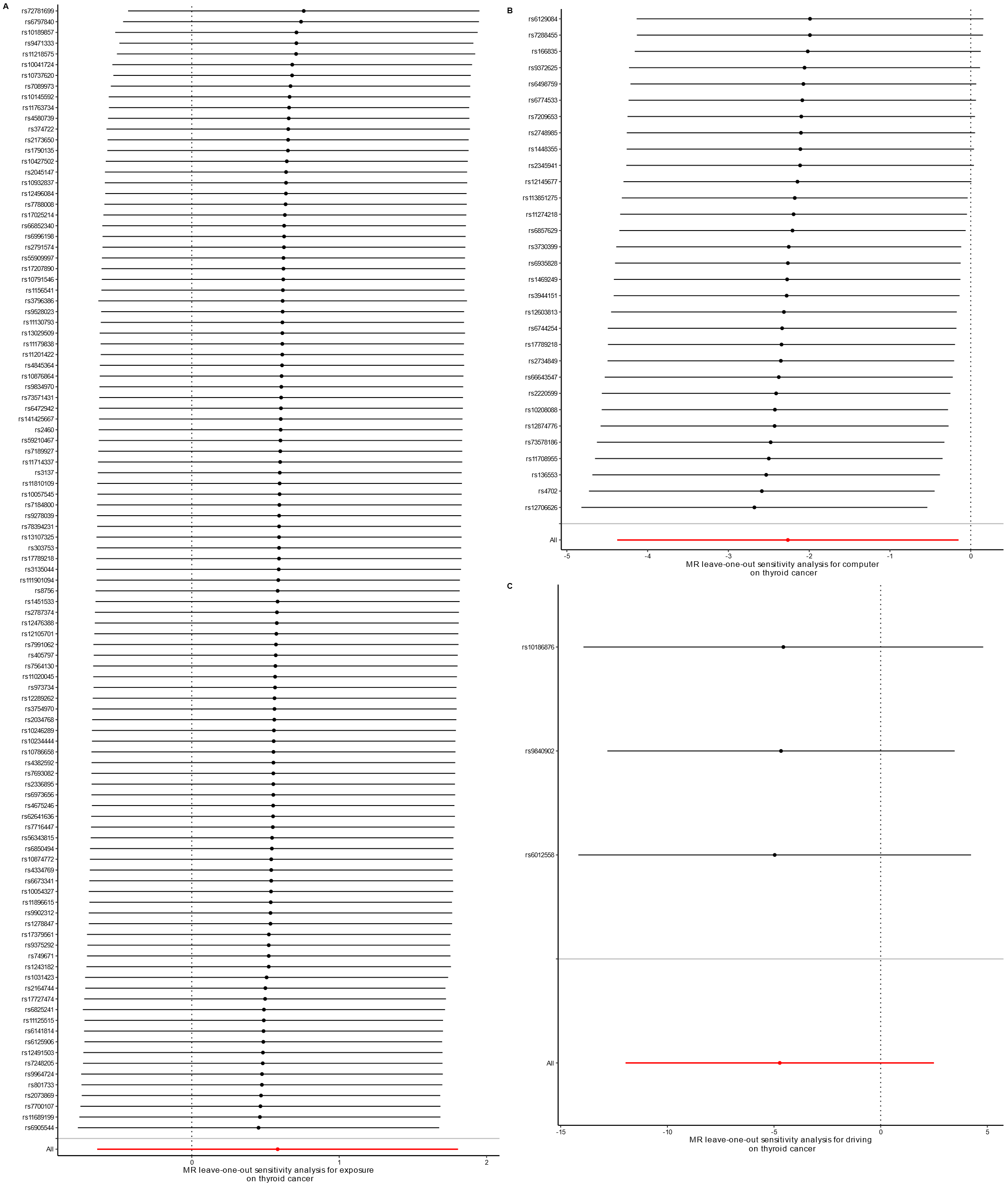


Leave-one-out plot of Mendelian randomization sensitivity analysis between (A) television watching and thyroid cancer, (B) computer using and thyroid cancer, (C) driving and thyroid cancer.

### eFigure 87. Forest plots of single-SNP analysis of leisure sedentary behaviors and thyroid cancer


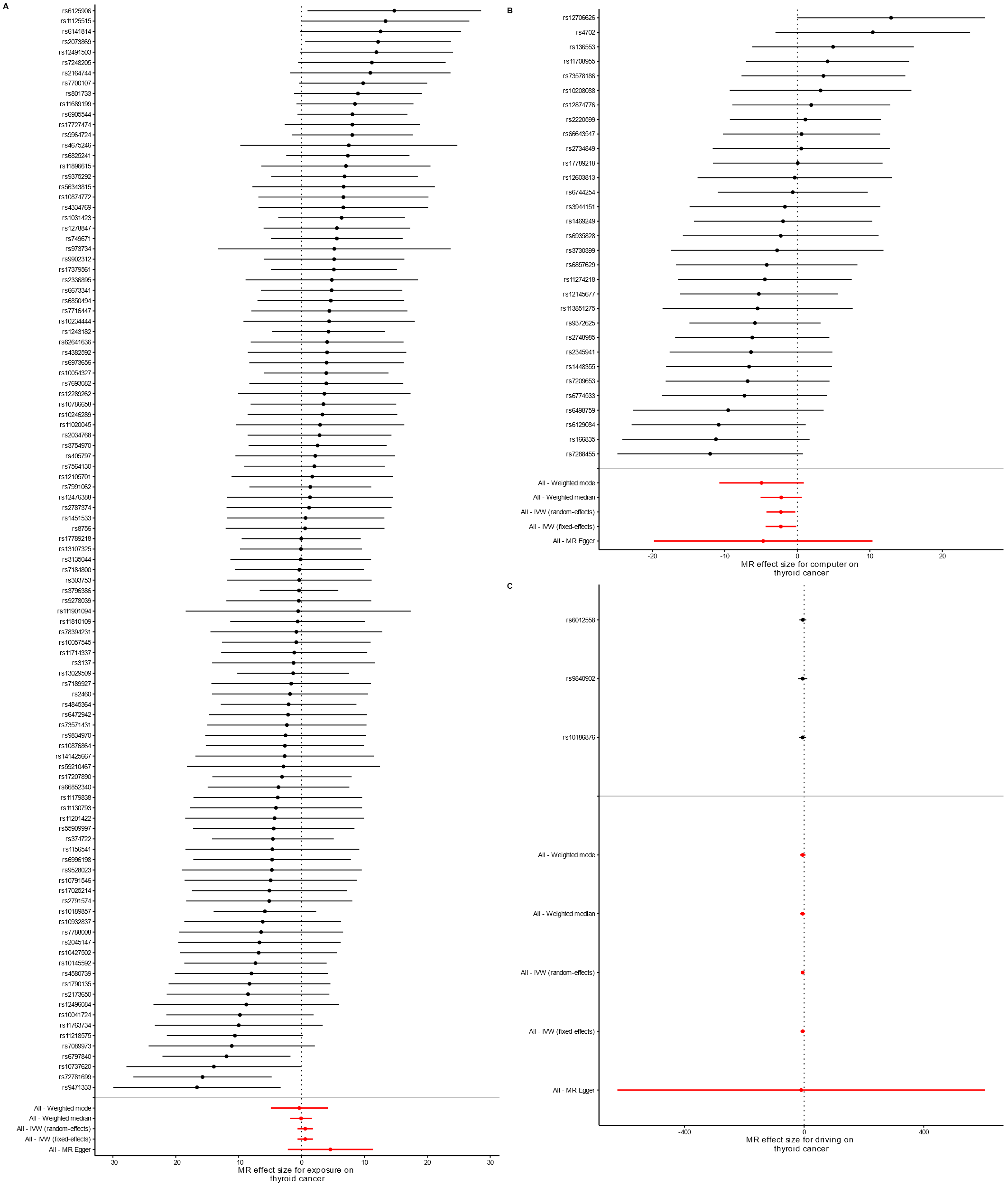


The MR single-SNP analysis plots the Wald estimate of causal association between (A) television watching and thyroid cancer, (B) computer using and thyroid cancer, (C) driving and thyroid cancer.

## eFigures of melanoma

### eFigure 88. Scatter plots of leisure sedentary behaviors and melanoma


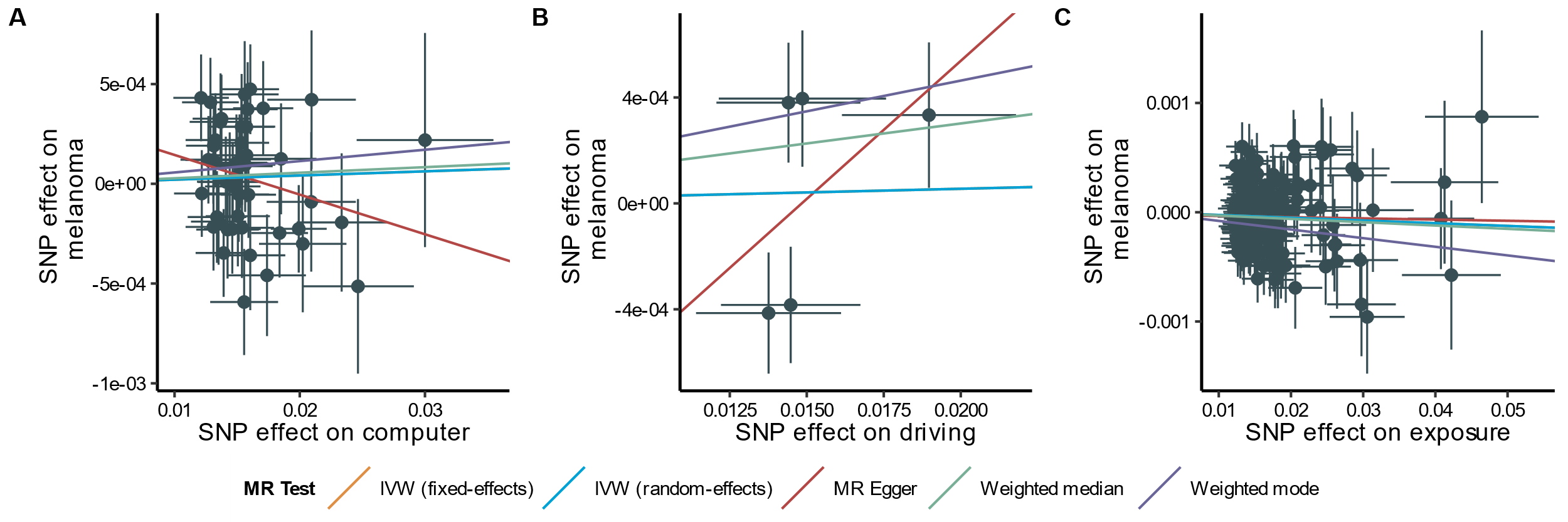


Scatter plots with colored lines representing results of each mendelian randomization sensitivity analysis between (A) computer using and melanoma, (B) driving and melanoma, (C) television watching and melanoma.

### eFigure 89. Funnel plots of leisure sedentary behaviors and melanoma


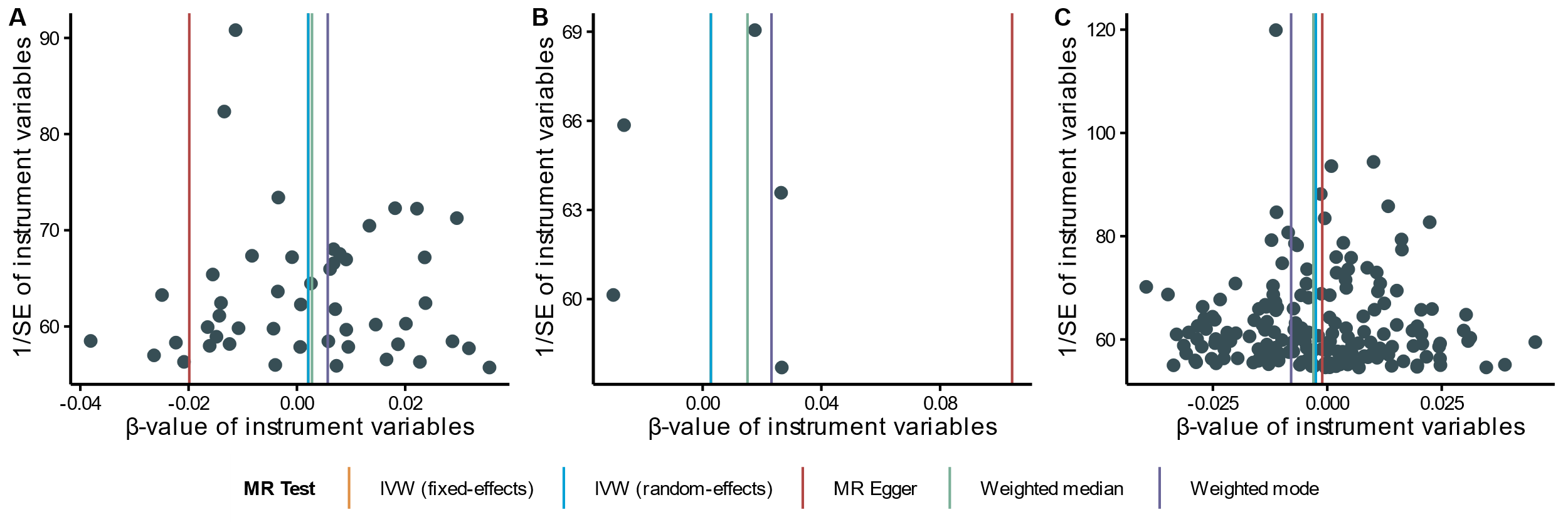


Funnel plots with colored vertical lines representing total MR estimation of causal associations between (A) computer using and melanoma, (B) driving and melanoma, (C) television watching and melanoma.

### eFigure 90. Leave-one-out plots of leisure sedentary behaviors and melanoma


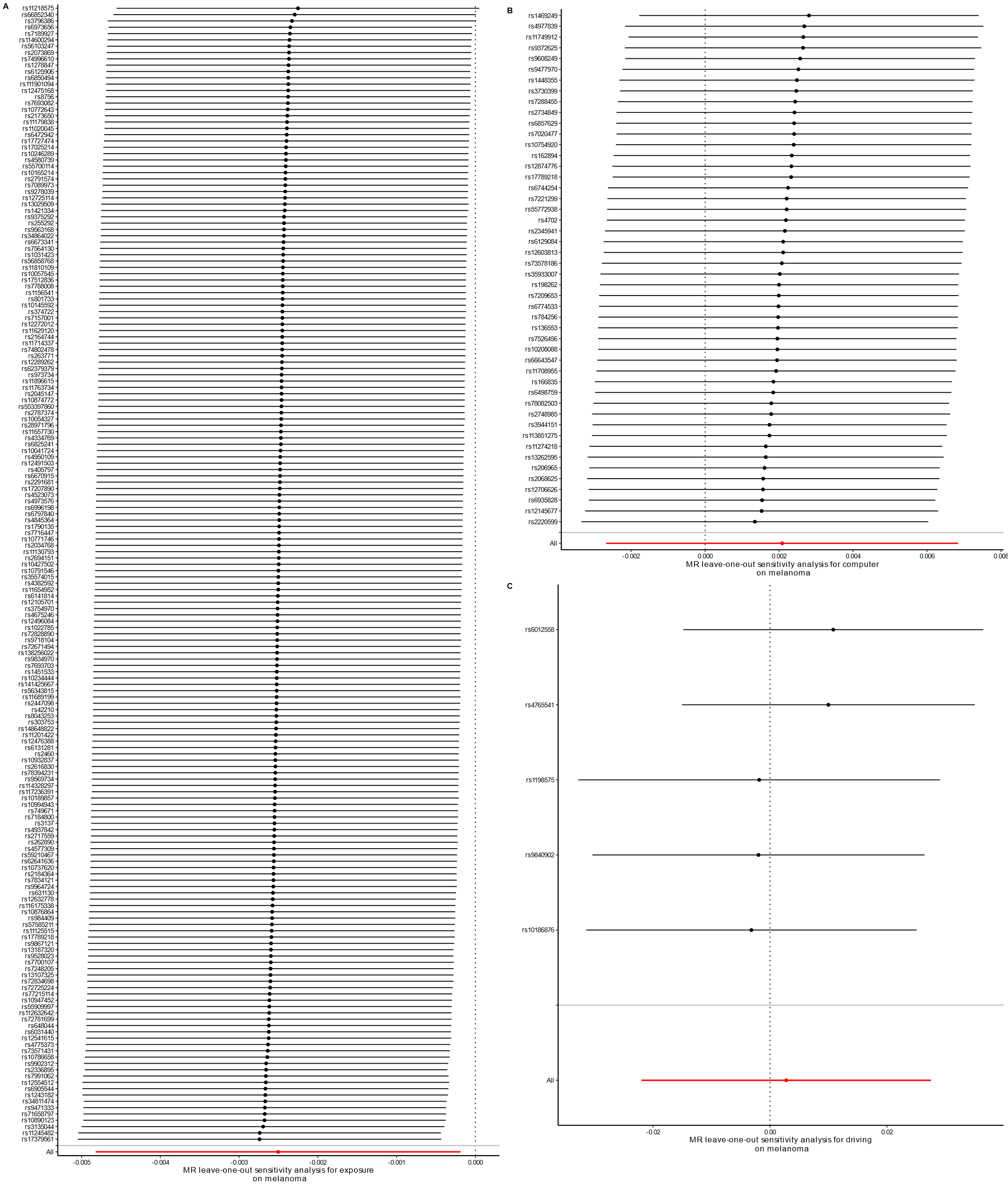


Leave-one-out plot of Mendelian randomization sensitivity analysis between (A) television watching and melanoma, (B) computer using and melanoma, (C) driving and melanoma.

### eFigure 91. Forest plots of single-SNP analysis of leisure sedentary behaviors and melanoma


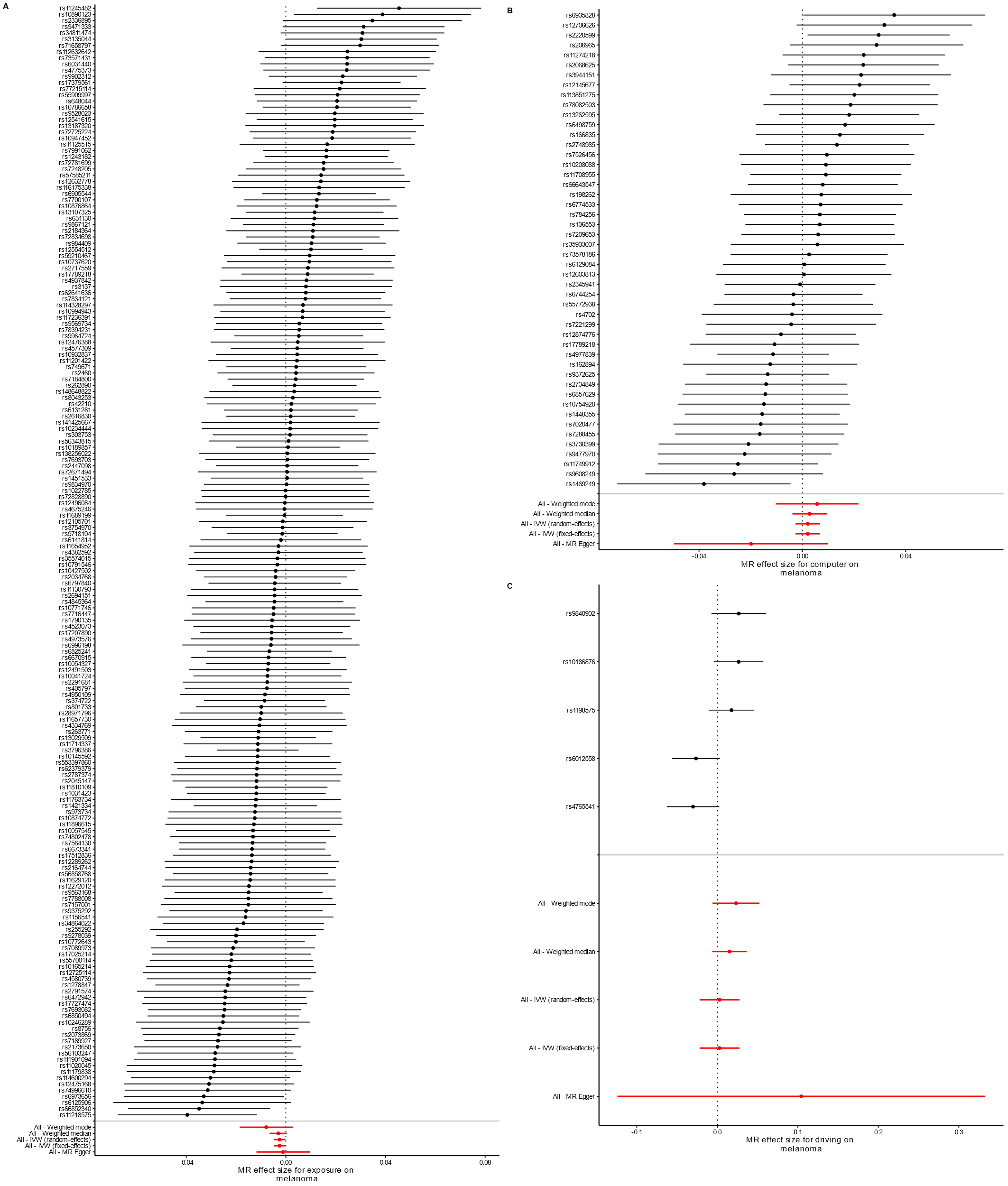


The MR single-SNP analysis plots the Wald estimate of causal association between (A) television watching and melanoma, (B) computer using and melanoma, (C) driving and melanoma.

## eFigures of bladder cancer

### eFigure 92. Scatter plots of leisure sedentary behaviors and bladder cancer


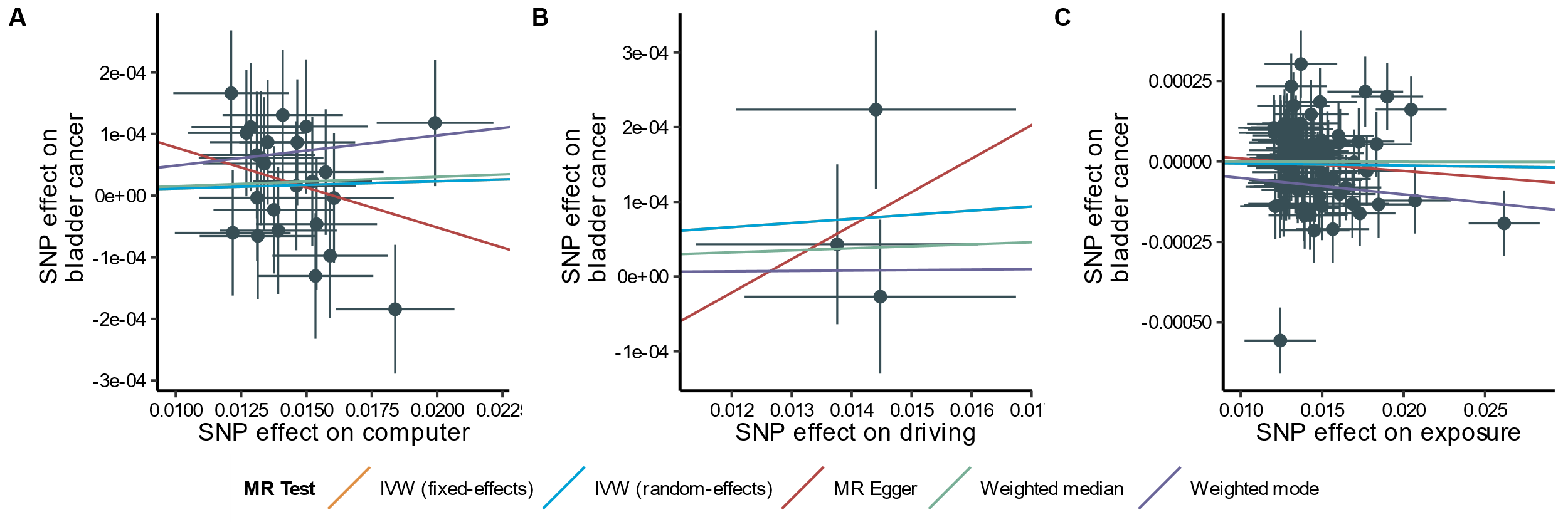


Scatter plots with colored lines representing results of each mendelian randomization sensitivity analysis between (A) computer using and bladder cancer, (B) driving and bladder cancer, (C) television watching and bladder cancer.

### eFigure 93. Funnel plots of leisure sedentary behaviors and bladder cancer


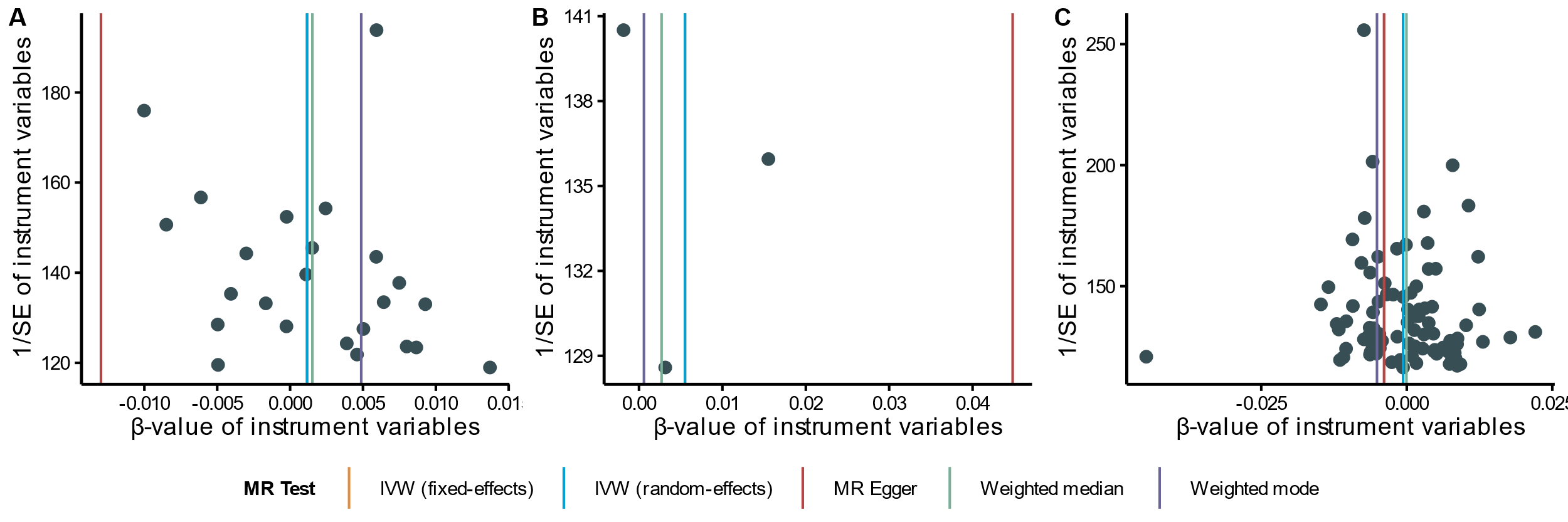


Funnel plots with colored vertical lines representing total MR estimation of causal associations between (A) computer using and bladder cancer, (B) driving and bladder cancer, (C) television watching and bladder cancer.

### eFigure 94. Leave-one-out plots of leisure sedentary behaviors and bladder cancer


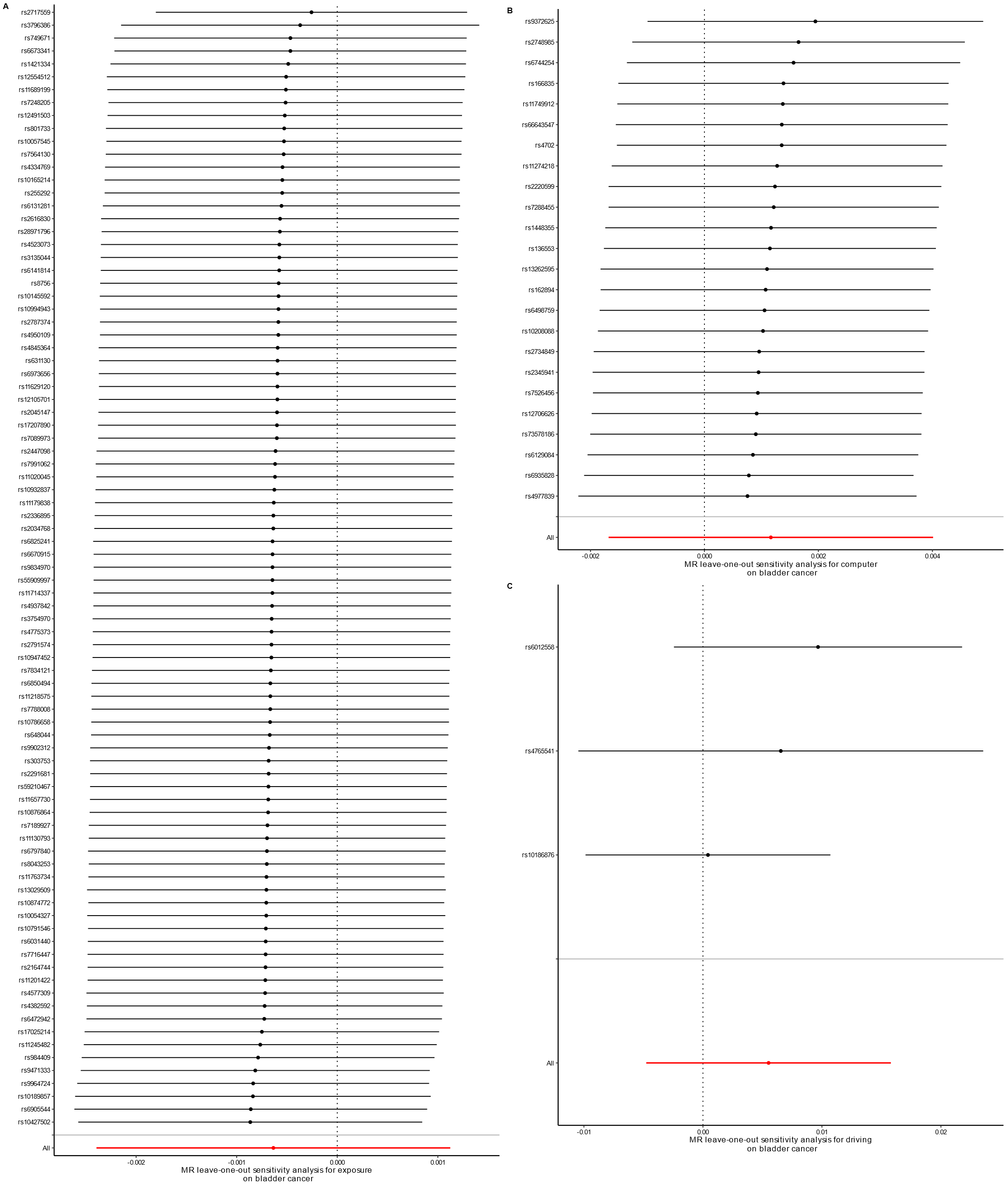


Leave-one-out plot of Mendelian randomization sensitivity analysis between (A) television watching and bladder cancer, (B) computer using and bladder cancer, (C) driving and bladder cancer.

### eFigure 95. Forest plots of single-SNP analysis of leisure sedentary behaviors and bladder cancer


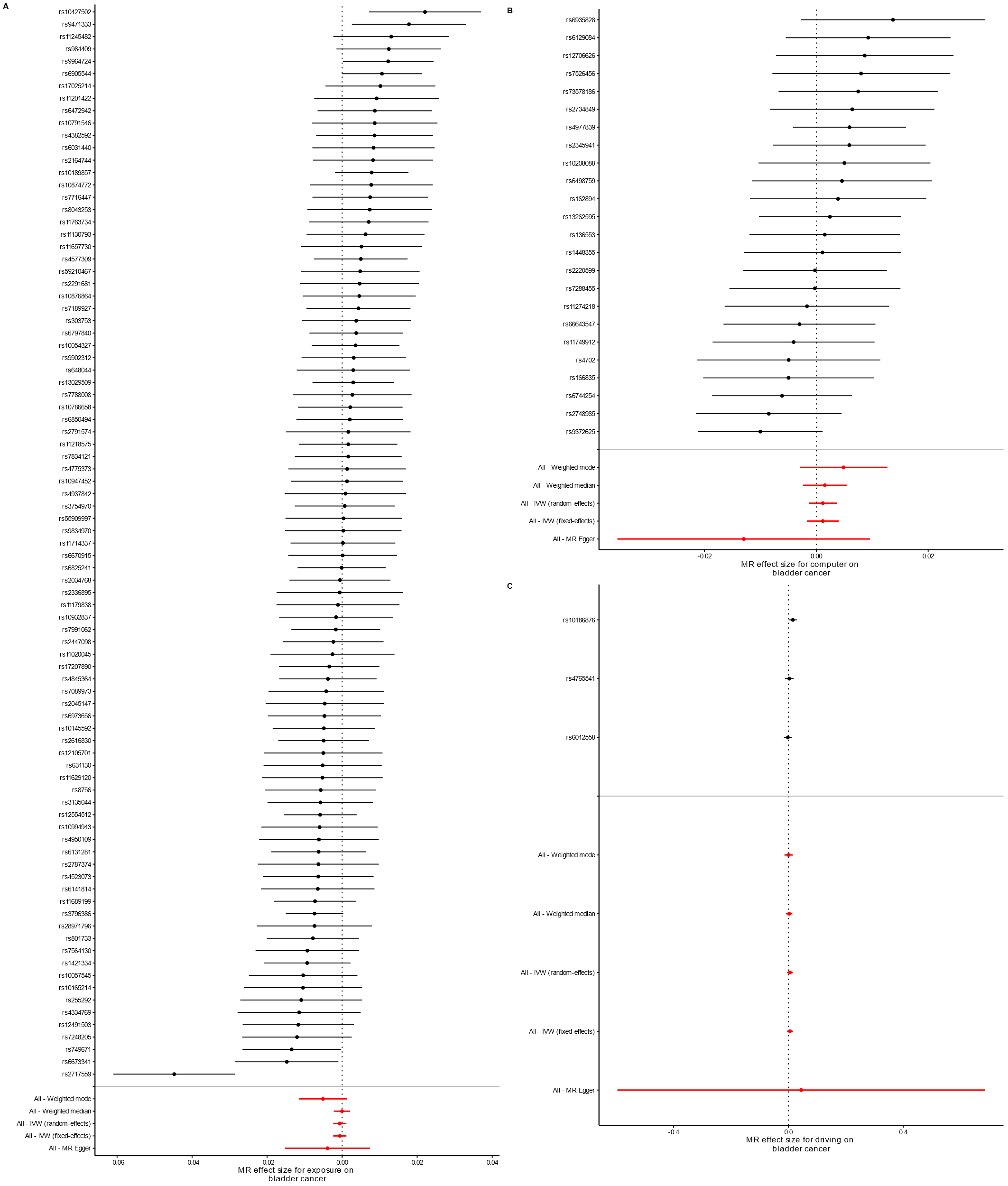


The MR single-SNP analysis plots the Wald estimate of causal association between (A) television watching and bladder cancer, (B) computer using and bladder cancer, (C) driving and bladder cancer

## eFigures of glioma

### eFigure 96. Scatter plots of leisure sedentary behaviors and glioma


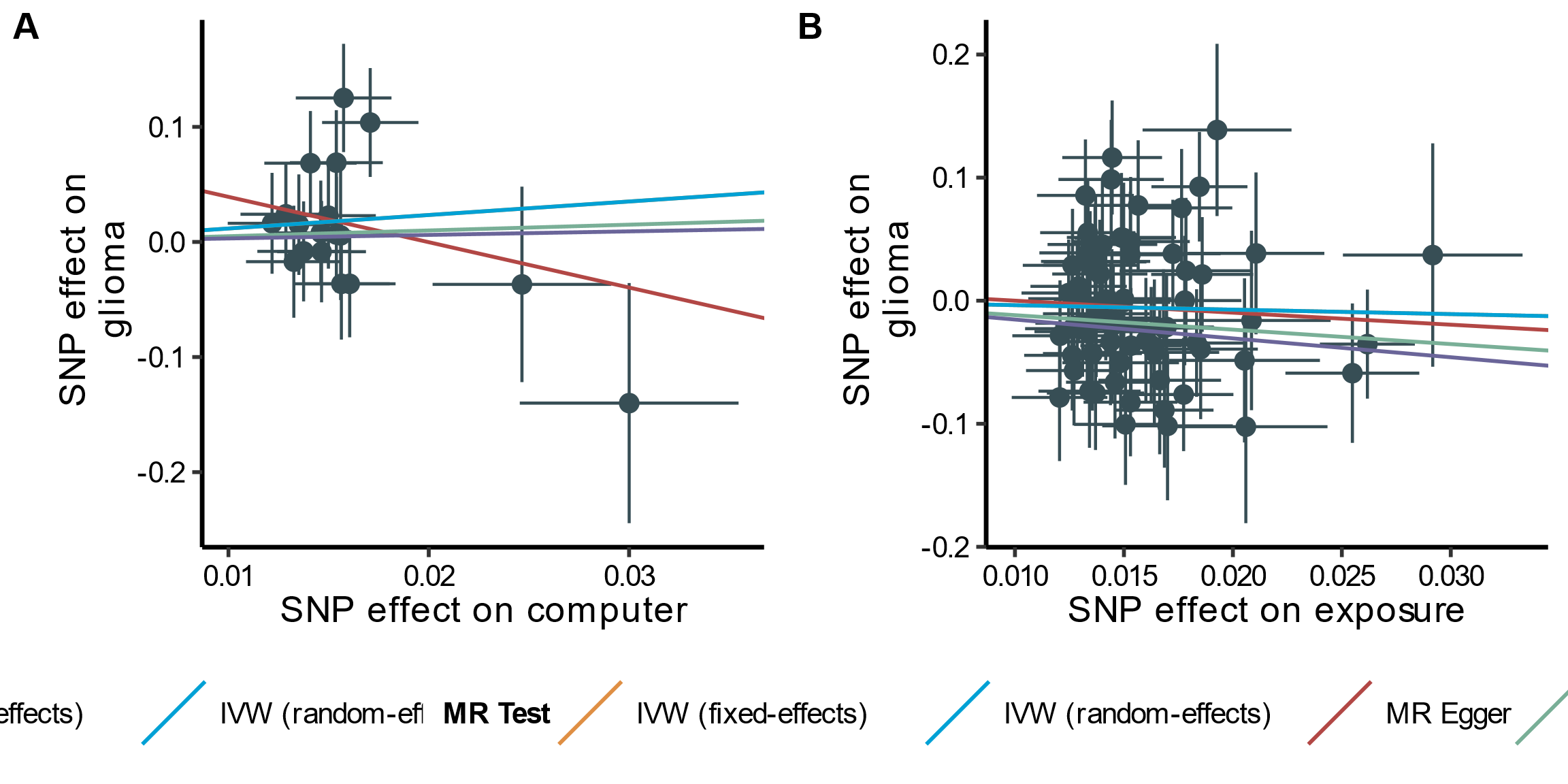


Scatter plots with colored lines representing results of each mendelian randomization sensitivity analysis between (A) computer using and glioma, (B) television watching and glioma.

### eFigure 97. Funnel plots of leisure sedentary behaviors and glioma


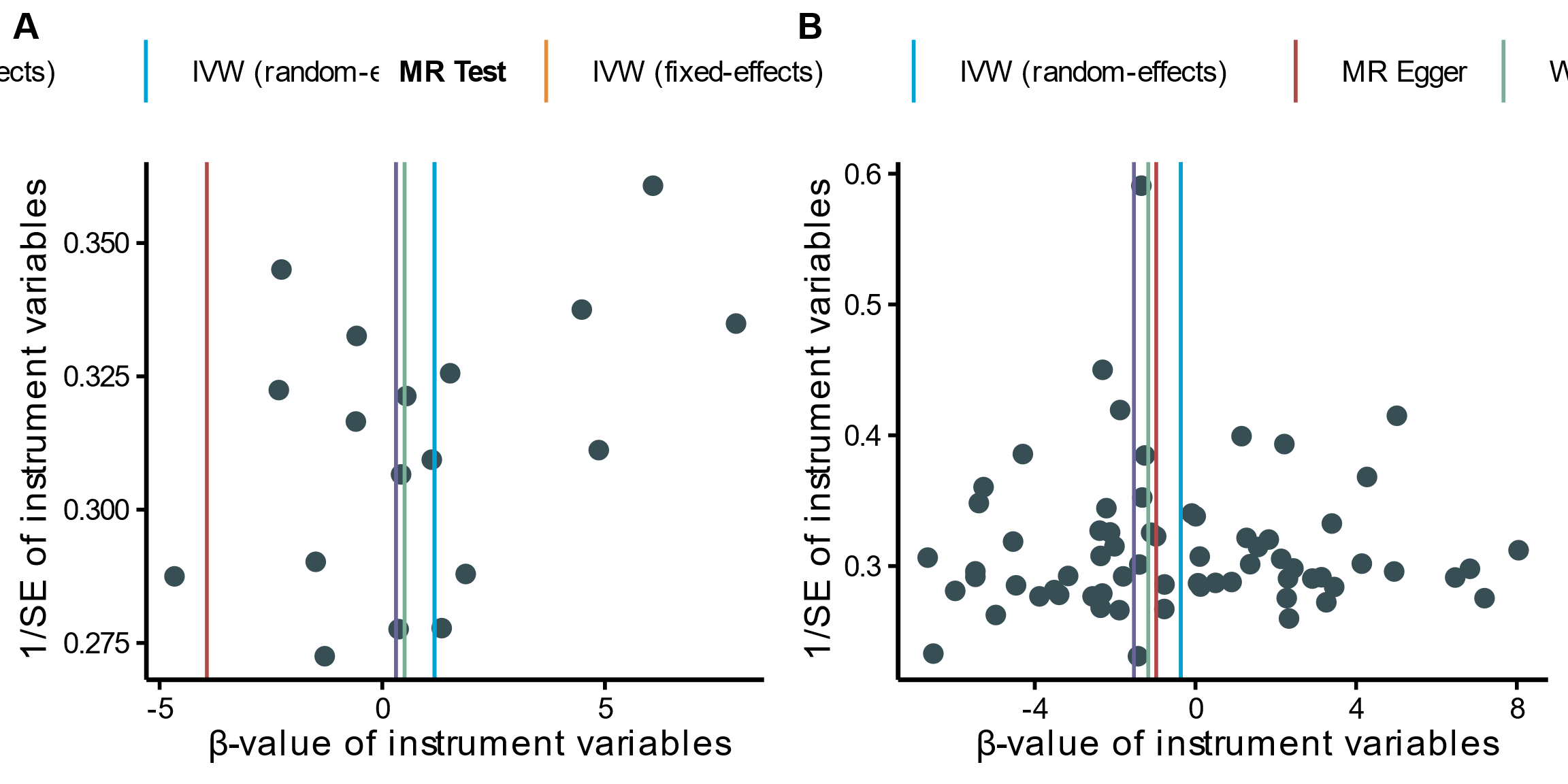


Funnel plots with colored vertical lines representing total MR estimation of causal associations between (A) computer using and glioma, (B) television watching and glioma.

### eFigure 98. Leave-one-out plots of leisure sedentary behaviors and glioma


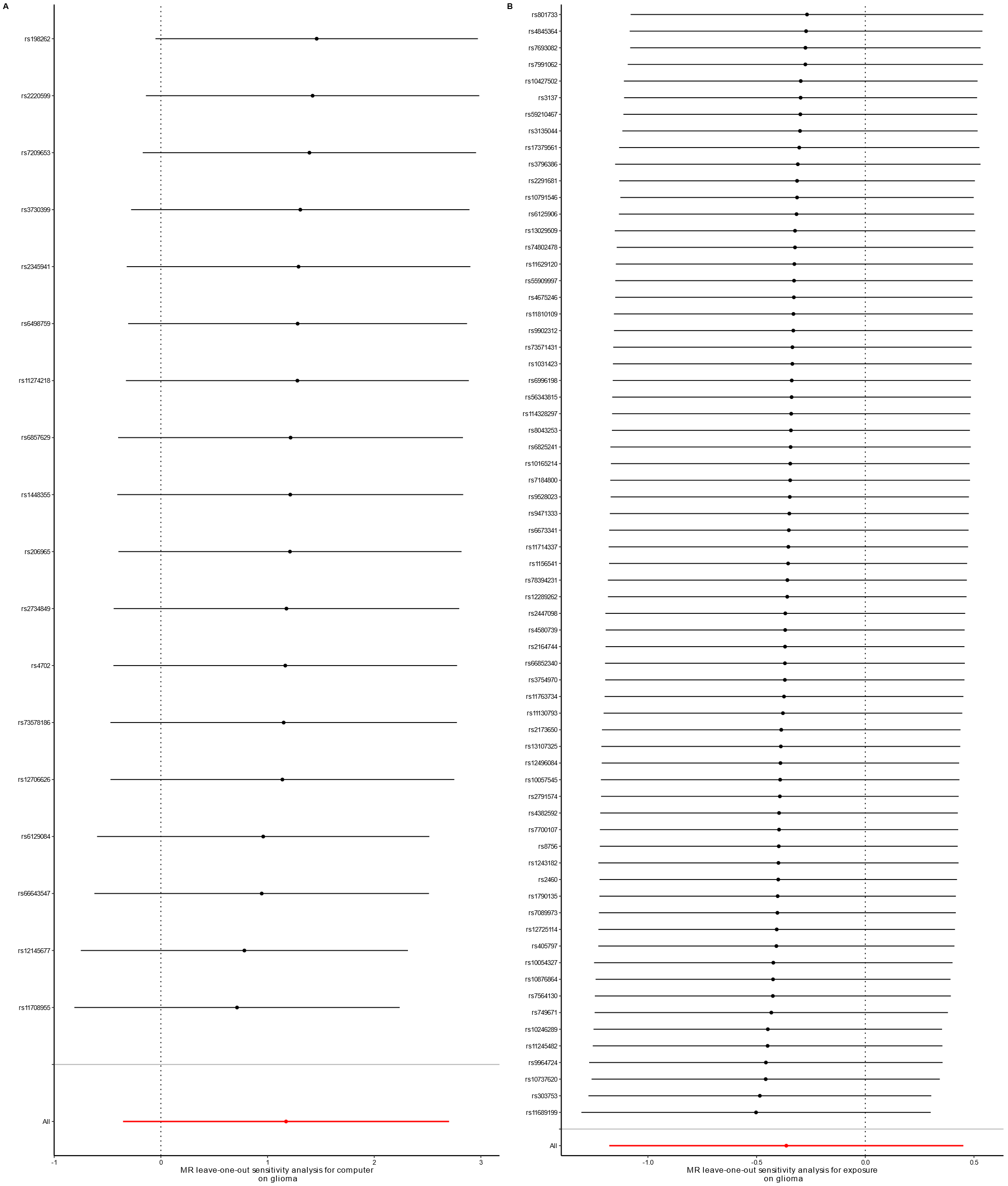


Leave-one-out plot of Mendelian randomization sensitivity analysis between (A) computer using and glioma, (B) television watching and glioma.

### eFigure 99. Forest plots of single-SNP analysis of leisure sedentary behaviors and glioma


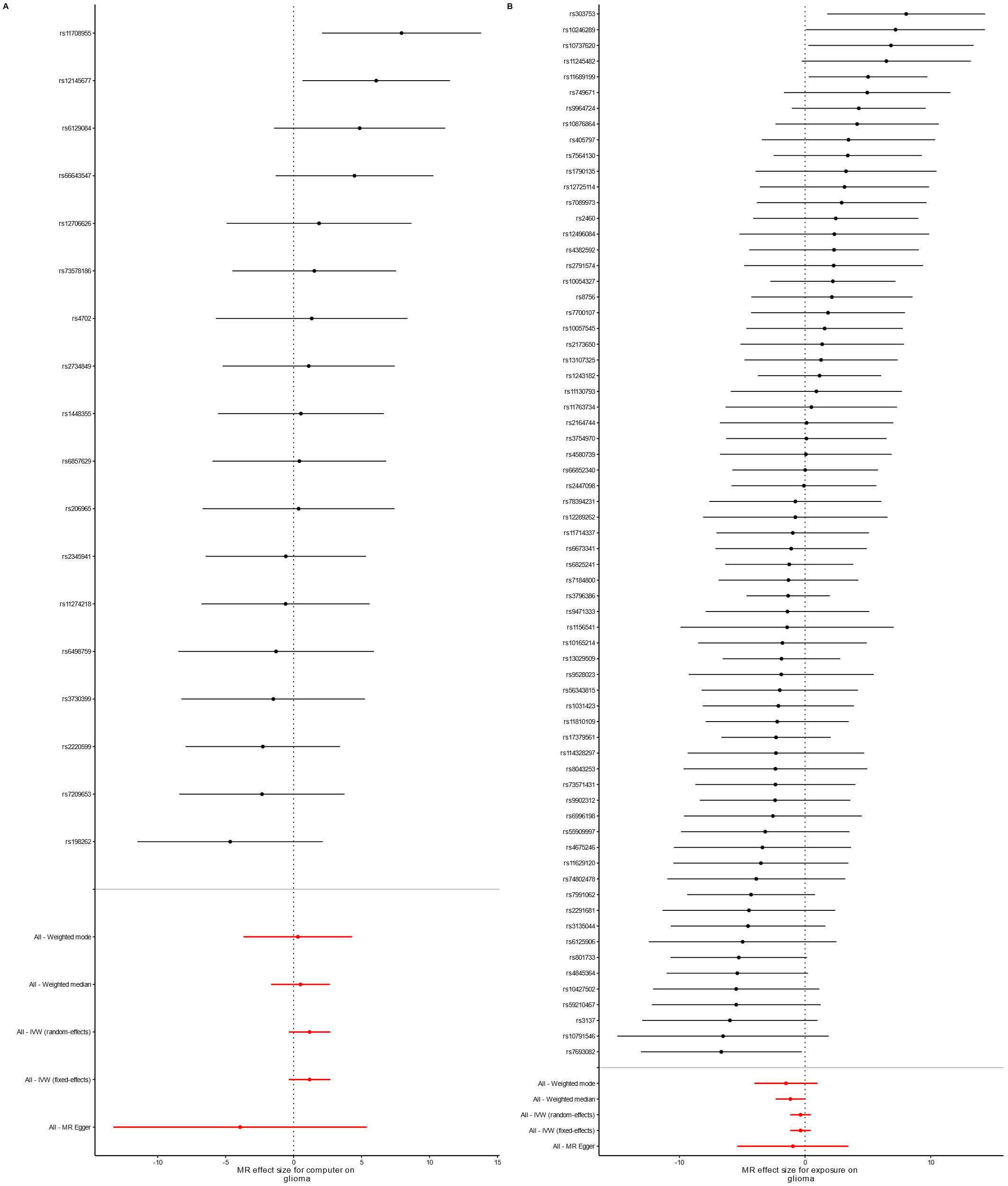


The MR single-SNP analysis plots the Wald estimate of causal association between (A) computer using and glioma, (B) television watching and glioma
